# Proteomic and transcriptomic analysis of heart failure due to volume overload in a rat aorto-caval fistula model provides support for new potential therapeutic targets - monoamine oxidase A and transglutaminase 2

**DOI:** 10.1186/1477-5956-9-69

**Published:** 2011-11-11

**Authors:** Jiri Petrak, Jana Pospisilova, Miroslava Sedinova, Petr Jedelsky, Lucie Lorkova, Ondrej Vit, Michal Kolar, Hynek Strnad, Jan Benes, David Sedmera, Ludek Cervenka, Vojtech Melenovsky

**Affiliations:** 1 Institute of Pathological Physiology, First Faculty of Medicine, Charles University, Prague, Czech Republic; 2 Faculty of Science, Charles University, Prague, Czech Republic; 3 Institute of Molecular Genetics, Academy of Sciences of the Czech Republic, Prague, Czech Republic; 4 Department for Experimental Medicine and Department of Cardiology, Institute for Clinical and Experimental Medicine-IKEM, Prague, Czech Republic; 5 Institute of Anatomy, First Faculty of Medicine, Charles University, Prague, Czech Republic; 6 Institute of Physiology, Academy of Sciences of the Czech Republic, Prague, Czech Republic; 7 Department of Physiology, 2nd Medical Faculty, Charles University, Prague, Czech Republic

**Keywords:** Heart failure, hypertrophy, annexins, monoamine oxidase, transglutaminase

## Abstract

**Background:**

Chronic hemodynamic overloading leads to heart failure (HF) due to incompletely understood mechanisms. To gain deeper insight into the molecular pathophysiology of volume overload-induced HF and to identify potential markers and targets for novel therapies, we performed proteomic and mRNA expression analysis comparing myocardium from Wistar rats with HF induced by a chronic aorto-caval fistula (ACF) and sham-operated rats harvested at the advanced, decompensated stage of HF.

**Methods:**

We analyzed control and failing myocardium employing iTRAQ labeling, two-dimensional peptide separation combining peptide IEF and nano-HPLC with MALDI-MS/MS. For the transcriptomic analysis we employed Illumina RatRef-12v1 Expression BeadChip.

**Results:**

In the proteomic analysis we identified 2030 myocardial proteins, of which 66 proteins were differentially expressed. The mRNA expression analysis identified 851 differentially expressed mRNAs.

**Conclusions:**

The differentially expressed proteins confirm a switch in the substrate preference from fatty acids to other sources in the failing heart. Failing hearts showed downregulation of the major calcium transporters SERCA2 and ryanodine receptor 2 and altered expression of creatine kinases. Decreased expression of two NADPH producing proteins suggests a decreased redox reserve. Overexpression of annexins supports their possible potential as HF biomarkers. Most importantly, among the most up-regulated proteins in ACF hearts were monoamine oxidase A and transglutaminase 2 that are both potential attractive targets of low molecular weight inhibitors in future HF therapy.

## Background

Heart failure (HF) is a major cause of human morbidity and mortality with increasing prevalence worldwide, affecting 2-4% of the adult European population [1]. HF is a complex syndrome, resulting from an impaired ability of the diseased heart to maintain adequate effective cardiac output [2]. Typical signs and symptoms of chronic HF are shortness of breath, cough, accumulation of fluids in the lungs and other tissues, fatigue, limitations on physical activity and arrhythmia [2]. The prognosis for affected individuals is poor and 50% of chronic HF patients die within 4 years of the initial diagnosis [1]. Despite substantial progress in deciphering individual processes involved in the initiation and gradual progression of HF [3], our understanding of the underlying molecular causes of cardiomyocyte dysfunction is still very limited. The molecular phenotype of heart failure has been associated with the altered expression of proteins involved in energy metabolism, membrane excitation, calcium-mediated excitation-contraction coupling, force transduction and with myofilament contraction or relaxation [3]. Studies of the molecular mechanisms of HF in humans are undermined by multifactor etiology of cardiac dysfunction, by confounding co-morbid conditions and also by a lack of appropriate healthy controls. These obstacles can be avoided in experimental animal models. In rodents, experimental HF is most often induced by myocardial infarction (ligation of the proximal left coronary artery) or by pressure overload (banding of the proximal aorta). As recently demonstrated the molecular responses to volume and pressure overload appear to differ [4].

HF induced by chronic volume overload has been studied less, despite such overload due to valve insufficiency being relatively common among HF patients [5]. Volume overload due to a surgically created aorto-caval fistula (ACF) in rats is a well defined model of chronic HF [6-8], which mimics the gradual transition of asymptomatic cardiac hypertrophy into symptomatic HF. The creation of an ACF leads to increased cardiac output and eccentric ventricular hypertrophy that remains asymptomatic for 8-10 weeks. Because most of cardiac output is shunted into the inferior vena cava, the effective cardiac output is reduced. leading to renal hypoperfusion [7], neurohumoral activation, and sodium/water retention [8]. Elevated cardiac filling pressures further contribute to cardiac overload [9-11]. By these mechanisms, HF gradually develops [8].

To better elucidate the molecular pathophysiology of HF due to ACF, and to identify potential molecular targets for novel therapies, we performed a proteomic analysis of the left ventricle myocardium from ACF animals with signs of HF (150 days after fistula creation) and control (sham-operated) rats. We used a shot-gun approach that combines iTRAQ labeling chemistry [12] with two-dimensional separation of peptides by isoelectric focusing on immobilized pH gradients (IEF-IPG) [13] followed by nano-HPLC and MALDI mass spectrometry. The myocardial samples were also subjected to mRNA microarray expression analysis.

## Materials and methods

### The chronic HF model

HF due to volume overload was induced in male Wistar rats (300-350 g) by creating an aorto-caval fistula (ACF) using a 1.2 mm needle from laparotomy under general anesthesia, as described previously [6,7]. Control sham-operated animals underwent the same procedure, but without creating an ACF. The animals were kept on a 12/12-hour light/dark cycle, and fed a normal salt/protein diet (0.45% NaCl, 19-21% protein, SEMED, CR). The investigation conformed to the NIH Guide for the care and use of laboratory animals (NIH Publication No. 85-23, 1996), Animal protection laws of the Czech Republic (311/1997) and was approved by the Ethics Committee of IKEM (305/09/1390 from 25. March 2008).

### Echocardiography and hemodynamics

Examinations were performed under general anesthesia (ketamine+midazolam mixture) at the study end (150 days after ACF creation) prior to harvesting of heart tissue. Echocardiography was performed with a 10 MHz probe (Vivid System 5, GE, USA). End-systolic and end-diastolic left ventricle (LV) volumes were derived by the cubic equation and stroke volume as their difference. Hemodynamics was measured with a 2F micro-manometer catheter (Millar Instruments) inserted into the aorta and LV via the carotid artery, connected to a Powerlab 8 platform for off-line analysis with LabChart software (ADInstruments, Germany). The presence of ACF was verified by laparotomy and the animals were killed by exsanguination. After removal, hearts were immediately perfused with ice-cold St. Thomas cardioplegic solution administered into the aortic root. The organs were weighted and normalized to body weight.

### Morphological examination

Perfused hearts were fixed with 4% paraformaldehyde in phosphate buffer saline (PBS). After 24 h of immersion in the same fixative, the hearts were rinsed in PBS and processed through ascending series of saccharose prior to embedding into Tissue-Tek OCT medium. The blocks were then cut on cryomicrotome at 12 micrometers thickness. Guide series were stained by hematoxylin-eosin with Alcian blue. Sister sections were then stained with Picrosirius Red. The slides were finally washed with distilled water and dehydrated in ascending ethanol series, cleared in xylene, and mounted in Depex medium. Observation and photography were performed in transmitted and polarized light on an Olympus BX51 compound microscope.

### Myocardial sample preparation

Samples of mid-ventricular anterior free LV wall tissue were immediately harvested into liquid nitrogen and stored at -80°C until analysis. Frozen samples (ACF, n = 6 and controls, n = 6) were pulverized under liquid nitrogen and the samples were sub-pooled according to the following scheme: ACF1 (ACF rats #1,3,5), ACF2 (ACF rats #2,4,6), Sham1 (sham-operated rats #1,3,5), Sham2 (sham-operated rats #2,4,6). The pooled samples (10 mg) were extracted with 1 mL of NHT buffer (140 mM NaCl, 10 mM Hepes, 1.5% Triton X-100, pH 7.4) for 15 min on ice. Insoluble material was sedimented at 15 000 × g for 15 min and the protein concentration of the cleared supernatant was determined by the Bradford assay (Bio-Rad, CA). A 100 μg aliquot from each sample was precipitated overnight in cold acetone (-20°C). Precipitated proteins were sedimented at 15 000 × *g *at 4°C for 15 min.

### Protein digestion and iTRAQ labeling

Extracted and acetone-precipitated myocardial samples were reduced, alkylated, digested with trypsin and labeled with 114-117 iTRAQ chemistry according to the manufacturer's instructions (Applied Biosystems, UK). Labeling was performed as follows: "114" - ACF1, "115" - ACF2, "116" - Sham1, "117" - Sham2. Labeled samples 114-117 were then combined and the volume of the final sample was reduced to 40 μL in a SpeedVac Concentrator (Eppendorf, CR). In total, three independent analyses A, B and C of the ACF1, ACF2, Sham1 and Sham2 samples were performed including extraction, digestion, labeling, separation and MS analysis.

### IEF-IPG of peptides, extraction

Isoelectric focusing was performed on a Protean IEF cell (Bio-Rad, CA, USA) using 24 cm IPG strips (pH 4-7, Bio-Rad). Strips were rehydrated overnight in 450 μL of iTRAQ-labeled peptide mixture diluted with rehydration buffer (7 M urea, 2 M thiourea, 4% CHAPS, 60 mM DTT, 1% ampholytes and 0.002% bromophenol blue). IEF was carried out for 73 kVhr with maximum voltage not exceeding 6 kV, current limited to 50 μA per strip and temperature set to 20°C. After focusing, strips were briefly washed in water, cut into 32 pieces and peptides were extracted from individual strip pieces into 150 μL of 80% acetonitrile with 0.5% trifluoroacetic acid, for one hour at room temperature. The volume of all fractions was reduced to 5-10 μL by evaporation in the SpeedVac Concentrator and fractions were stored at -80°C.

### LC-MALDI

LC-MALDI analyses were performed on an Ultimate 3000 HPLC system (Dionex, Sunnyvale, USA) coupled to a Probot micro-fraction collector (Dionex). Extracted post-IEF fractions were loaded onto a PepMap 100 C18 RP column (3 μm particle size, 15 cm long, 75 μm internal diameter; Dionex) and separated by a gradient of 3% (v/v) acetonitrile, 0.1% (v/v) trifluoroacetic acid to 44% (v/v) acetonitrile, 0.1% (v/v) trifluoroacetic acid over a period of 113 min and from 44% to 80% ACN over the next 7 min. The flow rate was set to 300 nL/min. The eluate was mixed 1:3 with matrix solution (2 mg/mL α-cyano-4-hydroxycinnamic acid in 80% ACN) by the Probot micro-fraction collector prior to spotting onto a MALDI target (5 spots per minute). Spectra were acquired on a 4800 Plus MALDI TOF/TOF analyzer (AB Sciex) equipped with a Nd:YAG laser (355 nm, firing rate 200 Hz). All spots were first measured in MS mode from m/z 800 to 4,000 and then up to 15 strongest precursors were selected for MS/MS analysis which was performed with 1 kV collision energy and a collision cell operating pressure of 10^-6 ^Torr. Tandem mass spectra were processed with a 4000 Series Explorer with subtract baseline enabled (peak width 50), Gaussian smoothing enabled (filter width 5), minimum signal to noise 8, local noise window width 250 m/z, minimum peak width at full width half max 2.9 bins, cluster area signal to noise optimization enabled (threshold 15), and flag monoisotopic peaks enabled.

### Proteomic data analysis

Mass spectrometry data from all three parallel analyses A, B and C were merged and processed as a single dataset. Protein identification and quantitation were performed using Protein Pilot 3.0 (AB Sciex). MS/MS spectra were searched against the *Rattus norvegicus *sequences assembly downloaded from GenBank (http://www.ncbi.nlm.nih.gov/protein, 110 358 sequences, as of 06-Jan-2010) with the following settings: Trypsin digestion (semitryptic peptides allowed), methyl methanethiosulfonate modification of cysteines, iTRAQ 4-plex labeled peptides, instrument 4800, no special factors, default iTRAQ isotope correction settings, quantification, bias correction, background correction, biological modifications and thorough ID parameters selected. Probabilities of modifications were not altered. The detected protein threshold (unused protein score and confidence of results) was set to 2.0 and 99.0% and false discovery rate analysis was enabled. Proteins sharing a set of peptides were grouped automatically with the default Pro Group™ Algorithm. Ratios of iTRAQ were calculated with default Protein Pilot setting, Protein fold change (iTRAQ ratio for an individual protein) was calculated automatically by the Protein Pilot software as a weighted average of Log iTRAQ ratios determined for individual peptides belonging to the particular protein after background subtraction.

To estimate the false discovery rate (FDR) a decoy database search was performed. For each protein ratio the Protein Pilot reported the p-value and EF (error factor). To be considered as differentially expressed, individual proteins had to fulfill the following statistical criteria: p value<0.05, EF<2 and average iTRAQ ratio>1.5. In our experimental iTRAQ labeling scheme ("114" - ACF1, "115" - ACF2, "116" - Sham1, "117" - Sham2) a protein was considered differentially expressed only when the all three parameters were reached for all four ACF/Sham protein iTRAQ ratios (i.e for all ratios 116/115, 116/114, 117/115 and 117/114). The fold-change of differentially expressed proteins was calculated as the average value from the protein iTRAQ ratios reported by Protein Pilot.

### Western blotting

Myocardial protein samples (20 μg) were separated on 10 or 12% SDS-PAGE minigels in Tris-Glycine buffer. Electrophoresis was performed at a constant voltage 90 V. Proteins were then transferred to PVDF membranes (Milipore, MA, USA) in semi-dry blotter (Hoeffer, Canada) at 0.8 mA/cm^2 ^of membrane. Membranes were incubated in blocking buffer (phosphate buffer saline (Invitrogen, CA) and 0.1% TWEEN 20 (Sigma-Aldrich)) for 2 hours. Primary antibodies raised against MAO-A (1:300), TGM2 (1:400), HADHA (1:500), from Santa Cruz Biotechnology, CA, USA and GAPDH (1: 330000) from Sigma) were used. After thorough washing in the blocking buffer, secondary horseradish peroxidase-conjugated antibody (1:10 000, Santa Cruz Biotechnology) was added to membrane for one hour. Signal was detected using Western Blotting Luminol Reagent (Santa Cruz Biotechnology).

### mRNA expression analysis

Samples of LV tissue (n = 6 in each group) were immediately harvested into RNA preserving solution (RNA-Later, Ambiogen, USA). Total RNA was isolated (RNeasy-MicroKit, Qiagen, USA), checked for integrity, amplified, and hybridized on an Illumina RatRef-12v1 Expression BeadChip (Illumina, USA). The raw data were analyzed and processed using the beadarray package of the Bioconductor, as previously described [14]. Analysis of differential expression was performed with the Limmapackage [15] and annotated against the RatRef_12_V1_0_ R3_11222119_A.bgx maniphest (Illumina, USA). The cut-off level for differential regulation was set to the fold change [1.5 or \0.67 with Storey q\0.05]. The data are MIAME-compliant and are deposited in the ArrayExpress database (accession #: E-MTAB-190).

## Results and Discussion

We prepared cohorts of rats with an aorto-caval fistula (ACF) and sham-operated control animals. We determined functional and morphological changes in the failing ACF myocardium and performed differential proteomic and mRNA expression analysis of control and failing ACF myocardium.

### Cardiac morphometry and function

**Figure 1:**
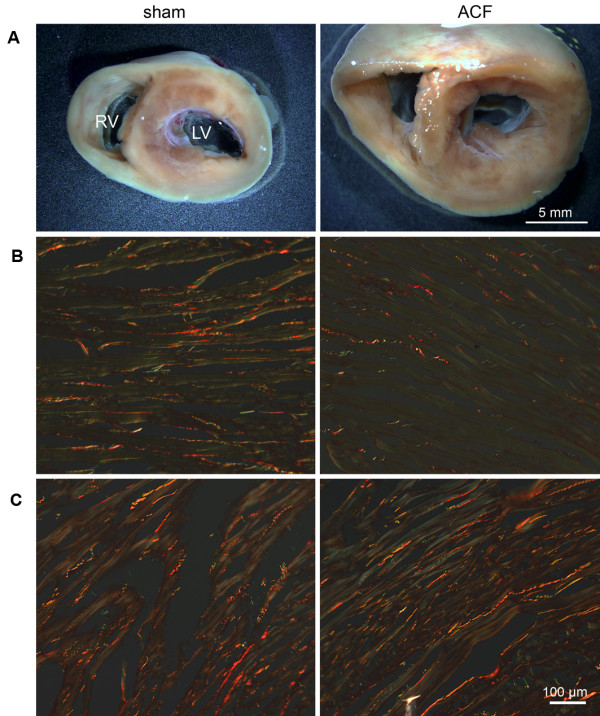
**Morphology of sham and ACF rat hearts 150 day after ACF creation**. **A**) Transversal section of the heart illustrates marked biventricular enlargement in the ACF animal compared to control sham-operated rats. **B,C**) Picrosirius Red staining in transverse sections, observed in polarized light, detected sparse mature collagen fibers (in red) well aligned with the myocyte bundles in the circular layer of the left ventricle (**B**). The amount of collagen was consistently higher in the right ventricle (**C**), but no difference between sham and ACF hearts was apparent. Green staining is due to erythrocytes, contractile proteins, or immature collagen fibrils. Very little green was observed at the edges of the collagen fibers, representing physiological protein turnover rather than tissue remodeling, with no difference between sham and ACF.

Rats with ACF had reached a similar body size as sham-operated controls and most of ACF animals showed clinical signs of HF such as piloerection, lethargy and difficult breathing 150 days after ACF creation. Compared to controls, ACF animals had markedly increased heart size (Figure 1A) and weights (5.29 ± 0.18 vs. 2.80 ± 0.12 g/100 g of body weight, p < 0.05) and increased lung weights indicating pulmonary congestion (Table 1). Echocardiography confirmed the enlargement of both ventricles and reduced fractional shortening of the left ventricle. These observations are compatible with incipient contractile dysfunction in ACF. Invasive hemodynamics showed increased end-diastolic LV (left ventricle) pressure also indicative of decompensated HF (Table 1). There was no marked fibrosis observed in ACF hearts (Figure 1B, C), in agreement with a previous report [16].

**Table 1:** Morphometric, hemodynamic and echocardiographic characteristics of cardiac function 150 days after ACF.

	Sham	ACF
***Morphometry***		
Body weight, *g*	592 ± 20.9	586 ± 23.4
Heart weight/BW, *g/100 g*	2.80 ± 0.12	5.29 ± 0.18 *
Lung Weight/BW, *g/100 g*	3.30 ± 0.16	4.23 ± 0.19 *
***Hemodynamics***		
Heart rate, *s^-1^*	344.9 ± 13.3	360.1 ± 10.8
Peak LV pressure, *mmHg*	129 ± 7.11	120 ± 3.96
End-diastolic LV pressure, *mmHg*	6.7 ± 0.84	12.1 ± 0.66 *
***Echocardiography***		
LV diastolic diameter, *mm*	6.08 ± 0.40	10.20 ± 0.48 *
LV systolic diameter, *mm*	1.95 ± 0.42	5.47 ± 0.42 *
LV Fractional shortening, *%*	69.2 ± 5.00	46.7 ± 2.46 *
LV anterior wall diastolic thickness, *mm*	2.30 ± 0.08	2.33 ± 0.09
LV posterior wall diastolic thickness, *mm*	2.33 ± 0.07	2.31 ± 0.09
RV diastolic diameter, *mm*	2.85 ± 0.18	5.07 ± 0.29 *

n = 10/group. Data are mean ± SE.

BW: Body weight, ACF: aorto-caval fistula group, LV: left ventricle, RV: right ventricle.

* significantly different (p < 0.05) than sham-operated animals.

### Proteomic and transcriptomic analysis

Six male rats with ACF and six sham-operated animals were included into our proteomic analysis and processed in two sub-pooled samples per group. Three independent labeling and separation experiments A, B and C were performed, resulting in a total 168 LC-runs, collecting over 110,000 MS/MS spectra. Mass spectrometry data from all three parallel analyses were merged and processed as a single dataset by Protein Pilot software. At high confidence (unused protein score 2.0 and confidence 99%) we identified 2030 individual proteins. For the expression analysis we considered only those proteins that were identified with at least two peptides, each peptide with at least 95% confidence. That reduced the number of identified proteins to 1446 with a false discovery rate (FDR) of only 0.48%. Based on the proteomic analysis (table 2), sixty six proteins were differentially expressed (p value<0.05, average iTRAQ ratio>1.5)

**Table 2:** Proteins differentially expressed in hearts of ACF rats.

Proteins downregulated in ACF					
**Peptides (95% confidence)**	**Seq. Cov**.	**Accession **	**Protein name**	**Protein Fold-change (iTRAQ ratio)**	**mRNA fold-change**

53	54	gi|259435950	Long-chain-fatty-acid-CoA ligase 1	0.23	NA
17	25	gi|59797483	Carnitine O-acetyltransferase	0.24	0.52
124	66.1	gi|189083744	Sarcomeric mitochondrial creatine kinase	0.24	1.05
42	63	gi|54035288	Enolase 3, beta	0.26	0.23
26	52	gi|57333	3-2 trans-enoyl-CoA isomerase	0.27	0.54
40	49	gi|60688124	Trifunctional enzyme subunit alpha, mitochondrial (HADHA)	0.3	0.54
24	35	gi|31077132	Histidine rich calcium binding protein	0.31	0.61
9	37	gi|1906812	Inducible carbonyl reductase	0.32	0.45
49	65	gi|56541110	Acyl-Coenzyme A dehydrogenase, very long chain	0.33	0.59
28	45	gi|510110	Trifunctional enzyme subunit beta, mitochondrial (HADHB)	0.33	0.56
4	17	gi|66910891	Glutamic-pyruvate transaminase (alanine aminotransferase)	0.34	0.38
113	53	gi|57303	Sarcoplasmic reticulum 2+-Ca-ATPase (SERCA2)	0.35	1.0
40	56.8	gi|149042663	Sarcalumenin	0.36	0.91
20	41.1	gi|77993368	Acyl-CoA synthetase family member 2 precursor	0.39	NA
120	74.3	gi|6978661	Muscle creatine kinase	0.4	0.69
195	75.8	gi|83300587	ATP synthase subunit alpha, mitochondrial;	0.4	0.71
120	71	gi|62079055	Isocitrate dehydrogenase 2 (NADP+)	0.41	0.62
30	50	gi|7387725	Medium and short chain L-3-hydroxyacyl-coenzyme A dehydrogenase	0.43	0.37
18	47.5	gi|51260066	Propionyl coenzyme A carboxylase, beta polypeptide	0.43	0.84
19	39	gi|6166586	Acyl-coenzyme A thioesterase 2	0.44	0.54
24	42.6	gi|149050263	Propionyl-CoA carboxylase alpha chain	0.44	0.91
35	40.7	gi|6978543	Na+/K+ -ATPase alpha 1 subunit precursor	0.45	1.1
34	64	gi|56929	Pyruvate kinase M1/M2	0.46	0.6
16	37	gi|62825891	Phosphofructokinase, muscle	0.46	0.5
42	68.8	gi|57527204	Electron-transfer-flavoprotein, alpha polypeptide	0.46	0.69
10	30	gi|149062241	LRP16 protein	0.47	0.38
35	47.9	gi|92090591	Glutamate dehydrogenase 1	0.47	0.84
13	43	gi|6981396	Protein kinase, cAMP dependent regulatory, type I, alpha	0.47	1.0
68	37	gi|61557127	Nicotinamide nucleotide transhydrogenase	0.48	0.67
111	69.1	gi|6978431	Long-chain acyl-CoA dehydrogenase precursor	0.49	0.84
31	49	gi|48734846	Acyl-Coenzyme A dehydrogenase, C-2 to C-3 short chain	0.53	0.58
64	44.5	gi|81883712	2-oxoglutarate dehydrogenase E1 component	0.53	0.69
48	67	gi|149027156	Acetyl-Coenzyme A acyltransferase 2	0.54	0.61
45	25.9	gi|189181710	Ryanodine receptor 2, cardiac	0.58	0.79
30	37	gi|81871846	Leucine-rich PPR motif-containing protein, mitochondrial	0.61	0.66
33	45	gi|6978705	Carnitine O-palmitoyltransferase precursor	0.61	0.58
					
**Proteins upregulated in ACF**					

**Peptides (95% confidence)**	**Seq. Cov**.	**Accession **	**Protein name**	**Protein Fold-change (iTRAQ ratio)**	**mRNA fold-change**

44	55	gi|48425083	Monoamine Oxidase A	4.06	1.93
10	18	gi|55249666	Cadherin 13	3.40	2.15
19	34	gi|5326787	Transglutaminase 2	3.07	1.93
24	61	gi|94400790	Heat shock protein 1 (HSP27)	3.05	1.41
23	72.2	gi|438878	tropomyosin	3.04	1.32
10	42	gi|6978501	Annexin A1	3.00	2.23
35	69.6	gi|535069	Muscle LIM protein [Rattus norvegicus]	2.97	1.31
22	50	gi|6981324	Prolyl 4-hydroxylase, beta polypeptide	2.91	1.27
59	73.3	gi|56388799	Brain creatine kinase (Ckb protein)	2.88	1.31
16	30	gi|149048530	Ceruloplasmin, isoform CRA_a	2.80	2.02
34	62.3	gi|744592	Alpha-B crystallin	2.61	1.05
35	68	gi|157830232	Annexin V	2.58	1.71
20	27.3	gi|462569	Microtubule-associated protein 1A	2.58	1.30
10	26.9	gi|158706096	Pre-B-cell leukemia transcription factor-interacting protein 1	2.45	1.23
8	34.4	gi|68837285	D-beta-hydroxybutyrate dehydrogenase, mitochondrial;	2.44	1.02
10	28	gi|974168	Aldehyde dehydrogenase 1A1 (retinal dehydrogenase 1)	2.43	1.84
11	28	gi|7533042	Guanine deaminase	2.41	2.02
8	28.9	gi|57241	Sulfated glycoprotein 2 (clusterin)	2.39	1.34
24	38.6	gi|6981022	Hexokinase 1	2.23	NA
59	64.4	gi|109468300	Alpha-enolase (Enolase 1)	2.23	1.00
94	50.7	gi|149063941	Beta myosin heavy chain myo7	2.22	1.02
14	35.3	gi|53237076	EH-domain containing 4	2.22	1.08
22	50	gi|9845234	Annexin A2	2.21	2.17
11	25	gi|149018456	Microtubule-associated protein 4	2.18	1.24
6	26	gi|158186676	Calumenin isoform a	2.17	0.86
39	42.6	gi|54673763	Heat shock protein 90, alpha (cytosolic), class A member 1	2.14	1.27
13	60.8	gi|1051270	14-3-3 zeta isoform	1.99	1.18
11	33.2	gi|55855	Calreticulin	1.90	1.18
41	26.9	gi|62646949	Filamin-C (Gamma-filamin) (Filamin-2)	1.87	1.21
18	39.1	gi|157819677	Sarcolemma associated protein	1.81	1.02

Identification of all proteins was based on at least four peptides. (for peptide sequences see Additional data 2). NA- mRNA not represented on the chip.

Transcriptomic analysis was performed using Illumina chips containing 23,401 rat genes. 16,206 transcripts were tested for differential expression, with 851 being differentially expressed (q-value<0.05). Complete mRNA expression data are deposited in the ArrayExpress database (accession #: E-MTAB-190).

Table 2 lists the 66 differentially expressed proteins, along with their respective mRNA expression data. Twenty nine of these proteins were differentially expressed with at least a 1.5-fold change at the mRNA level. Eighteen mRNAs showed less pronounced differential expression but with a trend corresponding with the respective proteins (i.e. up- or down-regulation). Three proteins were not represented on the array, and the expression of 16 mRNAs out of 66 was not altered.

The list of these 66 differentially expressed proteins including complete iTRAQ and mRNA statistics is available as Additional file 1, examples of 3 peptides used for their identification are as Additional file 2. All other proteins identified in our proteomic analysis are listed in Additional file 3.

**Figure 2:**
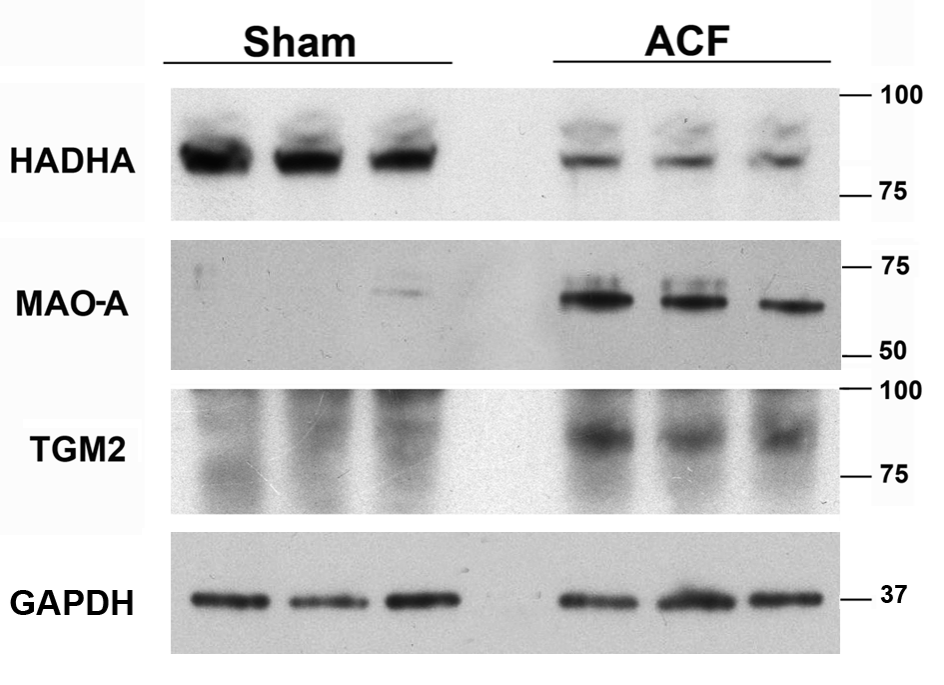
**Western blotting confirmation of the expression changes**. HADHA (trifunctional protein subunit A (Hydroxyacyl-Coenzyme A dehydrogenase/3-ketoacyl-Coenzyme A thiolase/enoyl-Coenzyme A hydratase alpha subunit), MAO-A (monoamine oxidase A), TGM2 (transglutaminase 2). Ten micrograms of protein was loaded per lane. GAPDH (Glyceraldehyde 3-phosphate dehydrogenase) was used as a loading control.

We further verified our results by western-blotting analysis of three proteins with potential therapeutic relevance - monoamine-oxidase A (MAO-A), transglutaminase 2 (TGM2) and a key protein of fatty acid beta oxidation - the alpha-subunit of mitochondrial trifunctional enzyme (HADHA) (Figure 2). The results confirm the upregulation of MAO-A and TGM2 and down-regulation of HADHA identified by proteomics and transcriptomics.

#### Molecular changes in the failing myocardium

Although contractile function of the heart appears to remain relatively preserved at this stage of HF, our proteomic analysis confirmed characteristic molecular features of HF such as profound changes in heart energetics and metabolism - namely the switch of substrate preference from fatty acids to other substrates are the hallmark of HF [17,18]. The largest group of differentially regulated proteins in ACF, representing approximately half of the differentially expressed proteins, is associated with energetic substrate metabolism (Table 2). We note the few cases where mRNA expression did not mimic protein changes, or was not present on micro array chips.

### Enzymes of fatty acid oxidation and electron transport chain

The most obvious, but not unexpected, alteration in the failing myocardium was the down-regulation of most key proteins involved in the β-oxidation of fatty acids (FA). The depressed expression or activity of individual enzymes involved in FA oxidation has been previously demonstrated in advanced HF patients and in most HF models [reviewed in [17] and [18]]. Attenuated myocardial oxidation of palmitate has recently been demonstrated in the same HF model by our group [19]. However, we note that net lipid oxidation can be increased in diabetic cardiac hypertrophy models [20]

We found *carnitine O-palmitoyltransferase 2*, responsible for the transport of FA across the inner mitochondrial membrane, to be downregulated. The key protein of beta oxidation the *mitochondrial trifunctional protein *was downregulated (both HADHA and HADHB subunits) as was *Acyl-CoA dehydrogenase (3 forms with different FA chain length specificity)*. Additionally, *3-2trans-enoyl-CoA isomerase *that is responsible for the catabolism of unsaturated FA and *Acyl-CoA thioesterase *(mitochondrial thioesterase, MTE-1), an enzyme responsible for the intra-mitochondrial generation of free FA anions from acyl-CoAs were both down-regulated in ACF. ACF animals in this study also showed significant downregulation of long-chain *acyl-CoA synthetase 1 *and *acyl-CoA synthetase family member 2 precursor *responsible for the initial binding of fatty acids to the coenzyme A moiety, however, their mRNAs were not represented on the Illumina chip.

### Glycolysis

Despite the existence of functional studies suggesting that a failing heart preferentially utilizes glucose [17], we found no convincing evidence of up-regulation of the glycolytic pathway in ACF. The key regulatory enzyme and the last enzyme of glycolysis - *phosphofructokinase *and *pyruvate kinase*, were both down-regulated in ACF. Failing hearts also showed decreased expression of muscle-specific *enolase-3 *(β form) but an increased abundance of the ubiquitous *enolase-1 *(no change at the mRNA level).

### Creatine kinase system

We observed decreased abundance of *sarcomeric mitochondrial (sMt-CK, no change observed at the mRNA level) *and *muscle (M-CK) creatine kinase *along with up-regulation of the *B-CK isoform *(1.3-fold up-regulation of mRNA) changes typical for animal and human HF [21]. Expression of the three corresponding mRNAs is in agreement with these trends. The CK system serves as a temporal buffer of high-energy phosphates (sMt-CK), and participates in an spatial enzymatic network (M-CK) responsible for the fast transport of high-energy phosphates from mitochondria to the contractile machinery [22,23]. Decreased CK levels may contribute to the diminished ATP flux via CK observed in HF [23] and contribute to the limited cardiac functional reserve.

### Sarcomeric and Calcium handling and proteins

Of the sarcomeric proteins, we observed up-regulation of the *β-myosin heavy chain *(myosin 7, (no change observed at mRNA level). The switch of the predominant myosin heavy chain from the α- to β-isoform affects the contractile phenotype, and is considered a hallmark of myocardial HF-induced remodeling [3]. The failing myocardium is also characterized by the diminished expression of proteins responsible for sarcoplasmic reticulum (SR) Ca^2+ ^uptake, handling and release [24]. Correspondingly, we observed marked down-regulation of *SR calcium ATPase *(SERCA2) protein (no change observed at the mRNA level) and of the main SR Ca^2+ ^release channel - *Ryanodine Receptor 2 *protein (RYR2) (0.79 fold down-regulation of mRNA) thus confirming the molecular HF phenotype in ACF rats. Down-regulation of both proteins in HF has been described previously and was implicated in diminished contractility, reduced SR Ca^2+ ^stores and less efficient energy utilization of Ca^2+ ^handling [25,26].

#### Redox state and stress-response related proteins

Abnormalities in the intracellular redox state have been implicated in most processes affecting cardiac function and the development of HF [27]. The antioxidant potential of the cell is determined by the content of reduced and oxidized glutathione (GSH and GSSG). A sufficiently high GSH/GSSG ratio is maintained by NADPH-dependent glutathione reductase. In cardiomyocytes, NADPH is produced by nicotinamide nucleotide transhydrogenase, isocitrate dehydrogenase, and the pentosephosphate pathway. Interestingly, mitochondrial *nicotinamide nucleotide transhydrogenase *(NNT), which accounts for up to 45% of the total NADPH supply [28], was markedly down-regulated in ACF animals. In addition, *NADP-dependent isocitrate dehydrogenase *(IDH2*) *which may further contribute to NADPH levels, was also down-regulated. Our observations led us to hypothesize that decreased expression of the two important NADPH producers could compromise the function of glutathione reductase, explaining the attenuated redox reserve. We recently demonstrated decreased GSH/GSSG ratio in the failing ACF myocardium [19], providing support to this idea.

#### Other proteins

Three members of the large annexin family: *Annexin A5*, *A2 *and *A1 were up-regulated*. Annexins are ubiquitous proteins associated with the inner cytoplasmatic membrane that are known to bind phospholipids in a Ca^2+^-dependent manner and to participate in a variety of membrane-related events [29] as well as in apoptosis, inflammation and coagulation. The role of annexins in cardiac physiology remains largely unknown. Interestingly, all three annexins (A1, A2 and A5) that were up-regulated in ACF hearts in our study have been previously implicated in calcium-dependent "cell membrane resealing". Such resealing may be relevant in hemodynamically-overloaded hearts with increased mechanical stress [[30] and references within]. *Annexin A5 *(expressed mostly in cardiomyocytes) and *annexin A2 *(detected only in endothelial cells and the extracellular matrix) but not *annexin A1 *have been previously reported to be up-regulated in hypertrophic and failing hearts [29]. Increased serum levels of *annexin A5 *has been demonstrated in a large study on heart disease patients and was considered to be a potential marker for hypertension-related HF [31]. However, authors of the study however failed to detect increased annexin A5 mRNA in the hearts, raising a question about the source of the protein. Here we demonstrate up-regulation of A1, A2 and A5 transcripts, which further supports these annexin proteins as potential HF markers.

The ACF myocardium displayed up-regulation of *Cadherin 13 *(T- cadherin). Until recently, its heart-related function has been unknown. This GPI-anchored, lipid-raft associated member of the cadherin superfamily recruits adiponectin to heart, and is critical for adiponectin-mediated cardioprotection [32]. Increased expression here can be explained as an adaptive compensation for altered levels of circulating adiponectin [33].

#### Potential therapeutic targets

Among the most markedly up-regulated proteins in our study are *transglutaminase 2 *and *monoamine oxidase A*. Since both proteins carry promising therapeutic potential we discuss them in detail.

*Transglutaminase 2 *(TGM2) *was *up-regulated 3-fold in ACF hearts. TGM2 is a multifunctional protein with G-protein function, disulfide-isomerase and transglutaminase activities, found predominantly in the cytosol and at the cell curface. The transglutaminase activity of this protein is responsible for stable cross-linking of peptide chains between lysine and glutamine residues involved in extracellular matrix stabilization and wound healing as well as during apoptosis [34,35]. Due to its G-protein properties, TGM2 participates in intracellular signaling via α1-adrenergic and thromboxane receptors [34], and has been recently shown to promote apoptosis of rat cardiomyocytes under oxidative stress [36]. Two independent groups have demonstrated that heart-specific TGM2 over-expression results in detrimental hemodynamic changes, structural alterations, cardiomyocyte apoptosis, cardiac hypertrophy and fibrosis [37,38]. Our observation of up-regulated TGM2 in ACF hearts thus adds further evidence for the adverse effect of TGM2 up-regulation in cardiac hypertrophy and HF. Effective low molecular weight inhibitors such as cystamine and monodansylcadaverine are already known and their use inhibits TGM2-induced apoptosis in aortic smooth muscle cells [39] and partially repressed hypoxia-induced cardiac hypertrophy in rats [40]. This highlights the potential of TGM2 as a novel therapeutic target.

### Monoamine oxidase A

The most up-regulated (4-fold) protein in ACF heart is mitochondrial *monoamine oxidase-A *(MAO-A), an enzyme responsible for oxidative deamination of bioactive monoamines (epinephrine, norepinephrine, serotonin), giving rise to hydrogen peroxide and toxic aldehyde metabolites that are further catabolized by aldehyde dehydrogenases [41]. In concordance with this, *aldehyde dehydrogenase 1A1 *was also found to be up-regulated in ACF hearts in our study. The hydrogen peroxide produced by cardiac MAO-A has been shown to contribute to cardiomyocyte apoptosis [42]. Kaludercic et al. recently demonstrated that increased MAO-A-dependent catabolism of norepinephrine contributes to adverse remodeling in pressure-overloaded hearts. Pharmacological inhibition of MAO-A by clorgyline prevents left ventricle dilatation and dysfunction, attenuated oxidative stress and increased norepinephrine myocardial content in pressure overloaded hearts [43]. In an identical model to ours, Kristen et al. showed that ACF rats have increased circulating norepinephrine levels, but depleted cardiac norepinephrine stores [44]. In combination with the studies discussed above, our findings suggest that besides the loss of sympathetic nerve endings [44] or attenuated norepinephrine reuptake [45], myocardial norepinephrine depletion in HF may result from its increased catabolism by MAO-A. This process is common to both pressure and volume overload, and along with tissue norepinephrine depletion causes oxidative damage to cardiomyocytes. Interestingly, MAO-A has also been recently identified as a causal agent of oxidative myofibril damage in muscular dystrophy [46]. All experimental evidence summarized in a recent review [47] along with our observations strongly indicates that MAO-A expression/activity is a major contributor to cardiac hypertrophy and HF. Low-molecular weight inhibitors of MAO-A such as moclobemide exist and are already in clinical use as antidepressants [48]. Therefore, targeted inhibition of MAO-A activity should be intensively investigated as a potential therapy for HF.

#### Proteins with no previous association with HF

Of the 66 differentially expressed proteins at least 6 molecules have not been previously associated with heart HF and might therefore be new players in the disease development or progression. No previous connection with HF has been made for *inducible carbonyl reductase*, *LRP16 *(a compound of the NF-κB transcriptional complex) [49] or *Leucine-rich PPR motif-containing protein *(a regulator of mitochondrial transcription) [50] all down-regulated in ACF. These molecules seem to be involved in metabolic and regulatory processes, but information available on these three molecules is very limited. The up-regulated regulatory protein *Pre-B-cell leukemia transcription factor-interacting protein *alias HPIP (1.3 -fold up-regulation of mRNA) has been previously studied in the context of MAPK and AKT activation and estrogen receptor (ERα) and tubulin binding [51], but no connection with heart has been made to date. The up-regulated proteins *guanine deaminase *and *ceruloplasmin *although well known, also have yet to be connected with HF. Ceruloplasmin is a copper binding protein with ferroxidase activity, its altered expression thus may point out toward altered copper or iron homeostasis in HF. Notably copper metabolism or balance appears to be disrupted in diabetic hypertrophied hearts, and copper chelation has been shown to improve heart diabetic cardiac function [52]. The individual roles of these potential new players in the molecular puzzle of HF remain to be determined in future targeted studies.

## Conclusions

To our knowledge, our shot-gun study employing peptide IEF combined with nanoLC-MALDI is the largest (over 2000 proteins) semi-quantitative analysis of proteome changes related to HF to date. We are aware that our experimental design using two sub-pooled controls and two ACF sub-pools is not typical. This design was driven by our aim to penetrate deeper into medium- and low-abundance proteome and maximize the number of reliably identified and quantified proteins. Merging of MS data from three biologically identical runs provided us with a higher number of identified proteins with higher sequence coverage, and simultaneously increased the number of observed iTRAQ reporter quartets for each protein, thus increasing the reliability of the quantitative information. Our second reason for using this approach is economic. A higher statistical power for the experiment could have been achieved with iTRAQ quadruplex by analysis of one control pool against three individual ACF animals (or three ACF subpools). However, such a single control (sham-operated animals) pool would have to consist of many animals to eliminate the risk of a single atypical rat affecting the composition of such a representative control pool. Unfortunately, to operate and maintain large cohorts of such animals for almost half a year is economically prohibitive.

Various proteomics strategies have provided several important "snapshots" of different stages and types of heart hypertrophy and HF resulting from diverse initial insults, different underlying molecular mechanisms, and in different animal models. In this respect the results of different proteomic analyses are difficult to compare. However, the similarity of our results with the work of Grant et al. [53], who used an analogical proteomic approach to examine effect the of aging on the cardiac proteome in old versus young rats, is very intriguing. Similar to our results, aged hearts showed the down-regulation of enzymes of fatty acids oxidation, SMt- and M- creatine kinase, electron-transferring flavoprotein and ATP synthase components. Also in agreement with our study, aged hearts displayed up-regulated β-myosin heavy chain, muscle LIM protein, microtubule associated proteins 1 and 4, calumenin, calreticulin, annexin 5, prolyl-4-hydroxylase beta subunit, HSP 27 and alpha-B crystallin. Based on the high concordance of proteomic alterations induced by spontaneous aging and by overload-induced HF, it is tempting, however speculative, to view the HF developed in our model as a sort of accelerated, premature aging of the organ.

We are fully aware that our study has one significant limitation. Being based on a pair-wise comparison our study lacks important temporal information and can not discriminate between processes of compensatory hypertrophy and later events of HF itself. To access such a temporal information on the development process and gradual progression of HF, more time points will have to be analyzed in the future.

In summary, we identified multiple enzymes involved in substrate metabolism in the HF myocardium. This confirms many previous observations and is in accordance with altered substrate preference in the HF [17,18]. These alterations probably reflect the activation of a pro-survival program of stressed cells, and at least some changes may be adaptive, maximizing cardiac efficiency. Our study brings a novel observation suggesting an attenuated redox reserve (down-regulation of NADPH producers) in ACF rats which possibly contributes to the myocardial remodeling in HF due to oxidative stress. Further, we propose new potential biomarkers of hypertrophy and/or HF (annexin A2 and A1) and, most importantly, suggest two highly potential therapeutic targets for the treatment of HF - monoamine oxidase A and transglutaminase 2. Our work has also identified several proteins, new in the context of HF, as leads for specific, hypothesis-driven experiments.

## Abbreviations

HF: Heart Failure; ACF: Aorto-caval fistula; LV: Left ventricle; FA: fatty acids; TGG: transglutaminase 2; MAO-A: monoamine oxidase A; HADHA: Hydroxyacyl-Coenzyme A dehydrogenase/3-ketoacyl-Coenzyme A thiolase/enoyl-Coenzyme A hydratase alpha subunit; SERCA2: sarcoplasic reticulum ^2+^Ca ATPase.

## Competing interests

The authors declare that they have no competing interests.

## Authors' contributions

JP and VM designed the study, interpreted the data and wrote the manuscript. JaP, LL and OV performed the sample preparation, peptide labeling and separation, and western blotting experiments. MS and PJ performed the LC-MS analysis. VM, JB, LC and DS prepared the ACF animals, measured the hemodynamic and echocardiographic paramaters and performed the morphological analysis. MK and HS were responsible for the mRNA chip analysis. All authors read and approved the final manuscript.

## Supplementary Material

Additional file 1 **Additional data 1_ statistics of differentially expressed proteins and mRNAs.pdf**. Table presents statistical significance data on the differential expression of individual proteins (iTRAQ ratios) and their respective mRNA expression. Click here for file

Additional file 2 **Additional data 3_peptides used for protein identifications .pdf**. Table shows sequences of three of the *n *peptides used for the identification of the 66 differentially expressed proteins. Click here for file

Additional file 3 **Additional data 3_ all identified proteins.pdf**. Extensive table summarizes all other proteins (not differentially expressed) identified by MS including their accession numbers, sequence coverage and number of peptides observed. Click here for file
