# Supplementary material for: Proteomic and transcriptomic analysis of heart failure due to volume overload in a rat aorto-caval fistula model provides support for new potential therapeutic targets - monoamine oxidase A and transglutaminase 2
Source: Proteome Sci. 2011 Nov 11;9:69. doi: 10.1186/1477-5956-9-69 (PMC3225319; doi:10.1186/1477-5956-9-69)
Supplement: Additional file 1 — Additional data 1_ statistics of differentially expressed proteins and mRNAs.pdf. Table presents statistical significance data on the differential expression of individual proteins (iTRAQ ratios) and their respective mRNA expression. [file 1477-5956-9-69-S1.PDF]

Proteins downregulated in ACF

Peptides (95% confidence)	Seq. Cov.	Accession	Protein name	Protein Fold-change (iTRAQ ratio)	PVal 116:114	PVal 117:114	PVal 116:115	PVal 117:115	mRNA fold-change	mRNA adjusted p-value
53	54	gi|259435950	Long-chain-fatty-acid-CoA ligase 1	0.23	0.0095	<0,0001	0.0005	<0,0001	NA	NA
17	25	gi|59797483	Carnitine O-acetyltransferase	0.24	0.0058	0.0046	0.007	0.0055	0.52	0.00140
124	66.1	gi|189083744	sarcomeric mitochondrial creatine kinase precursor	0.24	<0,0001	<0,0001	<0,0001	0.0001	1.05	0.550742
42	63	gi|54035288	Enolase 3, beta	0.26	0.0459	0.0244	0.0037	0.0031	0.23	0.00003
26	52	gi|57333	3-2 trans-enoyl-CoA isomerase	0.27	0.0213	0.0029	0.0141	0.0042	0.54	0.00044
40	49	gi|60688124	Trifunctional enzyme subunit alpha, mitochondrial (HADHA)	0.3	0.0082	0.0055	0.0028	0.002	0.54	0.00063
24	35	gi|31077132	Histidine rich calcium binding protein	0.31	0.0092	0.001	0.0011	0.0007	0.61	0.00153
9	37	gi|1906812	Inducible carbonyl reductase	0.32	0.019	0.0377	0.013	0.0238	0.45	0.00899
49	65	gi|56541110	Acyl-Coenzyme A dehydrogenase, very long chain	0.33	0.0001	<0,0001	0.0049	0.0004	0.59	0.00046
28	45	gi|510110	Trifunctional enzyme subunit beta, mitochondrial (HADHB)	0.33	0.005	0.0012	0.0125	0.0026	0.56	0.00005
4	17	gi|66910891	Glutamic-pyruvate transaminase (alanine aminotransferase)	0.34	0.001	0.0031	0.0035	0.0378	0.38	0.00002
113	53	gi|57303	Sarcoplasmic reticulum 2+-Ca-ATPase (SERCA2)	0.35	0.0128	0.0357	0.001	0.0018	1	0.104355
40	56.8	gi|149042663	sarcalumenin (predicted), isoform CRA_a	0.36	0.0036	0.0286	0.0029	0.0093	0.91	0.528607
20	41.1	gi|77993368	acyl-CoA synthetase family member 2 precursor	0.39	0.004	0.0004	0.0489	0.0041	NA	NA
120	74.3	gi|6978661	muscle creatine kinase	0.4	0.0192	0.0009	0.0291	0.0006	0.69	0.002701
195	75.8	gi|83300587	ATP synthase subunit alpha, mitochondrial;	0.4	0.0033	0.0305	0.0002	0.0023	0.71	0.0002
120	71	gi|62079055	Isocitrate dehydrogenase 2 (NADP+), mitochondrial precursor	0.41	0.0125	0.0267	0.0006	0.0012	0.62	0.00219
30	50	gi|7387725	Medium and short chain L-3-hydroxyacyl-coenzyme A dehydrogenase	0.43	0.0017	0.0029	0.0332	0.0872	0.37	0.00079
18	47.5	gi|51260066	Propionyl coenzyme A carboxylase, beta polypeptide	0.43	0.0277	0.0224	0.037	0.0206	0.84	0.035658
19	39	gi|6166586	Acyl-coenzyme A thioesterase 2, mitochondrial;	0.44	0.0295	0.0197	0.0422	0.0275	0.54	0.00366

24	42.6	gi|149050263	Propionyl-CoA carboxylase alpha chain rCG36968,	0.44	0.0144	0.0249	0.0195	0.0143	0.91	0.085376
35	40.7	gi|6978543	Na+/K+ -ATPase alpha 1 subunit precursor	0.45	0.0232	0.0198	0.0479	0.0407	1.01	0.824869
34	64	gi|56929	Pyruvate kinase M1/M2	0.46	<0,0001	<0,0001	0.0305	0.026	0.6	0.01110
16	37	gi|62825891	Phosphofructokinase, muscle	0.46	0.0066	0.045	0.032	0.046	0.5	0.00019
42	68.8	gi|57527204	electron-transfer-flavoprotein, alpha polypeptide precursor	0.46	0.0081	0.0353	0.0267	0.0338	0.69	0.00125
10	30	gi|149062241	LRP16 protein	0.47	0.0431	0.0222	0.0414	0.0214	0.38	0.00027
35	47.9	gi|92090591	Glutamate dehydrogenase 1, mitochondrial; Short=GDH;	0.47	0.0177	0.0039	0.03	0.0029	0.84	0.014172
13	43	gi|6981396	protein kinase, cAMP dependent regulatory, type I, alpha	0.47	0.0007	0.0017	0.0023	0.006	1	0.988789
68	37	gi|61557127	Nicotinamide nucleotide transhydrogenase	0.48	0.0001	<0,0001	0.014	<0,0001	0.67	0.00005
111	69.1	gi|6978431	long-chain acyl-CoA dehydrogenase precursor	0.49	0.0249	0.0385	0.031	0.0478	0.84	0.00888
31	49	gi|48734846	Acyl-Coenzyme A dehydrogenase, C-2 to C-3 short chain	0.53	0.022	0.0129	0.0072	0.0045	0.58	0.00009
64	44.5	gi|81883712	RecName: Full=2-oxoglutarate dehydrogenase E1 component	0.53	0.0319	0.0327	0.0035	0.0036	0.69	0.003779
48	67	gi|149027156	Acetyl-Coenzyme A acyltransferase 2	0.54	0.0065	0.0011	0.0162	0.0037	0.61	0.00635
45	25.9	gi|189181710	ryanodine receptor 2, cardiac	0.58	0.0001	0.0004	0.0102	0.0302	0.79	0.035924
30	37	gi|81871846	Leucine-rich PPR motif-containing protein, mitochondrial	0.61	0.0058	0.0022	0.0368	0.007	0.66	0.00175
33	45	gi|6978705	Carnitine O-palmitoyltransferase precursor	0.61	0.0233	0.0287	0.048	0.021	0.58	0.00008

Proteins upregulated in ACF

Peptides (95% confidence)	Seq. Cov.	Accession	Protein name	Protein Fold-change (iTRAQ ratio)	PVal 116:114	PVal 117:114	PVal 116:115	PVal 117:115	mRNA fold-change	mRNA adjusted p-value
44	55	gi|48425083	Monoamine Oxidase A	4.06	<0,0001	<0,0001	<0,0001	<0,0001	1.93	0.01332
10	18	gi|55249666	Cadherin 13	3.4	0.006	0.007	0.0371	0.0455	2.15	0.00008
19	34	gi|5326787	Transglutaminase 2	3.07	0.0004	0.0012	0.001	0.0035	1.93	0.00105
24	61	gi|94400790	Heat shock protein 1 (HSP27)	3.05	0.0495	0.048	0.0465	0.0498	1.41	0.0317983
23	72.2	gi|438878	tropomyosin [Rattus norvegicus]	3.04	0.0095	0.0135	0.0057	0.0079	1.32	0.0029994
10	42	gi|6978501	Annexin A1	3	0.0016	0.003	0.0017	0.0033	2.23	0.00003
35	69.6	gi|535069	muscle LIM protein [Rattus norvegicus]	2.97	0.0006	0.0013	0.0034	0.0075	1.31	0.0005121
22	50	gi|6981324	Prolyl 4-hydroxylase, beta polypeptide	2.91	0.0004	0.0037	0.0001	0.001	1.27	0.0103887
59	73.3	gi|56388799	Ckb protein [Rattus norvegicus]	2.88	0.0184	0.0072	0.0319	0.0118	1.31	0.0396429
16	30	gi|149048530	Ceruloplasmin, isoform CRA_a	2.8	0.0093	0.0146	0.0046	0.0071	2.02	0.00010
34	62.3	gi|744592	alpha-B crystallin	2.61	0.0479	0.0408	0.033	0.0284	1.05	0.4601382
20	27.3	gi|462569	Microtubule-associated protein 1A	2.58	0.0025	0.0027	0.0006	0.0006	1.3	0.0140246
35	68	gi|157830232	Annexin V	2.58	0.0001	<0,0001	0.0007	<0,0001	1.71	0.00018
10	26.9	gi|158706096	Pre-B-cell leukemia transcription factor-interacting protein 1	2.45	0.0093	0.0032	0.0184	0.0061	1.23	0.2822604
8	34.4	gi|68837285	D-beta-hydroxybutyrate dehydrogenase, mitochondrial;	2.44	0.0229	0.0206	0.0105	0.006	1.02	0.9286606
10	28	gi|974168	Aldehyde dehydrogenase 1A1 (retinal dehydrogenase 1)	2.43	0.0208	0.0074	0.0133	0.0049	1.84	0.00971
11	28	gi|7533042	Guanine deaminase	2.41	0.0172	0.02	0.0123	0.0164	2.02	0.00035

**8**	**28.9**	**gi|57241**	**sulfated glycoprotein 2 (clusterin) [Rattus norvegicus]**	2.39	0.0475	0.0076	0.0113	0.0022	1.34	0.0008996
**24**	**38.6**	**gi|6981022**	**hexokinase 1 [Rattus norvegicus]**	2.23	0.0001	<0,0001	0.0079	0.0018	NA	NA
**59**	**64.4**	**gi|109468300**	**Alpha-enolase (Non-neural enolase) (NNE) (Enolase 1)**	2.23	0.0293	0.0209	0.0397	0.0271	1	0.9887893
**94**	**50.7**	**gi|149063941**	**beta myosin heavy chain myo7, rCG23467, isoform CRA_a**	2.22	<0,0001	0.0082	0.0061	0.0049	1.02	0.8552522
**14**	**35.3**	**gi|53237076**	**EH-domain containing 4 [Rattus norvegicus]**	2.22	0.0145	0.0163	0.0149	0.0169	1.08	0.4254535
**22**	**50**	**gi|9845234**	**Annexin A2**	2.21	0.02	0.0068	0.0217	0.0074	2.17	0.00004
**11**	**25**	**gi|149018456**	**microtubule-associated protein 4 [Rattus norvegicus]**	2.18	0.0136	0.0028	0.0495	0.0094	1.24	0.0067132
**6**	**26**	**gi|158186676**	**calumenin isoform a [Rattus norvegicus]**	2.17	0.0066	0.0456	0.0053	0.0355	0.86	0.3567508
**39**	**42.6**	**gi|54673763**	**Heat shock protein 90, alpha (cytosolic), class A member 1**	2.14	0.0237	0.0083	0.0146	0.0053	1.27	0.0161013
**13**	**60.8**	**gi|1051270**	**14-3-3 zeta isoform [Rattus norvegicus]**	1.99	0.0064	0.0014	0.0154	0.004	1.18	0.0376797
**11**	**33.2**	**gi|55855**	**Calreticulin precursor (AA -17 to 399)**	1.9	0.0294	0.0179	0.0268	0.0163	1.18	0.108613
**41**	**26.9**	**gi|62646949**	**Filamin-C (Gamma-filamin) (Filamin-2) (Protein FLNc)**	1.87	0.0032	0.0001	0.0028	0.0001	1.21	0.173164
**18**	**39.1**	**gi|157819677**	**sarcolemma associated protein**	1.81	0.0008	0.001	0.008	0.0095	1.02	0.9257805
