# Supplementary material for: Proteomic and transcriptomic analysis of heart failure due to volume overload in a rat aorto-caval fistula model provides support for new potential therapeutic targets - monoamine oxidase A and transglutaminase 2
Source: Proteome Sci. 2011 Nov 11;9:69. doi: 10.1186/1477-5956-9-69 (PMC3225319; doi:10.1186/1477-5956-9-69)
Supplement: Additional file 2 — Additional data 3_peptides used for protein identifications .pdf. Table shows sequences of three of the n peptides used for the identification of the 66 differentially expressed proteins. [file 1477-5956-9-69-S2.PDF]

Differentially expressed proteins and 3 of the peptides used for identification

DOWNREGULATED

peptides	Seq. Cov	Protein name	Three of the peptides used for identification		
4	17	Glutamic-pyruvate transaminase (alanine aminotransferase)	AWALDIAELR	ECIEAVIR	EDVAQYIER
9	37	Inducible carbonyl reductase	FHQLDIDNPQSIR	FLGDVVLTAR	SETITEEELVGLMNK
10	30	LRP16 protein	AAGSLLTDECR	DEGIYQER	EEHYFCK
13	43	protein kinase, cAMP dependent regulatory, type I, alpha	ECELYVQK	GAISAEVYTEEDAASYVR	LTVADALEPVQFEDGQK
16	37	Phosphofructokinase, muscle	DLQVNVEHLVQK	DQTDFEHR	GITNLCVIGGDGSLTGADTFR
17	25	Carnitine O-acetyltransferase	ALQPIVSEEEWAHTK	FNITPEIK	GMDDPK
18	47.5	Propionyl coenzyme A carboxylase, beta polypeptide	AFDNDVDALCNLR	AYGGAYDVMSSK	CADFGMAAEK
19	39	Acyl-coenzyme A thioesterase 2, mitochondrial;	ADAGGELDLAR	DETIPPVSLLR	DVQKPYVVELEVLDGHEPDGGQR
20	41.1	acyl-CoA synthetase family member 2 precursor	EQMEQHLK	EQNLAQLR	GGENIYPAELEDFFHK
24	35	Histidine rich calcium binding protein	EEDEDFSR	EEEDGVVSGEDHR	ENQEEATVK
24	42.6	Propionyl-CoA carboxylase alpha chain rCG36968,	AGDTVGEGDLLVELE	CLAAEDVTFIGPDTHAIQAMGDK	EIGYPVMIK
26	52	3-2 trans-enoyl-CoA isomerase	ALQLGTLFPPAEALK	EADIQNFTSFISR	EGEAGIAVMK
28	45	Trifunctional enzyme subunit beta, mitochondrial (HADHB)	AMDSDWFAQNYMGR	DFIYVSQDPK	DLMPHDLAR
30	50	Medium and short chain L-3-hydroxyacyl-coenzyme A dehydrogenase	AADEFVEK	EDIDTAMK	GIEESLKR
30	37	Leucine-rich PPR motif-containing protein, mitochondrial	AALDLEQVPSELAVTR	AEGHPVGDPLK	AGYPQYVSEILEK
31	49	Acyl-Coenzyme A dehydrogenase, C-2 to C-3 short chain	ASSTANLIFEDCR	ELVPIAAQLDK	ENLLGEPGMGFK
33	45	Carnitine O-palmitoyltransferase precursor	DELFTDTK	EAAMGQGFDR	EKFDTTVK
34	64	Pyruvate kinase M1/M2	AATESFASDPILYR	ASSQSTDPLEAMAMGSVEASYK	CDENILWLDYK
35	40.7	Na+/K+ -ATPase alpha 1 subunit precursor	ADIGVAMGIVGSDVSK	AVAGDASESALLK	DAFQNAYLELGGLGER

peptides	Seq. Cov	Protein name	Three of the peptides used for identification		
35	47.9	Glutamate dehydrogenase 1, mitochondrial; Short=GDH;	CAVVDVPFGGAK	CVGVGESDGSIWNPDGIDPK	DDGSWEVIEGYR
40	49	Trifunctional enzyme subunit alpha, mitochondrial (HADHA)	CLAPMMSEVIR	DSIFSNLIGQLDYK	DTTASAVAVGLK
40	56.8	sarcalumenin (predicted), isoform CRA_a	ADNLATQMLMR	AEVDAETGEEAEDQGEPR	AITQELPSLLGSIGLGK
42	63	Enolase 3, beta	FMIELDGTENK	IAQAVEK	IEEALGDK
42	68.8	electron-transfer-flavoprotein, alpha polypeptide precursor	AAVDAGFVPNDMQVGQTGK	APSSSSAGISEWLDQK	GTSFEAAAASGGSASSEK
45	25.9	ryanodine receptor 2, cardiac	AAISDQER	AEDTSDPER	AEGEDGEKEEK
48	67	Acetyl-Coenzyme A acyltransferase 2	AANEAGYFNEEMAPIEVK	DAEVVLCGGTESMSQSPYSVR	DFTATDLTEFAAR
49	65	Acyl-Coenzyme A dehydrogenase, very long chain	ASNTSEVYFDGVK	DFQIEAAISK	ENMASLQSNPQQQELFR
53	54	Long-chain-fatty-acid-CoA ligase 1	AELSVIFADKPEK	AILEDMVK	ALEDLGR
64	44.5	RecName: Full=2-oxoglutarate dehydrogenase E1 component	AEQFYCGDTEGK	DMAEEVAITR	ELEQIFCQFDSK
68	37	Nicotinamide nucleotide transhydrogenase	AQYPIADLVK	DDFDFGTMSHVIR	DGNVIFPAPTPK
111	69.1	long-chain acyl-CoA dehydrogenase precursor	AFVDSCLQLHETK	AQDTAELFFEDVR	CIGAIAMTEPGAGSDLQGVR
113	53	Sarcoplasmic reticulum 2+-Ca-ATPase (SERCA2)	AKDIVPGDIVEIAVGDK	AMGVVVATGVNTEIGK	CLALATHDNPLR
120	71	Isocitrate dehydrogenase 2 (NADP+), mitochondrial precursor	ATDFVVDR	CATITPDEAR	DIFQEIFDK
120	74.3	muscle creatine kinase	DLFDPIIQDR	FEEILTR	GQSIDDMIPAQK
124	66.1	sarcomeric mitochondrial creatine kinase precursor	EVENVAITALEGLK	GTGGVDTAAVADVYDISNIDR	GWEFMWNER
195	75.8	RecName: Full=ATP synthase subunit alpha, mitochondrial;	AIEEQVAVIYAGVR	AVDSLVPIGR	EAYPGDVFYLHSR

UPREGULATED

peptides	Seq. Cov	Protein name	Three of the peptides used for identification		
6	26	calumenin isoform a [Rattus norvegicus]	EQFVEFR	HLVYESDQDKDGK	MDKEETK
8	28.9	sulfated glycoprotein 2 (clusterin) [Rattus norvegicus]	ASGIIDTLFQDR	FMDTVAEK	IDSLLESDR
8	34.4	D-beta-hydroxybutyrate dehydrogenase, mitochondrial;	EVAEVNLWGTVR	FGVEAFSDCLR	KMWDELPEVVR
10	18	Cadherin 13	DLHPNTDPFK	DLHPNTDPFK	ETIATYQLFVETTDASGR
10	42	Annexin A1	ALYEAGER	CEDMSVNQDLADTDAR	DITSDTSGDFR
10	28	Aldehyde dehydrogenase 1A1 (retinal dehydrogenase 1)	EEIFGPVQQIMK	ELGEHGLYEYTELK	IFVEESVYDEFVR
10	26.9	Pre-B-cell leukemia transcription factor-interacting protein 1	EPSPPQPTAAVDGEDQAK	GALGGDDGHGTK	GQEPDTSLLEQHK
11	28	Guanine deaminase	DFDALLINPR	DHLLGVSDSGK	EIGNFEVGK
11	33.2	Calreticulin precursor (AA -17 to 399)	DKQDEEQR	EQFLDGDAWTNR	FYGDQEK
11	25	microtubule-associated protein 4 [Rattus norvegicus]	AVDLESVK	DTMSSVEPDISSAK	DVSPSPETETAK
13	60.8	14-3-3 zeta isoform [Rattus norvegicus]	DNLTLWTSDTQGDEA EAGEGGEN	GIVDQSQQAYQEAFEISK	SVTEQGAELSNEER
14	35.3	EH-domain containing 4 [Rattus norvegicus]	ADQVDTQQLMR	AMQEQLENYDFTK	EGADEEEWVVAK
16	30	Ceruloplasmin, isoform CRA_a	ALYSEYTDGTFTK	ANEPSPGEGDSNCVTR	DCNKPSPDDDIQDR
18	39.1	sarcolemma associated protein	AASEYEEEIR	AELEGWR	DTDFMSLQEELK
19	34	Transglutaminase 2	DDREDITYTYK	DLYLENPEIK	EDITYTYK
20	27.3	Microtubule-associated protein 1A; Short=MAP-1A;	ADSVEQQDGAALEK	APEHSIPEPTQTDR	DTDLQQTQATEPR
22	50	Annexin A2	AEDGSVIDYELIDQDAR	DALNIETAIK	DIISDTSGEFR
22	50	Prolyl 4-hydroxylase, beta polypeptide	DHENIVIAK	EADDIVNWLK	ILFIFIDSDHTDNQR
23	72.2	tropomyosin [Rattus norvegicus]	ALKDEEK	CLSAAEEK	EQAEAEVASLNR
24	38.6	hexokinase 1 [Rattus norvegicus]	ASGVEGADVVK	ATDCEGHDVASLLR	CTVSFLLSEDGSGK
24	61	Heat shock protein 1 (HSP27)	AQIGGPESEQSGAK	AVTQSAEITIPVTFEAR	EGVVEITGK

peptides	Seq. Cov	Protein name	Three of the peptides used for identification		
34	62.3	alpha-B crystallin	APSWIDTGLSEMR	EEKPAVTAAPK	HFSPEELK
35	68	Annexin V	ADAEVLR	ALLLLCGGEDD	DLVNDMK
35	69.6	muscle LIM protein [Rattus norvegicus]	ALDSTTVAAHESEIYCK	NFGPTGIGFGGLTHQVEK	PNWGGGAK
39	42.6	Heat shock protein 90, alpha (cytosolic), class A member 1	APFDLFENR	DQVANSAFVER	ELISNSSDALDK
41	26.9	Filamin-C (Gamma-filamin) (Filamin-2) (Protein FLNc)	AEIAFEDR	APLQVAVLGPTGVAEPVEVR	DAGEGGLSLAVEGPSK
44	55	Monoamine Oxidase A	DIWVEEPESK	DVPAIEITHTFLER	EIPVDAPWQAR
59	64.4	Alpha-enolase (Non-neural enolase) (NNE) (Enolase 1)	DATNVGDEGGFAPNILENK	FTATAGIQVVGDDLTVTNPK	IEEELGSK
59	73.3	Ckb protein [Rattus norvegicus]	FCTGLTQIETLFK	GTGGVDTAAVGGVFDVSNADR	LAVEALSSLDGDLSGR
94	50.7	beta myosin heavy chain myo7, rCG23467, isoform CRA_a	DFELNALNAR	DVFVPDDKEEFVK	EQYEEETEAK
