# Supplementary material for: Proteomic and transcriptomic analysis of heart failure due to volume overload in a rat aorto-caval fistula model provides support for new potential therapeutic targets - monoamine oxidase A and transglutaminase 2
Source: Proteome Sci. 2011 Nov 11;9:69. doi: 10.1186/1477-5956-9-69 (PMC3225319; doi:10.1186/1477-5956-9-69)
Supplement: Additional file 3 — Additional data 3_ all identified proteins.pdf. Extensive table summarizes all other proteins (not differentially expressed) identified by MS including their accession numbers, sequence coverage and number of peptides observed. [file 1477-5956-9-69-S3.PDF]

# Proteins identified but not differentially expressed

| Accession # | Name | % Seq Cov | Peptides (95%) |
|---|---|---|---|
| gi\|37748456 | 2,4-dienoyl CoA reductase 1, mitochondrial [Rattus norvegicus] | 39,1 | 10 |
| gi\|3288594 | 26S proteasome subunit p112 [Rattus norvegicus] | 20,6 | 10 |
| gi\|1580888 | 2-oxoglutarate carrier protein | 53,2 | 14 |
| gi\|423755 | 3',5'-cyclic adenosine monophosphate-dependent protein kinase inhibitor alpha isoform - rat | 64,5 | 2 |
| gi\|157816997 | 3-hydroxybutyrate dehydrogenase, type 2 [Rattus norvegicus] | 30,2 | 3 |
| gi\|83977457 | 3-hydroxyisobutyrate dehydrogenase type 1 precursor [Rattus norvegicus] | 29,9 | 13 |
| gi\|149046212 | 3-hydroxyisobutyryl-Coenzyme A hydrolase [Rattus norvegicus] | 43,9 | 17 |
| gi\|38511566 | 3-hydroxymethyl-3-methylglutaryl-Coenzyme A lyase [Rattus norvegicus] | 29,9 | 7 |
| gi\|71051684 | 5' nucleotidase, ecto [Rattus norvegicus] | 11,8 | 2 |
| gi\|48675845 | 5-aminoimidazole-4-carboxamide ribonucleotide formyltransferase/IMP cyclohydrolase [Rattus norvegicus] | 39,9 | 14 |
| gi\|862473 | 5'-AMP-activated protein kinase catalytic alpha-2 subunit [Rattus norvegicus] | 10 | 2 |
| gi\|149036104 | 6-phosphogluconolactonase (predicted), isoform CRA_a [Rattus norvegicus] | 25,2 | 4 |
| gi\|91234898 | 84 kDa heat shock protein [Rattus norvegicus] | 59,3 | 56 |
| gi\|149053827 | A kinase (PRKA) anchor protein 1, isoform CRA_b [Rattus norvegicus] | 11,8 | 2 |
| gi\|33086444 | Ab1-021 [Rattus norvegicus] | 37,2 | 19 |
| gi\|33086560 | Ab2-131 [Rattus norvegicus] | 12,1 | 2 |
| gi\|33086600 | Ab2-371 [Rattus norvegicus] | 13 | 3 |
| gi\|81336220 | ABC transporter 8 [Rattus norvegicus] | 29 | 9 |
| gi\|32527721 | Ac2-067 [Rattus norvegicus] | 16,6 | 2 |
| gi\|8392836 | acetyl-Coenzyme A acetyltransferase 1 precursor [Rattus norvegicus] | 59 | 52 |
| gi\|94963134 | acetyl-Coenzyme A carboxylase 2 [Rattus norvegicus] | 14,6 | 6 |
| gi\|56388748 | Acidic (leucine-rich) nuclear phosphoprotein 32 family, member A [Rattus norvegicus] | 33,6 | 5 |
| gi\|8394162 | aconitase 1 [Rattus norvegicus] | 15,4 | 4 |
| gi\|77993370 | actin alpha  cardiac 1 [Rattus norvegicus] | 65,8 | 77 |
| gi\|281332157 | actinin alpha 2 [Rattus norvegicus] | 62,1 | 61 |
| gi\|84781662 | actin-like 6A [Rattus norvegicus] | 7 | 2 |
| gi\|157818027 | acyl-CoA synthetase short-chain family member 1 [Rattus norvegicus] | 26,5 | 13 |

| | | | |
|---|---|---|---|
| gi\|157817043 | acyl-CoA thioesterase 13 [Rattus norvegicus] | 44,3 | 4 |
| gi\|62078649 | acyl-CoA thioesterase 9 [Rattus norvegicus] | 21 | 4 |
| gi\|197313734 | acyl-Coenzyme A dehydrogenase family, member 9 [Rattus norvegicus] | 34,9 | 18 |
| gi\|6978433 | acyl-Coenzyme A dehydrogenase, short/branched chain [Rattus norvegicus] | 23,2 | 2 |
| gi\|188595700 | acylglycerol kinase [Rattus norvegicus] | 14,7 | 3 |
| gi\|157822589 | acylphosphatase 1 [Rattus norvegicus] | 22,2 | 4 |
| gi\|281332093 | acylphosphatase 2, muscle type [Rattus norvegicus] | 50,8 | 8 |
| gi\|8392872 | adaptor-related protein complex 1, beta 1 subunit [Rattus norvegicus] | 17,6 | 4 |
| gi\|157823677 | adaptor-related protein complex 2, alpha 1 subunit [Rattus norvegicus] | 15,4 | 5 |
| gi\|61556832 | adenine phosphoribosyltransferase [Rattus norvegicus] | 40 | 8 |
| gi\|56789878 | Adenosine deaminase [Rattus norvegicus] | 17,6 | 2 |
| gi\|52345435 | adenosine kinase [Rattus norvegicus] | 18 | 4 |
| gi\|149039004 | adenylate kinase 1, isoform CRA_c [Rattus norvegicus] | 69,1 | 23 |
| gi\|8392885 | adenylate kinase 3-like 1 [Rattus norvegicus] | 27,4 | 3 |
| gi\|194473622 | adenylosuccinate lyase [Rattus norvegicus] | 23,4 | 5 |
| gi\|54035294 | Adh5 protein [Rattus norvegicus] | 21,9 | 4 |
| gi\|5002228 | adipocyte lipid-binding protein [Rattus norvegicus] | 65,3 | 26 |
| gi\|62530988 | Adiponectin, C1Q and collagen domain containing [Rattus norvegicus] | 20,9 | 2 |
| gi\|57033190 | ADP-ribosylation factor 3 [Rattus norvegicus] | 28,7 | 7 |
| gi\|56388751 | ADP-ribosylation factor 5 [Rattus norvegicus] | 25 | 4 |
| gi\|149036808 | ADP-ribosylation factor-like 6 interacting protein 5, isoform CRA_b [Rattus norvegicus] | 11,9 | 2 |
| gi\|166797087 | ADP-ribosylhydrolase like 2 [Rattus norvegicus] | 12,2 | 3 |
| gi\|58865636 | ADP-ribosyltransferase 3 [Rattus norvegicus] | 22,2 | 2 |
| gi\|198442897 | AFG3(ATPase family gene 3)-like 2 [Rattus norvegicus] | 41,3 | 21 |
| gi\|38197378 | Ahcy protein [Rattus norvegicus] | 10,7 | 2 |
| gi\|77628163 | AHNAK 1 [Rattus norvegicus] | 44,6 | 13 |
| gi\|976252 | AIR carboxylase-SAICAR synthetase [Rattus norvegicus] | 29,7 | 7 |
| gi\|40352787 | Ak3 protein [Rattus norvegicus] | 43,6 | 7 |
| gi\|149058126 | aldehyde dehydrogenase family 9, subfamily A1 [Rattus norvegicus] | 38,2 | 15 |

| | | | |
|---|---|---|---|
| gi\|55605 | aldehyde dehydrogenase preprotein [Rattus norvegicus] | 53 | 34 |
| gi\|399660 | aldehyde reductase [Rattus norvegicus] | 28,6 | 5 |
| gi\|6978491 | aldo-keto reductase family 1, member B1 [Rattus norvegicus] | 35,4 | 16 |
| gi\|202837 | aldolase A [Rattus norvegicus] | 75,8 | 77 |
| gi\|77539778 | alpha actinin 4 [Rattus norvegicus] | 39,1 | 24 |
| gi\|259953926 | alpha II spectrin [Rattus norvegicus] | 35,5 | 36 |
| gi\|55926139 | alpha isoform of regulatory subunit A, protein phosphatase 2 [Rattus norvegicus] | 27,5 | 10 |
| gi\|205308 | alpha-1 major acute phase protein prepeptide [Rattus norvegicus] | 35,5 | 14 |
| gi\|83816939 | alpha-1-inhibitor III precursor [Rattus norvegicus] | 31,2 | 30 |
| gi\|6978497 | alpha-1-microglobulin/bikunin precursor precursor [Rattus norvegicus] | 13,5 | 2 |
| gi\|58865362 | alpha-2-antiplasmin precursor [Rattus norvegicus] | 20,4 | 5 |
| gi\|6978477 | alpha-2-HS-glycoprotein precursor [Rattus norvegicus] | 27,8 | 12 |
| gi\|785039 | alpha-adducin, normotensive phenotype [Rattus norvegicus] | 20,5 | 3 |
| gi\|157817115 | Alstrom syndrome 1 homolog [Rattus norvegicus] | 11,3 | 2 |
| gi\|75832035 | aminoacyl tRNA synthetase complex-interacting multifunctional protein 1 [Rattus norvegicus] | 16,8 | 2 |
| gi\|7673021 | aminopeptidase A [Rattus norvegicus] | 11,8 | 2 |
| gi\|2039143 | aminopeptidase B [Rattus norvegicus] | 16,2 | 5 |
| gi\|158749540 | aminopeptidase puromycin sensitive [Rattus norvegicus] | 27,4 | 14 |
| gi\|157819187 | amylo-1,6-glucosidase, 4-alpha-glucanotransferase [Rattus norvegicus] | 27,9 | 28 |
| gi\|157822539 | ankyrin 1, erythrocytic [Rattus norvegicus] | 11,5 | 2 |
| gi\|77157800 | ankyrin 3, epithelial isoform 2 [Rattus norvegicus] | 11,5 | 2 |
| gi\|58865414 | annexin A11 [Rattus norvegicus] | 36,2 | 15 |
| gi\|51980303 | Annexin A3 [Rattus norvegicus] | 63 | 17 |
| gi\|47477833 | Annexin A7 [Rattus norvegicus] | 30,9 | 6 |
| gi\|4096754 | anti-NGF30 antibody light-chain [Rattus norvegicus] | 49,1 | 10 |
| gi\|3510374 | antisecretory factor [Rattus norvegicus] | 17,4 | 2 |
| gi\|73695330 | Ap2b1 protein [Rattus norvegicus] | 14,3 | 5 |
| gi\|624915 | APEX nuclease [Rattus norvegicus] | 18 | 2 |
| gi\|6978515 | apolipoprotein A-I precursor [Rattus norvegicus] | 39,8 | 11 |

| | | | |
|---|---|---|---|
| gi\|60552712 | Apolipoprotein A-IV [Rattus norvegicus] | 37,6 | 7 |
| gi\|195540026 | Apolipoprotein B mRNA editing enzyme, catalytic polypeptide-like 2 [Rattus norvegicus] | 31,7 | 3 |
| gi\|57528174 | apolipoprotein H (beta-2-glycoprotein I) [Rattus norvegicus] | 20,6 | 4 |
| gi\|188497679 | apoptosis inhibitor 5 [Rattus norvegicus] | 14,7 | 2 |
| gi\|7619915 | apoptosis-inducing factor [Rattus norvegicus] | 34,2 | 12 |
| gi\|157821685 | arg tyrosine kinase [Rattus norvegicus] | 10,8 | 2 |
| gi\|149021399 | Arg/Abl-interacting protein ArgBP2, isoform CRA_c [Rattus norvegicus] | 18,6 | 8 |
| gi\|171846737 | Arginyl-tRNA synthetase [Rattus norvegicus] | 20,3 | 5 |
| gi\|187469764 | Armet protein [Rattus norvegicus] | 23,5 | 4 |
| gi\|187469602 | ARP1 actin-related protein 1 homolog B (yeast) [Rattus norvegicus] | 18,1 | 3 |
| gi\|914957 | Ash-m [Rattus norvegicus] | 35,5 | 2 |
| gi\|6980972 | aspartate aminotransferase 2 [Rattus norvegicus] | 64,9 | 48 |
| gi\|149016180 | aspartyl aminopeptidase, isoform CRA_b [Rattus norvegicus] | 26 | 8 |
| gi\|55249793 | Asph protein [Rattus norvegicus] | 27,5 | 5 |
| gi\|91177626 | atlastin-3-like [Rattus norvegicus] | 10,3 | 4 |
| gi\|54145376 | ATP synthase F0 subunit 8 [Rattus norvegicus] | 26,9 | 5 |
| gi\|157822047 | ATP synthase mitochondrial F1 complex assembly factor 2 [Rattus norvegicus] | 18,1 | 3 |
| gi\|39645769 | ATP synthase, H+ transporting, mitochondrial F0 complex, subunit B1 [Rattus norvegicus] | 53,5 | 39 |
| gi\|9506411 | ATP synthase, H+ transporting, mitochondrial F0 complex, subunit d [Rattus norvegicus] | 81,4 | 24 |
| gi\|47058994 | ATP synthase, H+ transporting, mitochondrial F0 complex, subunit G [Rattus norvegicus] | 65,1 | 5 |
| gi\|71681130 | ATP synthase, H+ transporting, mitochondrial F1 complex, beta polypeptide [Rattus norvegicus] | 73,9 | 190 |
| gi\|149034587 | ATP synthase, H+ transporting, mitochondrial F1 complex, delta subunit, isoform CRA_b [Rattus norvegicus] | 51,2 | 28 |
| gi\|77917528 | ATPase inhibitory factor 1 precursor [Rattus norvegicus] | 57 | 13 |
| gi\|149053310 | ATPase, Ca++ transporting, ubiquitous, isoform CRA_b [Rattus norvegicus] | 23,6 | 21 |
| gi\|55716047 | ATPase, H transporting, lysosomal V1 subunit B2 [Rattus norvegicus] | 14,7 | 2 |
| gi\|183985826 | ATPase, H transporting, lysosomal V1 subunit G1 [Rattus norvegicus] | 31,4 | 2 |
| gi\|157819953 | ATPase, H+ transporting, lysosomal V1 subunit A [Rattus norvegicus] | 22,2 | 8 |
| gi\|50927657 | ATPase, Na+/K+ transporting, beta 1 polypeptide [Rattus norvegicus] | 33,9 | 6 |
| gi\|58865904 | ATP-binding cassette, sub-family B (MDR/TAP), member 10 [Rattus norvegicus] | 15,7 | 2 |

| | | | |
|---|---|---|---|
| gi\|433211 | ATP-stimulated glucocorticoid-receptor translocaton promoter [Rattus norvegicus] | 12,4 | 4 |
| gi\|149044979 | AU RNA binding protein/enoyl-coenzyme A hydratase (predicted), isoform CRA_a [Rattus norvegicus] | 37,1 | 6 |
| gi\|33086632 | Ba1-651 [Rattus norvegicus] | 10,8 | 10 |
| gi\|7709992 | basigin isoform 2 [Rattus norvegicus] | 41,5 | 18 |
| gi\|51948420 | B-cell receptor-associated protein 31 [Rattus norvegicus] | 25,7 | 6 |
| gi\|108937164 | Bcl-2-interacting death suppressor [Rattus norvegicus] | 34,3 | 9 |
| gi\|157823743 | BCL2-like 13 (apoptosis facilitator) [Rattus norvegicus] | 31,7 | 3 |
| gi\|56090628 | BCS1-like [Rattus norvegicus] | 8,4 | 2 |
| gi\|55575 | beta-actin [Rattus norvegicus] | 65,6 | 60 |
| gi\|56252 | beta-globin [Rattus norvegicus] | 95,9 | 66 |
| gi\|50925455 | Biliverdin reductase A [Rattus norvegicus] | 24,8 | 4 |
| gi\|197246855 | Biliverdin reductase B (flavin reductase (NADPH)) [Rattus norvegicus] | 30,6 | 4 |
| gi\|81295385 | biphenyl hydrolase-like (serine hydrolase) [Rattus norvegicus] | 25,4 | 3 |
| gi\|40786455 | bisphosphoglycerate mutase [Rattus norvegicus] | 11,2 | 2 |
| gi\|157819589 | bolA homolog 3 [Rattus norvegicus] | 40,9 | 2 |
| gi\|66911415 | Brain protein 44-like [Rattus norvegicus] | 45 | 3 |
| gi\|165971320 | Branched chain keto acid dehydrogenase E1, beta polypeptide [Rattus norvegicus] | 36,9 | 6 |
| gi\|62945334 | Bri3 binding protein [Rattus norvegicus] | 24,1 | 2 |
| gi\|60688421 | C9 protein [Rattus norvegicus] | 24,7 | 7 |
| gi\|871525 | Calcium binding protein [Rattus norvegicus] | 41,4 | 6 |
| gi\|157818343 | calcium binding protein 39 [Rattus norvegicus] | 24 | 2 |
| gi\|27450704 | calcium channel alpha-2 delta-1 subunit isoform e [Rattus norvegicus] | 10,7 | 3 |
| gi\|149065235 | caldesmon 1, isoform CRA_a [Rattus norvegicus] | 37,8 | 4 |
| gi\|8394168 | calmodulin 2 [Rattus norvegicus] | 59,1 | 18 |
| gi\|76159282 | calpastatin isoform a [Rattus norvegicus] | 30,3 | 8 |
| gi\|9506501 | calponin 3, acidic [Rattus norvegicus] | 17,6 | 2 |
| gi\|47940716 | Calsequestrin 2 (cardiac muscle) [Rattus norvegicus] | 40,8 | 45 |
| gi\|155369271 | cAMP-dependent protein kinase catalytic subunit alpha [Rattus norvegicus] | 35,3 | 6 |
| gi\|59709467 | CAP, adenylate cyclase-associated protein 1 [Rattus norvegicus] | 12,9 | 4 |

| | | | |
|---|---|---|---|
| gi\|975311 | CAP2 protein [Rattus norvegicus] | 23,9 | 6 |
| gi\|157822193 | carbohydrate kinase domain containing [Rattus norvegicus] | 19,9 | 2 |
| gi\|9506449 | carbonic anhydrase IV precursor [Rattus norvegicus] | 13,3 | 3 |
| gi\|468766 | carboxylesterase; serum carboxylesterase [Rattus norvegicus] | 25,7 | 8 |
| gi\|205495 | cardiac myosin light chain 2 [Rattus norvegicus] | 76,5 | 26 |
| gi\|42417059 | cardiac titin fetal N2BA isoform middle Ig [Rattus norvegicus] | 10,6 | 5 |
| gi\|48735405 | Carnitine palmitoyltransferase 1a, liver [Rattus norvegicus] | 10,9 | 4 |
| gi\|6978607 | catalase [Rattus norvegicus] | 30,7 | 14 |
| gi\|6978681 | catechol-O-methyltransferase [Rattus norvegicus] | 10,2 | 3 |
| gi\|149022412 | catenin (cadherin associated protein), delta 1 (predicted), isoform CRA_b [Rattus norvegicus] | 14,6 | 2 |
| gi\|55742755 | catenin (cadherin-associated protein), alpha 1, 102kDa [Rattus norvegicus] | 23,1 | 10 |
| gi\|82830420 | cathepsin B preproprotein [Rattus norvegicus] | 31,6 | 9 |
| gi\|6978723 | cathepsin L1 preproprotein [Rattus norvegicus] | 18,6 | 2 |
| gi\|575380 | caveolin [Rattus norvegicus] | 37,1 | 12 |
| gi\|9506465 | caveolin 3 [Rattus norvegicus] | 26,5 | 6 |
| gi\|33086660 | Cc1-8 [Rattus norvegicus] | 38,6 | 56 |
| gi\|33086672 | Cc2-27 [Rattus norvegicus] | 13,1 | 7 |
| gi\|6978639 | Cd81 molecule [Rattus norvegicus] | 16,5 | 3 |
| gi\|32965004 | CD99 [Rattus norvegicus] | 22,4 | 2 |
| gi\|38197354 | Cell division cycle 37 homolog (S. cerevisiae) [Rattus norvegicus] | 28,8 | 6 |
| gi\|6531681 | cell division cycle 42 [Rattus norvegicus] | 36,7 | 4 |
| gi\|7339666 | cell membrane glycoprotein 110000Mr (surface antigen) homolog [Rattus norvegicus] | 10,6 | 2 |
| gi\|6707016 | cell surface protein CD36 [Rattus norvegicus] | 40,9 | 10 |
| gi\|8650478 | cellular nucleic acid binding protein [Rattus norvegicus] | 26 | 5 |
| gi\|82830401 | cerebral dopamine neurotrophic factor precursor [Rattus norvegicus] | 26,7 | 3 |
| gi\|9506497 | clathrin, heavy chain (Hc) [Rattus norvegicus] | 26,9 | 23 |
| gi\|203359 | clathryn light chain (LCB2) [Rattus norvegicus] | 54,6 | 8 |
| gi\|56269651 | Clta protein [Rattus norvegicus] | 28,4 | 3 |
| gi\|56972641 | Clybl protein [Rattus norvegicus] | 37,4 | 8 |

| | | | |
|---|---|---|---|
| gi\|158186711 | coagulation factor XIII, A1 subunit precursor [Rattus norvegicus] | 9,3 | 3 |
| gi\|197384345 | coatomer protein complex subunit alpha [Rattus norvegicus] | 11,2 | 2 |
| gi\|157824117 | coatomer protein complex, subunit zeta 1 [Rattus norvegicus] | 17 | 2 |
| gi\|149029695 | coenzyme Q10 homolog A (yeast) (predicted), isoform CRA_a [Rattus norvegicus] | 20,9 | 4 |
| gi\|58865534 | coenzyme Q6 homolog [Rattus norvegicus] | 10,5 | 4 |
| gi\|8393101 | cofilin 1 [Rattus norvegicus] | 59 | 7 |
| gi\|149051244 | cofilin 2, muscle (predicted), isoform CRA_b [Rattus norvegicus] | 56,6 | 11 |
| gi\|149018471 | coiled-coil domain containing 51 [Rattus norvegicus] | 36,8 | 6 |
| gi\|157817027 | coiled-coil-helix-coiled-coil-helix domain containing 3 [Rattus norvegicus] | 64,8 | 19 |
| gi\|48675371 | complement component 1, q subcomponent binding protein precursor [Rattus norvegicus] | 35,8 | 9 |
| gi\|158138561 | complement component 3 [Rattus norvegicus] | 37,4 | 43 |
| gi\|46237589 | complement component 4, gene 1 [Rattus norvegicus] | 12,5 | 3 |
| gi\|157821013 | complement component 8, alpha polypeptide [Rattus norvegicus] | 11,4 | 3 |
| gi\|77861917 | complement factor H [Rattus norvegicus] | 16,2 | 4 |
| gi\|59809147 | Complement factor I [Rattus norvegicus] | 11,8 | 2 |
| gi\|255760059 | COP9 complex subunit 7a isoform 1 [Rattus norvegicus] | 30,9 | 3 |
| gi\|71043620 | COP9 constitutive photomorphogenic homolog subunit 5 [Rattus norvegicus] | 18 | 2 |
| gi\|149045496 | copine III (predicted), isoform CRA_b [Rattus norvegicus] | 14,8 | 2 |
| gi\|79749365 | coproporphyrinogen oxidase precursor [Rattus norvegicus] | 11,7 | 2 |
| gi\|71051329 | Coq3 protein [Rattus norvegicus] | 33,4 | 7 |
| gi\|2996046 | cortactin isoform B [Rattus norvegicus] | 21,5 | 2 |
| gi\|34849861 | Cox6c protein [Rattus norvegicus] | 56,6 | 10 |
| gi\|187469733 | Cox7a2l protein [Rattus norvegicus] | 36,3 | 2 |
| gi\|847719 | CPTI like protein [Rattus norvegicus] | 28,1 | 17 |
| gi\|157817342 | cullin 2 [Rattus norvegicus] | 15,5 | 2 |
| gi\|737713 | Cys-rich protein CRP2 | 42,3 | 12 |
| gi\|6978715 | cystatin B [Rattus norvegicus] | 46,9 | 2 |
| gi\|227013 | cystatin C | 32,5 | 2 |
| gi\|8393206 | cysteine and glycine-rich protein 1 [Rattus norvegicus] | 39,4 | 4 |

| | | | |
|---|---|---|---|
| gi\|56789736 | Cytoglobin [Rattus norvegicus] | 21,6 | 2 |
| gi\|38303959 | Cytochrome b5 reductase 3 [Rattus norvegicus] | 42,2 | 7 |
| gi\|56269768 | Cytochrome b5 type A (microsomal) [Rattus norvegicus] | 51,5 | 6 |
| gi\|48735409 | Cytochrome b5 type B (outer mitochondrial membrane) [Rattus norvegicus] | 47,3 | 4 |
| gi\|5851903 | cytochrome C oxidase assembly protein COX17 [Rattus norvegicus] | 66,7 | 7 |
| gi\|54145375 | cytochrome c oxidase subunit 2 [Rattus norvegicus] | 32,2 | 15 |
| gi\|8393180 | cytochrome c oxidase subunit IV isoform 1 precursor [Rattus norvegicus] | 62,1 | 67 |
| gi\|55971 | cytochrome c oxidase subunit Va preprotein [Rattus norvegicus] | 69,9 | 37 |
| gi\|55992 | cytochrome c oxidase subunit VIa (AA 1 - 118) [Rattus norvegicus] | 61,5 | 50 |
| gi\|56025 | cytochrome c oxidase subunit VIIa [Rattus norvegicus] | 37,4 | 10 |
| gi\|32966975 | cytochrome c oxidase subunit VIIb [Rattus norvegicus] | 21,3 | 2 |
| gi\|77736544 | cytochrome c oxidase, subunit VIa, polypeptide 1 precursor [Rattus norvegicus] | 34,2 | 3 |
| gi\|223718723 | cytochrome c oxidase, subunit VIb polypeptide 1 [Rattus norvegicus] | 80,2 | 31 |
| gi\|197927439 | cytochrome c oxidase, subunit VIIc [Rattus norvegicus] | 36,5 | 11 |
| gi\|6978725 | cytochrome c, somatic [Rattus norvegicus] | 71,4 | 20 |
| gi\|149066116 | cytochrome c-1 (predicted), isoform CRA_c [Rattus norvegicus] | 45,4 | 32 |
| gi\|157823877 | cytoskeleton-associated protein 4 [Rattus norvegicus] | 32,8 | 4 |
| gi\|537941 | cytosolic NADP-dependent isocitrate dehydrogenase [Rattus norvegicus] | 45,9 | 17 |
| gi\|33086684 | Da1-24 [Rattus norvegicus] | 13,4 | 3 |
| gi\|149062405 | damage-specific DNA binding protein 1 [Rattus norvegicus] | 14,6 | 7 |
| gi\|47938978 | Dars protein [Rattus norvegicus] | 19,6 | 3 |
| gi\|56090441 | DEAD (Asp-Glu-Ala-Asp) box polypeptide 5 [Rattus norvegicus] | 19,7 | 2 |
| gi\|56057 | decorin [Rattus norvegicus] | 15,1 | 3 |
| gi\|6978483 | delta-aminolevulinic acid dehydratase [Rattus norvegicus] | 18,5 | 2 |
| gi\|198386317 | delta-sarcoglycan b [Rattus norvegicus] | 14,5 | 2 |
| gi\|149068155 | demethyl-Q 7, isoform CRA_c [Rattus norvegicus] | 39,2 | 4 |
| gi\|149036525 | deoxyguanosine kinase (predicted), isoform CRA_b [Rattus norvegicus] | 23,5 | 3 |
| gi\|24637713 | dermcidin precursor [Rattus norvegicus] | 63,6 | 5 |
| gi\|38197676 | Desmin [Rattus norvegicus] | 51,2 | 14 |

| | | | |
|---|---|---|---|
| gi\|75991707 | destrin [Rattus norvegicus] | 46,7 | 9 |
| gi\|6492122 | deubiquitinating enzyme Ubp109 [Rattus norvegicus] | 14,1 | 2 |
| gi\|56605642 | diablo [Rattus norvegicus] | 33,3 | 4 |
| gi\|54261671 | Diazepam binding inhibitor (GABA receptor modulator, acyl-Coenzyme A binding protein) [Rattus norvegicus] | 74,7 | 8 |
| gi\|183985854 | Dihydrolipoamide branched chain transacylase E2 [Rattus norvegicus] | 26,1 | 10 |
| gi\|56676360 | dihydroorotate dehydrogenase precursor [Rattus norvegicus] | 20,3 | 2 |
| gi\|157786744 | dihydropyrimidinase-like 2 [Rattus norvegicus] | 32,9 | 11 |
| gi\|6978773 | dipeptidylpeptidase 4 [Rattus norvegicus] | 8,2 | 2 |
| gi\|149060747 | discs, large homolog 1 (Drosophila), isoform CRA_e [Rattus norvegicus] | 13,3 | 2 |
| gi\|6012071 | dithiolethione-inducible gene-1 [Rattus norvegicus] | 26,8 | 6 |
| gi\|224549858 | DNA-directed polymerase delta interacting protein 2 [Rattus norvegicus] | 12,2 | 2 |
| gi\|70794764 | DnaJ (Hsp40) homolog, subfamily A, member 4 [Rattus norvegicus] | 21,3 | 9 |
| gi\|61556870 | DnaJ (Hsp40) homolog, subfamily B, member 4 [Rattus norvegicus] | 25,5 | 2 |
| gi\|56799412 | DnaJ subfamily A member 2 [Rattus norvegicus] | 21,4 | 3 |
| gi\|149020488 | dynamin 2, isoform CRA_c [Rattus norvegicus] | 15,7 | 3 |
| gi\|50926121 | Dync1i2 protein [Rattus norvegicus] | 19 | 5 |
| gi\|294543 | dynein heavy chain [Rattus norvegicus] | 25,1 | 37 |
| gi\|157823277 | dysferlin [Rattus norvegicus] | 19,2 | 12 |
| gi\|52630316 | dystrophin isoform Dp71ab [Rattus norvegicus] | 24,6 | 7 |
| gi\|157821387 | early endosome antigen 1 [Rattus norvegicus] | 34,5 | 4 |
| gi\|72255531 | EF-hand domain family, member D2 [Rattus norvegicus] | 42,7 | 4 |
| gi\|194018463 | EH domain binding protein 1-like 1 isoform 2 [Rattus norvegicus] | 18,5 | 4 |
| gi\|71043892 | echinoderm microtubule associated protein like 1 [Rattus norvegicus] | 19,7 | 6 |
| gi\|149056792 | echinoderm microtubule associated protein like 2, isoform CRA_a [Rattus norvegicus] | 15,8 | 2 |
| gi\|52138635 | electron-transferring-flavoprotein dehydrogenase precursor [Rattus norvegicus] | 48,7 | 48 |
| gi\|58865872 | enabled homolog [Rattus norvegicus] | 21,3 | 2 |
| gi\|77917570 | endonuclease G [Rattus norvegicus] | 38,8 | 7 |
| gi\|60688521 | Endoplasmic reticulum protein 29 [Rattus norvegicus] | 25,8 | 2 |
| gi\|77020283 | endosulfine alpha isoform 1 [Rattus norvegicus] | 39,3 | 5 |

| | | | |
|---|---|---|---|
| gi\|50300479 | enthoprotin [Rattus norvegicus] | 16,5 | 2 |
| gi\|51948384 | ErbB3-binding protein 1 [Rattus norvegicus] | 23,1 | 6 |
| gi\|85057089 | Eukaryotic translation elongation factor 1 alpha 1 [Rattus norvegicus] | 38,5 | 16 |
| gi\|61556967 | eukaryotic translation elongation factor 1 delta [Rattus norvegicus] | 23,2 | 6 |
| gi\|71051349 | Eukaryotic translation elongation factor 1 gamma [Rattus norvegicus] | 33,2 | 7 |
| gi\|8393296 | eukaryotic translation elongation factor 2 [Rattus norvegicus] | 42,2 | 27 |
| gi\|9506571 | eukaryotic translation initiation factor 2, subunit 1 alpha [Rattus norvegicus] | 22,9 | 3 |
| gi\|40786451 | eukaryotic translation initiation factor 2, subunit 2 beta [Rattus norvegicus] | 20,7 | 2 |
| gi\|261337190 | eukaryotic translation initiation factor 3, subunit 10 (theta) [Rattus norvegicus] | 36,5 | 5 |
| gi\|149065953 | eukaryotic translation initiation factor 3, subunit 6 interacting protein [Rattus norvegicus] | 19,5 | 5 |
| gi\|40786436 | eukaryotic translation initiation factor 4A1 [Rattus norvegicus] | 32,5 | 3 |
| gi\|56605726 | eukaryotic translation initiation factor 4B [Rattus norvegicus] | 33,7 | 3 |
| gi\|56540868 | Eukaryotic translation initiation factor 4E [Rattus norvegicus] | 31,8 | 2 |
| gi\|9910214 | eukaryotic translation initiation factor 5 [Rattus norvegicus] | 19,6 | 3 |
| gi\|75832132 | extended synaptotagmin-like protein 1 [Rattus norvegicus] | 14,3 | 2 |
| gi\|72255551 | family with sequence similarity 82, member B [Rattus norvegicus] | 37,7 | 6 |
| gi\|83320094 | far upstream element (FUSE) binding protein 1 [Rattus norvegicus] | 18 | 5 |
| gi\|204080 | fatty acid binding protein [Rattus norvegicus] | 93,2 | 57 |
| gi\|58865400 | fermitin family homolog 2 [Rattus norvegicus] | 17,7 | 6 |
| gi\|57033194 | Ferredoxin reductase [Rattus norvegicus] | 17 | 2 |
| gi\|157823017 | ferrochelatase [Rattus norvegicus] | 14,2 | 2 |
| gi\|6562849 | fetuin-like protein IRL685 [Rattus norvegicus] | 10,3 | 4 |
| gi\|56797757 | fibrinogen alpha chain isoform 1 [Rattus norvegicus] | 29,2 | 15 |
| gi\|56971493 | Fibrinogen beta chain [Rattus norvegicus] | 25,5 | 9 |
| gi\|61098186 | fibrinogen gamma chain [Rattus norvegicus] | 14 | 6 |
| gi\|6978839 | fibroblast growth factor 1 precursor [Rattus norvegicus] | 29 | 3 |
| gi\|763182 | fibroblast tropomyosin 4 [Rattus norvegicus] | 67,7 | 25 |
| gi\|189011620 | fibulin 1 [Rattus norvegicus] | 13,5 | 2 |
| gi\|224472721 | filamin alpha [Rattus norvegicus] | 16,9 | 20 |

| | | | |
|---|---|---|---|
| gi\|157818975 | filamin, beta [Rattus norvegicus] | 11,9 | 8 |
| gi\|206725535 | FK506 binding protein 3, 25kDa [Rattus norvegicus] | 36,6 | 4 |
| gi\|157818687 | four and a half LIM domains 3 [Rattus norvegicus] | 9 | 3 |
| gi\|157817879 | fructosamine-3-kinase [Rattus norvegicus] | 19,2 | 4 |
| gi\|6978487 | fructose-bisphosphate aldolase A [Rattus norvegicus] | 78 | 74 |
| gi\|83404987 | Fth1 protein [Rattus norvegicus] | 27,6 | 4 |
| gi\|227665 | fumarase | 51,3 | 37 |
| gi\|209364562 | FUN14 domain containing 2 [Rattus norvegicus] | 33,1 | 2 |
| gi\|55250714 | G elongation factor, mitochondrial 1 [Rattus norvegicus] | 21,8 | 7 |
| gi\|157818483 | G1 to S phase transition 2 [Rattus norvegicus] | 15,5 | 3 |
| gi\|55741776 | gamma sarcoglycan [Rattus norvegicus] | 10,3 | 2 |
| gi\|41059678 | gamma synuclein [Rattus norvegicus] | 39,8 | 2 |
| gi\|1220488 | gamma-2a immunoglobulin heavy chain [Rattus norvegicus] | 35,2 | 27 |
| gi\|6978896 | gap junction protein, alpha 1 [Rattus norvegicus] | 21,2 | 6 |
| gi\|40254781 | GDP dissociation inhibitor 2 [Rattus norvegicus] | 45,2 | 7 |
| gi\|516540 | GDP-dissociation inhibitor [Rattus norvegicus] | 39,2 | 7 |
| gi\|205517 | general mitochondrial matrix processing protease 55 kDa subunit [Rattus norvegicus] | 20,6 | 5 |
| gi\|77799118 | GIMAP4 [Rattus norvegicus] | 28,1 | 4 |
| gi\|62945328 | glioblastoma amplified sequence [Rattus norvegicus] | 35,2 | 15 |
| gi\|62078447 | globin, alpha [Rattus norvegicus] | 45,1 | 7 |
| gi\|281332160 | glucan (1,4-alpha-), branching enzyme 1 [Rattus norvegicus] | 17 | 6 |
| gi\|56090285 | glutaminyl-tRNA synthetase [Rattus norvegicus] | 24,5 | 5 |
| gi\|66793366 | glutamyl-prolyl-tRNA synthetase [Rattus norvegicus] | 17,4 | 6 |
| gi\|78187979 | glutaredoxin 3 [Rattus norvegicus] | 26,1 | 4 |
| gi\|157823513 | glutaredoxin 5 [Rattus norvegicus] | 42,8 | 3 |
| gi\|157820807 | glutaryl-Coenzyme A dehydrogenase [Rattus norvegicus] | 16,1 | 2 |
| gi\|68138297 | glutathione peroxidase [Rattus norvegicus] | 61,7 | 21 |
| gi\|90903229 | glutathione peroxidase 4 isoform B precursor [Rattus norvegicus] | 21,7 | 3 |
| gi\|7188365 | glutathione S-transferase alpha [Rattus norvegicus] | 24,8 | 2 |

| | | | |
|---|---|---|---|
| gi\|208969735 | glutathione S-transferase alpha 3 [Rattus norvegicus] | 15,3 | 3 |
| gi\|8393502 | glutathione S-transferase mu 1 [Rattus norvegicus] | 43,6 | 6 |
| gi\|28933457 | glutathione S-transferase mu 2 [Rattus norvegicus] | 67,9 | 23 |
| gi\|66730313 | glutathione S-transferase mu 4 [Rattus norvegicus] | 54,6 | 6 |
| gi\|56090550 | glutathione S-transferase omega 1 [Rattus norvegicus] | 21,2 | 4 |
| gi\|769703 | glutathione S-transferase subunit Yrs [Rattus norvegicus] | 27,1 | 4 |
| gi\|529588 | glutathione S-transferase Yb3 subunit [Rattus norvegicus] | 52,8 | 12 |
| gi\|57527919 | glycerol-3-phosphate dehydrogenase 1 (soluble) [Rattus norvegicus] | 21,5 | 7 |
| gi\|6980978 | glycerol-3-phosphate dehydrogenase 2, mitochondrial precursor [Rattus norvegicus] | 11 | 2 |
| gi\|204421 | glycogen phosphorylase [Rattus norvegicus] | 52 | 38 |
| gi\|37589607 | Glycoprotein, synaptic 2 [Rattus norvegicus] | 11 | 2 |
| gi\|506417 | glypican [Rattus norvegicus] | 28,1 | 7 |
| gi\|253970435 | GNAS complex locus XLas [Rattus norvegicus] | 17,4 | 5 |
| gi\|149038203 | golgi apparatus protein 1 [Rattus norvegicus] | 15,7 | 2 |
| gi\|224451084 | G-protein signaling modulator 1 (AGS3-like, C. elegans) isoform a [Rattus norvegicus] | 29 | 3 |
| gi\|8980841 | GRIP-associated protein 1 long form [Rattus norvegicus] | 26,2 | 3 |
| gi\|6978879 | group specific component precursor [Rattus norvegicus] | 18,5 | 6 |
| gi\|67678103 | GrpE-like 1, mitochondrial [Rattus norvegicus] | 46,5 | 6 |
| gi\|281306781 | GTPase activating protein (SH3 domain) binding protein 1 [Rattus norvegicus] | 17 | 3 |
| gi\|8394152 | GTP-binding protein alpha o [Rattus norvegicus] | 28,3 | 2 |
| gi\|9837357 | GTP-binding protein RAB11B [Rattus norvegicus] | 40,8 | 12 |
| gi\|9837359 | GTP-binding protein RAB7 [Rattus norvegicus] | 30 | 3 |
| gi\|71089913 | guanine nucleotide binding protein alpha inhibiting 2 [Rattus norvegicus] | 37,8 | 8 |
| gi\|66911967 | Hagh protein [Rattus norvegicus] | 22,7 | 4 |
| gi\|204655 | haptoglobin (Hp) [Rattus norvegicus] | 25,1 | 11 |
| gi\|157824216 | Harvey rat sarcoma virus oncogene, subgroup R [Rattus norvegicus] | 34,9 | 3 |
| gi\|847785 | HASPP28 [Rattus norvegicus] | 39,2 | 4 |
| gi\|157823683 | HD domain containing 2 [Rattus norvegicus] | 29,2 | 2 |
| gi\|56383 | heat shock protein (hsp60) precursor [Rattus norvegicus] | 62,3 | 49 |

| | | | |
|---|---|---|---|
| gi\|197927441 | heat shock protein 4 like [Rattus norvegicus] | 16,5 | 3 |
| gi\|38303969 | Heat shock protein 5 [Rattus norvegicus] | 52,3 | 35 |
| gi\|197246889 | Heat shock protein family, member 7 (cardiovascular) [Rattus norvegicus] | 56,8 | 6 |
| gi\|195540095 | Hebp1 protein [Rattus norvegicus] | 14,7 | 2 |
| gi\|157822045 | hedgehog acyltransferase-like [Rattus norvegicus] | 19,7 | 6 |
| gi\|6981010 | hemoglobin alpha 1 chain [Rattus norvegicus] | 74,7 | 78 |
| gi\|60688311 | Hemopexin [Rattus norvegicus] | 35,7 | 15 |
| gi\|56388805 | Hepatoma-derived growth factor, related protein 2 [Rattus norvegicus] | 19,9 | 2 |
| gi\|34327779 | heterogeneous nuclear ribonucleoprotein A3 variant b [Rattus norvegicus] | 26,6 | 5 |
| gi\|71043810 | heterogeneous nuclear ribonucleoprotein C (C1/C2) [Rattus norvegicus] | 29,5 | 2 |
| gi\|76096336 | heterogeneous nuclear ribonucleoprotein D-like [Rattus norvegicus] | 25,5 | 3 |
| gi\|82546819 | heterogeneous nuclear ribonucleoprotein F [Rattus norvegicus] | 21,5 | 4 |
| gi\|197927211 | heterogeneous nuclear ribonucleoprotein L isoform a [Rattus norvegicus] | 20,6 | 2 |
| gi\|158186698 | heterogeneous nuclear ribonucleoprotein M isoform a [Rattus norvegicus] | 22,8 | 6 |
| gi\|62990189 | heterogeneous nuclear ribonucleoprotein R [Rattus norvegicus] | 13,8 | 2 |
| gi\|6562845 | heterogeneous nuclear ribonucleoprotein; type A/B hnRNP p40 [Rattus norvegicus] | 29,5 | 4 |
| gi\|7329187 | heterotrimeric guanine nucleotide-binding protein alpha q subunit [Rattus norvegicus] | 16,7 | 2 |
| gi\|7549765 | hexokinase 2 [Rattus norvegicus] | 29,6 | 14 |
| gi\|183986567 | HIG1 domain family, member 2A [Rattus norvegicus] | 33 | 2 |
| gi\|76779390 | High density lipoprotein binding protein (vigilin) [Rattus norvegicus] | 20,3 | 5 |
| gi\|164565401 | histidine triad nucleotide binding protein 2 [Rattus norvegicus] | 35,6 | 3 |
| gi\|70794762 | histidyl-tRNA synthetase [Rattus norvegicus] | 32,1 | 6 |
| gi\|72679585 | Histone cluster 1, H4b [Rattus norvegicus] | 24,3 | 2 |
| gi\|77020274 | HLA-B-associated transcript 3 isoform 1 [Rattus norvegicus] | 11,9 | 3 |
| gi\|71121745 | Hnrph1 protein [Rattus norvegicus] | 27,7 | 2 |
| gi\|38197650 | Hnrpk protein [Rattus norvegicus] | 41,4 | 11 |
| gi\|9624483 | hormone-regulated proliferation-associated 20 kDa protein short form [Rattus norvegicus] | 34,5 | 3 |
| gi\|951425 | housekeeping protein [Rattus norvegicus] | 69,5 | 18 |
| gi\|68533980 | Hras protein [Rattus norvegicus] | 31,5 | 3 |

| | | | |
|---|---|---|---|
| gi\|56385 | Hsc70-ps1 [Rattus norvegicus] | 63,5 | 77 |
| gi\|157820565 | HscB iron-sulfur cluster co-chaperone homolog [Rattus norvegicus] | 18,8 | 3 |
| gi\|48734844 | Hsd17b4 protein [Rattus norvegicus] | 20,2 | 7 |
| gi\|215272398 | huntingtin interacting protein 1 [Rattus norvegicus] | 16,4 | 2 |
| gi\|281306811 | hydrogen/potassium-exchanging ATPase 4A [Rattus norvegicus] | 17,3 | 5 |
| gi\|78214363 | hydroxymethylbilane synthase [Rattus norvegicus] | 28,3 | 3 |
| gi\|157787020 | hypothetical protein LOC289278 [Rattus norvegicus] | 31,6 | 3 |
| gi\|157821125 | hypothetical protein LOC298377 [Rattus norvegicus] | 24,7 | 2 |
| gi\|77627996 | hypothetical protein LOC298384 [Rattus norvegicus] | 43,1 | 5 |
| gi\|197386048 | hypothetical protein LOC298861 [Rattus norvegicus] | 12,1 | 2 |
| gi\|187282120 | hypothetical protein LOC299909 [Rattus norvegicus] | 19,4 | 2 |
| gi\|157816969 | hypothetical protein LOC307067 [Rattus norvegicus] | 8,1 | 3 |
| gi\|149022761 | hypothetical protein LOC311254 [Rattus norvegicus] | 37,4 | 7 |
| gi\|157819345 | hypothetical protein LOC313776 [Rattus norvegicus] | 10,2 | 4 |
| gi\|157820257 | hypothetical protein LOC314214 [Rattus norvegicus] | 15,5 | 2 |
| gi\|157819293 | hypothetical protein LOC360617 [Rattus norvegicus] | 33 | 2 |
| gi\|187937032 | hypothetical protein LOC361181 [Rattus norvegicus] | 22 | 2 |
| gi\|157817209 | hypothetical protein LOC361606 [Rattus norvegicus] | 38,7 | 2 |
| gi\|157819755 | hypothetical protein LOC364073 [Rattus norvegicus] | 20 | 2 |
| gi\|157817241 | hypothetical protein LOC500694 [Rattus norvegicus] | 38,3 | 5 |
| gi\|155369672 | hypothetical protein LOC500874 [Rattus norvegicus] | 23,4 | 5 |
| gi\|207028435 | hypothetical protein LOC684352 [Rattus norvegicus] | 28,9 | 6 |
| gi\|169234852 | hypothetical protein LOC687565 [Rattus norvegicus] | 25,3 | 3 |
| gi\|18041977 | hypothetical RNA binding protein RDA288 [Rattus norvegicus] | 26 | 2 |
| gi\|68534712 | Hypoxanthine phosphoribosyltransferase 1 [Rattus norvegicus] | 62,4 | 15 |
| gi\|77404380 | hypoxia up-regulated 1 [Rattus norvegicus] | 23,6 | 5 |
| gi\|223365746 | Chain A, Crystal Structure Analysis Of The Sodium-Bound Annexin A4 At 1.34 A Resolution | 49,4 | 10 |
| gi\|99032341 | Chain A, Crystal Structure Of Erk2 Complex With Kim Peptide Derived From Mkp3 | 19,2 | 2 |
| gi\|9257037 | Chain A, Crystal Structure Of M-Calpain | 20,9 | 7 |

| | | | |
|---|---|---|---|
| gi\|6435548 | Chain B, Crystal Structure Of A Mammalian 2-Cys Peroxiredoxin, Hbp23. | 41,7 | 13 |
| gi\|42543460 | Chain B, Crystal Structure Of A Mu-Like Calpain | 30,7 | 8 |
| gi\|3318959 | Chain B, Three-Dimensional Structure Of Nadph-Cytochrome P450 Reductase: Prototype For Fmn-And Fad-Containing Enzymes | 15,9 | 4 |
| gi\|7546397 | Chain C, Crystal Structure Of Rab Geranylgeranyltransferase From Rat Brain | 18,5 | 3 |
| gi\|42543496 | Chain D, Crystal Structure Of Mitochondrial Class Kappa Glutathione Transferase | 26,1 | 3 |
| gi\|76253725 | chaperonin containing Tcp1, subunit 6A (zeta 1) [Rattus norvegicus] | 25,4 | 6 |
| gi\|157819651 | chaperonin containing Tcp1, subunit 7 (eta) [Rattus norvegicus] | 26,7 | 4 |
| gi\|149059759 | chaperonin subunit 8 (theta) (predicted), isoform CRA_a [Rattus norvegicus] | 33,2 | 13 |
| gi\|313804 | CHIP28 [Rattus norvegicus] | 18,6 | 2 |
| gi\|149024253 | chloride intracellular channel 4, isoform CRA_b [Rattus norvegicus] | 41,1 | 11 |
| gi\|157820325 | chromosome segregation 1-like [Rattus norvegicus] | 18,4 | 4 |
| gi\|1220490 | Ig kappa chain [Rattus norvegicus] | 34,5 | 12 |
| gi\|623560 | immunoglobulin gamma-2b [Rattus norvegicus] | 24,7 | 9 |
| gi\|89258023 | immunoglobulin lambda light chain [Rattus norvegicus] | 29,8 | 3 |
| gi\|501064 | immunophilin FKBP12 [Rattus norvegicus] | 35,2 | 8 |
| gi\|157820315 | importin 7 [Rattus norvegicus] | 11,4 | 2 |
| gi\|149036390 | inner membrane protein, mitochondrial, isoform CRA_a [Rattus norvegicus] | 49,7 | 48 |
| gi\|40018566 | inosine monophosphate dehydrogenase 2 [Rattus norvegicus] | 15 | 2 |
| gi\|149061428 | inositol polyphosphate-5-phosphatase A (predicted), isoform CRA_b [Rattus norvegicus] | 16,6 | 2 |
| gi\|6981076 | insulin-degrading enzyme [Rattus norvegicus] | 14,9 | 2 |
| gi\|149027462 | insulin-like growth factor 2 receptor, isoform CRA_b [Rattus norvegicus] | 5,8 | 2 |
| gi\|197927176 | integral membrane protein 1 [Rattus norvegicus] | 10,6 | 2 |
| gi\|57528941 | integrin alpha 7 precursor [Rattus norvegicus] | 16,2 | 7 |
| gi\|158303324 | integrin beta 1 [Rattus norvegicus] | 20,6 | 9 |
| gi\|149022213 | integrin, alpha 6, isoform CRA_b [Rattus norvegicus] | 15,1 | 2 |
| gi\|59808174 | Inter alpha-trypsin inhibitor, heavy chain 4 [Rattus norvegicus] | 24,4 | 9 |
| gi\|8393899 | inter-alpha trypsin inhibitor, heavy chain 3 precursor [Rattus norvegicus] | 12,1 | 3 |
| gi\|210032529 | IQ motif containing GTPase activating protein 1 [Rattus norvegicus] | 15 | 3 |
| gi\|187469195 | Iron-sulfur cluster scaffold homolog (E. coli) [Rattus norvegicus] | 32,3 | 3 |

| | | | |
|---|---|---|---|
| gi\|198278545 | isoamyl acetate-hydrolyzing esterase 1 homolog [Rattus norvegicus] | 15,3 | 2 |
| gi\|149041699 | isocitrate dehydrogenase 3 (NAD+) alpha, isoform CRA_a [Rattus norvegicus] | 39,8 | 23 |
| gi\|208022685 | isoleucine tRNA synthetase [Rattus norvegicus] | 11,3 | 3 |
| gi\|6981112 | isovaleryl Coenzyme A dehydrogenase precursor [Rattus norvegicus] | 40,1 | 23 |
| gi\|83816931 | junctophilin 2 [Rattus norvegicus] | 24,6 | 7 |
| gi\|8393610 | karyopherin (importin) beta 1 [Rattus norvegicus] | 14,3 | 6 |
| gi\|62339392 | karyopherin alpha 3 [Rattus norvegicus] | 10,4 | 2 |
| gi\|62339366 | karyopherin alpha 4 (importin alpha 3) [Rattus norvegicus] | 10,9 | 2 |
| gi\|17105344 | kelch repeat and BTB (POZ) domain containing 10 [Rattus norvegicus] | 21 | 5 |
| gi\|157823427 | kelch-like 31 [Rattus norvegicus] | 9,9 | 2 |
| gi\|120474989 | keratin 1 [Rattus norvegicus] | 32,8 | 30 |
| gi\|56847618 | keratin 16 [Rattus norvegicus] | 33,7 | 9 |
| gi\|57012352 | keratin 75 [Rattus norvegicus] | 36,1 | 16 |
| gi\|83776543 | kinesin family member 5B [Rattus norvegicus] | 32,2 | 11 |
| gi\|80861401 | kininogen 1 [Rattus norvegicus] | 35,1 | 15 |
| gi\|7549773 | kininogen 2 isoform 1 [Rattus norvegicus] | 14,9 | 5 |
| gi\|6318318 | kynurenine aminotransferase/glutamine transaminase K [Rattus norvegicus] | 14,2 | 3 |
| gi\|157820173 | L-2-hydroxyglutarate dehydrogenase [Rattus norvegicus] | 24,4 | 7 |
| gi\|8393706 | lactate dehydrogenase A [Rattus norvegicus] | 51,2 | 47 |
| gi\|149038217 | lactate dehydrogenase D, isoform CRA_d [Rattus norvegicus] | 21,8 | 4 |
| gi\|83320101 | lactation elevated 1 [Rattus norvegicus] | 27,5 | 3 |
| gi\|763407 | lamina associated polypeptide 2 [Rattus norvegicus] | 18,1 | 2 |
| gi\|8393693 | laminin receptor 1 [Rattus norvegicus] | 24,8 | 5 |
| gi\|157818227 | laminin, beta 1 [Rattus norvegicus] | 6 | 2 |
| gi\|149018527 | laminin, beta 2 [Rattus norvegicus] | 19,7 | 9 |
| gi\|281371490 | laminin, gamma 1 [Rattus norvegicus] | 28,6 | 20 |
| gi\|6625487 | lanthionine synthetase C-like protein 1 [Rattus norvegicus] | 19,3 | 5 |
| gi\|54035563 | Ldb3 protein [Rattus norvegicus] | 33,2 | 18 |
| gi\|6981154 | lectin, galactose binding, soluble 5 [Rattus norvegicus] | 23,5 | 3 |

| | | | |
|---|---|---|---|
| gi\|9845261 | lectin, galactoside-binding, soluble, 1 [Rattus norvegicus] | 54,8 | 15 |
| gi\|169234844 | lectin, mannose-binding 2 [Rattus norvegicus] | 17,9 | 4 |
| gi\|149064501 | lectin, mannose-binding, 1, isoform CRA_a [Rattus norvegicus] | 30,8 | 6 |
| gi\|687712 | lens epithelial protein [Rattus norvegicus] | 32 | 5 |
| gi\|71795621 | leukotriene A4 hydrolase [Rattus norvegicus] | 14,6 | 2 |
| gi\|397357 | L-iditol 2-dehydrogenase [Rattus norvegicus] | 25,3 | 5 |
| gi\|56676346 | LIM and cysteine-rich domains 1 [Rattus norvegicus] | 33,7 | 9 |
| gi\|71122452 | LIM and SH3 protein 1 [Rattus norvegicus] | 32,7 | 4 |
| gi\|149022281 | limb and neural patterns (predicted), isoform CRA_b [Rattus norvegicus] | 14,6 | 3 |
| gi\|197246745 | Limd1 protein [Rattus norvegicus] | 11,5 | 2 |
| gi\|533124 | lipid-binding protein [Rattus norvegicus] | 30,4 | 4 |
| gi\|6981168 | lipoprotein lipase precursor [Rattus norvegicus] | 14,6 | 2 |
| gi\|6981146 | L-lactate dehydrogenase B [Rattus norvegicus] | 72,2 | 69 |
| gi\|71051822 | LOC683313 protein [Rattus norvegicus] | 44 | 22 |
| gi\|75517618 | LOC684538 protein [Rattus norvegicus] | 25,7 | 2 |
| gi\|5759131 | low molecular weight protein tyrosine phosphatase isoform A [Rattus norvegicus] | 42,4 | 4 |
| gi\|45478084 | LRRG00122 [Rattus norvegicus] | 18,5 | 2 |
| gi\|37654320 | LRRGT00052 [Rattus norvegicus] | 12,1 | 9 |
| gi\|45478238 | LRRGT00199 [Rattus norvegicus] | 31,6 | 36 |
| gi\|187469483 | LUC7-like 2 (S. cerevisiae) [Rattus norvegicus] | 24,5 | 2 |
| gi\|643024 | lumican, secretory intersticial proteoglycan [Rattus norvegicus] | 17,8 | 6 |
| gi\|58865656 | lymphocyte cytosolic protein 1 [Rattus norvegicus] | 22,2 | 4 |
| gi\|6981362 | lysophospholipase 1 [Rattus norvegicus] | 23 | 3 |
| gi\|55741637 | lysyl-tRNA synthetase [Rattus norvegicus] | 23,6 | 5 |
| gi\|49256645 | Major vault protein [Rattus norvegicus] | 24 | 8 |
| gi\|42476181 | malate dehydrogenase, mitochondrial precursor [Rattus norvegicus] | 82,5 | 104 |
| gi\|871528 | mammalian fusca gene homologue [Rattus norvegicus] | 20,2 | 2 |
| gi\|205360969 | maternal embryonic message 3 [Rattus norvegicus] | 20,4 | 9 |
| gi\|1871633 | Mature alpha chain of major histocompatibility complex class I antigen [Rattus norvegicus] | 42,3 | 8 |

| | | | |
|---|---|---|---|
| gi\|2168158 | Mature alpha chain of major histocompatibility complex class I antigen [Rattus norvegicus] | 30,3 | 3 |
| gi\|55824737 | Mercaptopyruvate sulfurtransferase [Rattus norvegicus] | 32 | 4 |
| gi\|149066556 | metadherin, isoform CRA_b [Rattus norvegicus] | 11,8 | 2 |
| gi\|56605654 | metaxin 2 [Rattus norvegicus] | 19 | 5 |
| gi\|59808745 | methenyltetrahydrofolate cyclohydrolase, formyltetrahydrofolate synthetase [Rattus norvegicus] | 21,5 | 4 |
| gi\|157821869 | methylmalonyl CoA epimerase [Rattus norvegicus] | 14,6 | 4 |
| gi\|710563 | MHC class I protein [Rattus norvegicus] | 32,2 | 5 |
| gi\|78097100 | microtubule-associated protein, RP/EB family, member 1 [Rattus norvegicus] | 15,3 | 2 |
| gi\|2780408 | MIPP65 [Rattus norvegicus] | 44,5 | 12 |
| gi\|8393331 | mitogen activated protein kinase 3 [Rattus norvegicus] | 20,5 | 2 |
| gi\|8394233 | mitogen-activated protein kinase 9 [Rattus norvegicus] | 18 | 3 |
| gi\|38303967 | Mitogen-activated protein kinase-activated protein kinase 2 [Rattus norvegicus] | 14,8 | 3 |
| gi\|165971637 | Mitochondrial carrier homolog 2 (C. elegans) [Rattus norvegicus] | 31 | 3 |
| gi\|206597496 | mitochondrial isoleucine tRNA synthetase [Rattus norvegicus] | 15,9 | 3 |
| gi\|239049264 | mitochondrial malic enzyme 3 [Rattus norvegicus] | 30,9 | 9 |
| gi\|599963 | mitochondrial oxidative phosphorylation coupling factor 6 [Rattus norvegicus] | 51,9 | 34 |
| gi\|397699 | mitochondrial processing protease [Rattus norvegicus] | 20,9 | 2 |
| gi\|55741522 | mitochondrial protein 18 kDa [Rattus norvegicus] | 27,1 | 4 |
| gi\|149062018 | mitochondrial ribosomal protein L11 [Rattus norvegicus] | 20,3 | 2 |
| gi\|71361655 | mitochondrial ribosomal protein L12 [Rattus norvegicus] | 31,2 | 3 |
| gi\|71361649 | mitochondrial ribosomal protein L19 [Rattus norvegicus] | 11,6 | 2 |
| gi\|67846004 | mitochondrial ribosomal protein L40 precursor [Rattus norvegicus] | 37,4 | 3 |
| gi\|169790975 | mitochondrial ribosomal protein S9 [Rattus norvegicus] | 16,4 | 2 |
| gi\|149042266 | moesin, isoform CRA_a [Rattus norvegicus] | 43,3 | 23 |
| gi\|158138498 | muscle glycogen phosphorylase [Rattus norvegicus] | 60,2 | 84 |
| gi\|197927186 | myofibrillogenesis regulator 1 isoform 3 [Rattus norvegicus] | 33,1 | 4 |
| gi\|6139055 | myo-inositol monophosphatase [Rattus norvegicus] | 14,4 | 3 |
| gi\|281306803 | myomesin 2 [Rattus norvegicus] | 44,6 | 43 |
| gi\|157820955 | myopalladin [Rattus norvegicus] | 10,9 | 4 |

| | | | |
|---|---|---|---|
| gi\|157824043 | myosin binding protein C, cardiac [Rattus norvegicus] | 62,5 | 121 |
| gi\|160961485 | myosin light chain kinase 3 [Rattus norvegicus] | 24,1 | 8 |
| gi\|211938461 | myosin, heavy chain 7B, cardiac muscle, beta [Rattus norvegicus] | 28 | 10 |
| gi\|149066032 | myosin, heavy polypeptide 9, non-muscle [Rattus norvegicus] | 21,5 | 5 |
| gi\|903892 | myotrophin [Rattus norvegicus] | 25,4 | 2 |
| gi\|149025875 | myozenin 2 (predicted), isoform CRA_a [Rattus norvegicus] | 38,6 | 4 |
| gi\|6978545 | Na+/K+ -ATPase alpha 2 subunit precursor [Rattus norvegicus] | 25,2 | 16 |
| gi\|187469748 | Naca protein [Rattus norvegicus] | 32,1 | 3 |
| gi\|171847415 | N-acetylneuraminic acid synthase [Rattus norvegicus] | 16,2 | 2 |
| gi\|53733394 | NAD(P)H dehydrogenase, quinone 1 [Rattus norvegicus] | 14,6 | 3 |
| gi\|6981260 | NADH dehydrogenase (ubiquinone) 1 alpha subcomplex  5 [Rattus norvegicus] | 58,6 | 5 |
| gi\|157818537 | NADH dehydrogenase (ubiquinone) 1 alpha subcomplex, 1 [Rattus norvegicus] | 23,3 | 2 |
| gi\|164565371 | NADH dehydrogenase (ubiquinone) 1 alpha subcomplex, 12 [Rattus norvegicus] | 64,8 | 14 |
| gi\|157817861 | NADH dehydrogenase (ubiquinone) 1 alpha subcomplex, 2 [Rattus norvegicus] | 75,3 | 11 |
| gi\|189085365 | NADH dehydrogenase (ubiquinone) 1 alpha subcomplex, 4 [Rattus norvegicus] | 63,4 | 28 |
| gi\|194473636 | NADH dehydrogenase (ubiquinone) 1 alpha subcomplex, 6 [Rattus norvegicus] | 58,5 | 10 |
| gi\|149038913 | NADH dehydrogenase (ubiquinone) 1 alpha subcomplex, 8 [Rattus norvegicus] | 64,5 | 14 |
| gi\|198278533 | NADH dehydrogenase (ubiquinone) 1 alpha subcomplex, 9 [Rattus norvegicus] | 43,8 | 13 |
| gi\|157817556 | NADH dehydrogenase (ubiquinone) 1 alpha subcomplex, assembly factor 1 [Rattus norvegicus] | 22,3 | 6 |
| gi\|157824071 | NADH dehydrogenase (ubiquinone) 1 beta subcomplex 3 [Rattus norvegicus] | 56,6 | 5 |
| gi\|82617686 | NADH dehydrogenase (ubiquinone) 1 beta subcomplex 4 [Rattus norvegicus] | 45 | 12 |
| gi\|187469737 | NADH dehydrogenase (ubiquinone) 1 beta subcomplex 8 [Rattus norvegicus] | 48,9 | 16 |
| gi\|157822175 | NADH dehydrogenase (ubiquinone) 1 beta subcomplex, 10 [Rattus norvegicus] | 59,1 | 29 |
| gi\|157822851 | NADH dehydrogenase (ubiquinone) 1 beta subcomplex, 11 [Rattus norvegicus] | 57 | 7 |
| gi\|219277692 | NADH dehydrogenase (ubiquinone) 1 beta subcomplex, 2 [Rattus norvegicus] | 32,4 | 3 |
| gi\|157823387 | NADH dehydrogenase (ubiquinone) 1 beta subcomplex, 5 [Rattus norvegicus] | 33,9 | 13 |
| gi\|157820465 | NADH dehydrogenase (ubiquinone) 1 beta subcomplex, 6 [Rattus norvegicus] | 51,6 | 4 |
| gi\|157823197 | NADH dehydrogenase (ubiquinone) 1 beta subcomplex, 7 [Rattus norvegicus] | 67,2 | 17 |
| gi\|197245756 | NADH dehydrogenase (ubiquinone) 1 beta subcomplex, 9 [Rattus norvegicus] | 63,7 | 16 |

| | | | |
|---|---|---|---|
| gi\|157820787 | NADH dehydrogenase (ubiquinone) 1, alpha/beta subcomplex, 1 [Rattus norvegicus] | 41 | 11 |
| gi\|57164133 | NADH dehydrogenase (ubiquinone) 1, subcomplex unknown, 2 [Rattus norvegicus] | 59,2 | 12 |
| gi\|149022594 | NADH dehydrogenase (ubiquinone) Fe-S protein 3 (predicted), isoform CRA_b [Rattus norvegicus] | 60,2 | 25 |
| gi\|72086149 | NADH dehydrogenase (ubiquinone) Fe-S protein 5b [Rattus norvegicus] | 60,4 | 9 |
| gi\|56606108 | NADH dehydrogenase (ubiquinone) Fe-S protein 7 [Rattus norvegicus] | 28,9 | 13 |
| gi\|54145379 | NADH dehydrogenase subunit 3 [Rattus norvegicus] | 31,3 | 3 |
| gi\|55741424 | NADH dehydrogenase ubiquinone flavoprotein 1 precursor [Rattus norvegicus] | 44 | 36 |
| gi\|74353679 | Ncam1 protein [Rattus norvegicus] | 11,8 | 2 |
| gi\|163915993 | Ndufa7 protein [Rattus norvegicus] | 49,1 | 9 |
| gi\|165971299 | Ndufs8 protein [Rattus norvegicus] | 50,5 | 13 |
| gi\|149047823 | nebulin (predicted) [Rattus norvegicus] | 15,6 | 3 |
| gi\|166091482 | nebulin-related anchoring protein isoform S [Rattus norvegicus] | 21,9 | 5 |
| gi\|987087 | neurolysin; neurotensin-degrading neutral metalloendopeptidase [Rattus norvegicus] | 19,2 | 3 |
| gi\|157822919 | neutral alpha-glucosidase AB [Rattus norvegicus] | 11,5 | 2 |
| gi\|76880449 | NFS1 nitrogen fixation 1 homolog [Rattus norvegicus] | 24 | 3 |
| gi\|282154799 | NFU1 iron-sulfur cluster scaffold homolog precursor [Rattus norvegicus] | 24,1 | 3 |
| gi\|157818439 | NHL repeat containing 2 [Rattus norvegicus] | 5,7 | 2 |
| gi\|77628000 | nitrilase family, member 2 [Rattus norvegicus] | 43,1 | 9 |
| gi\|157818303 | NMDA receptor regulated 1 [Rattus norvegicus] | 17,1 | 2 |
| gi\|157816949 | nodal modulator 1 [Rattus norvegicus] | 11,5 | 2 |
| gi\|6822247 | Nogo-A protein [Rattus norvegicus] | 19,4 | 7 |
| gi\|4210985 | non-muscle alpha-actinin 1 [Rattus norvegicus] | 41 | 25 |
| gi\|7839520 | NPW16 [Rattus norvegicus] | 35,9 | 4 |
| gi\|48734832 | NSFL1 (p97) cofactor (p47) [Rattus norvegicus] | 40,8 | 6 |
| gi\|8394272 | nuclear distribution gene C homolog [Rattus norvegicus] | 31 | 4 |
| gi\|74353675 | Nuclear protein localization 4 homolog (S. cerevisiae) [Rattus norvegicus] | 13,2 | 4 |
| gi\|50582542 | nucleolar protein 3 [Rattus norvegicus] | 23,5 | 3 |
| gi\|55250726 | Nucleolin [Rattus norvegicus] | 33,9 | 6 |
| gi\|55926145 | nucleoside diphosphate kinase B [Rattus norvegicus] | 67,8 | 18 |

| | | | |
|---|---|---|---|
| gi\|157823211 | nudix (nucleoside diphosphate linked moiety X)-type motif 15 [Rattus norvegicus] | 19,4 | 2 |
| gi\|189083707 | nudix-type motif 13 [Rattus norvegicus] | 19 | 2 |
| gi\|76677911 | Obg-like ATPase 1 [Rattus norvegicus] | 25 | 3 |
| gi\|202959 | ORF2 [Rattus norvegicus] | 49,7 | 9 |
| gi\|38181818 | Ornithine aminotransferase (gyrate atrophy) [Rattus norvegicus] | 27,3 | 6 |
| gi\|157824158 | oxidative-stress responsive 1 [Rattus norvegicus] | 17,8 | 3 |
| gi\|157821663 | oxysterol binding protein [Rattus norvegicus] | 18,3 | 3 |
| gi\|538153 | p115 [Rattus norvegicus] | 22,2 | 6 |
| gi\|1916984 | p38 mitogen activated protein kinase [Rattus norvegicus] | 14,2 | 3 |
| gi\|47938964 | Palm protein [Rattus norvegicus] | 20,4 | 2 |
| gi\|149024784 | pantothenate kinase 4 [Rattus norvegicus] | 20,6 | 5 |
| gi\|198041989 | parvin, beta [Rattus norvegicus] | 24,1 | 4 |
| gi\|55716041 | Pcx protein [Rattus norvegicus] | 22,3 | 11 |
| gi\|50925459 | Pcyt2 protein [Rattus norvegicus] | 24,4 | 4 |
| gi\|60688224 | Pdhx protein [Rattus norvegicus] | 44,5 | 14 |
| gi\|8393153 | PDZ and LIM domain 1 [Rattus norvegicus] | 35,5 | 11 |
| gi\|149054556 | peptidase (prosome, macropain) 26S subunit, ATPase 5, isoform CRA_b [Rattus norvegicus] | 33,7 | 7 |
| gi\|56268806 | Peptidylprolyl isomerase F (cyclophilin F) [Rattus norvegicus] | 54,4 | 14 |
| gi\|34849738 | Peroxiredoxin 2 [Rattus norvegicus] | 50 | 12 |
| gi\|149040547 | peroxiredoxin 3 [Rattus norvegicus] | 38,9 | 16 |
| gi\|51261175 | Peroxiredoxin 5 [Rattus norvegicus] | 51,2 | 13 |
| gi\|6539658 | peroxisomal phytanoyl-CoA hydroxylase [Rattus norvegicus] | 18,9 | 2 |
| gi\|149015966 | peroxisomal trans-2-enoyl-CoA reductase, isoform CRA_a [Rattus norvegicus] | 32 | 2 |
| gi\|41350889 | Pgam1 protein [Rattus norvegicus] | 62,6 | 22 |
| gi\|118764083 | Pgm1 protein [Rattus norvegicus] | 52,3 | 36 |
| gi\|169642489 | Pgm2 protein [Rattus norvegicus] | 17,3 | 2 |
| gi\|51948478 | phenylalanyl-tRNA synthetase, beta subunit [Rattus norvegicus] | 18,3 | 3 |
| gi\|71043634 | phosducin-like 3 [Rattus norvegicus] | 19,6 | 2 |
| gi\|8393910 | phosphatidylethanolamine binding protein [Rattus norvegicus] | 81,8 | 25 |

| | | | |
|---|---|---|---|
| gi\|829055 | phosphatidylinositol transfer protein [Rattus norvegicus] | 25,5 | 2 |
| gi\|8393962 | phosphatidylinositol transfer protein [Rattus norvegicus] | 20,3 | 4 |
| gi\|6981352 | phosphofructokinase, liver [Rattus norvegicus] | 12,2 | 4 |
| gi\|56585024 | Phosphoglycerate kinase 1 [Rattus norvegicus] | 71,7 | 42 |
| gi\|8393948 | phosphoglycerate mutase 2 [Rattus norvegicus] | 56,5 | 44 |
| gi\|281332119 | phosphoglycolate phosphatase [Rattus norvegicus] | 13,7 | 2 |
| gi\|164663846 | phosphohistidine phosphatase 1 [Rattus norvegicus] | 66,9 | 7 |
| gi\|158341684 | phospholipase A2, activating protein [Rattus norvegicus] | 22,9 | 7 |
| gi\|8393981 | phospholipase C, delta 1 [Rattus norvegicus] | 16,4 | 5 |
| gi\|197246445 | Phosphomannomutase 2 [Rattus norvegicus] | 33,9 | 6 |
| gi\|56541198 | Phosphoribosyl pyrophosphate amidotransferase [Rattus norvegicus] | 11,2 | 2 |
| gi\|8394053 | phosphoribosyl pyrophosphate synthetase 1 [Rattus norvegicus] | 17,6 | 2 |
| gi\|61889077 | phosphotriesterase related [Rattus norvegicus] | 22,9 | 3 |
| gi\|71679761 | Picalm protein [Rattus norvegicus] | 10,1 | 2 |
| gi\|157819139 | pitrilysin metallopeptidase 1 [Rattus norvegicus] | 25,2 | 9 |
| gi\|215276950 | plakophilin 2 [Rattus norvegicus] | 23,4 | 3 |
| gi\|60688649 | Plasminogen [Rattus norvegicus] | 17,7 | 7 |
| gi\|40849896 | plectin 6 [Rattus norvegicus] | 25,3 | 4 |
| gi\|52789215 | Poly(A) binding protein, cytoplasmic 1 [Rattus norvegicus] | 23 | 4 |
| gi\|157786694 | polymerase I and transcript release factor [Rattus norvegicus] | 53,8 | 24 |
| gi\|215277019 | polyribonucleotide nucleotidyltransferase 1 [Rattus norvegicus] | 15,3 | 5 |
| gi\|71122474 | Ppa1 protein [Rattus norvegicus] | 26,9 | 3 |
| gi\|38541053 | Ppib protein [Rattus norvegicus] | 52,8 | 7 |
| gi\|38303831 | Ppm1b protein [Rattus norvegicus] | 22,2 | 3 |
| gi\|56072 | precursor polypeptide (AA -29 to 261) [Rattus norvegicus] | 61,4 | 35 |
| gi\|109511985 | PREDICTED: hypothetical protein [Rattus norvegicus] | 27,3 | 8 |
| gi\|109461422 | mitochondrial precursor (Branched-chain alpha-keto acid dehydrogenase E1 component alpha chain) (BCKDH E1-alpha) [Rattus norvegicus] | 40 | 14 |
| gi\|109461574 | PREDICTED: similar to 40S ribosomal protein S16 [Rattus norvegicus] | 23 | 2 |
| gi\|109496275 | PREDICTED: similar to 60S ribosomal protein L17 (L23) (Amino acid starvation-induced protein) (ASI) [Rattus norvegicus] | 27 | 2 |

| | | | |
|---|---|---|---|
| gi\|62638415 | PREDICTED: similar to 60S ribosomal protein L26 (Silica-induced gene 20 protein) (SIG-20) [Rattus norvegicus] | 20 | 2 |
| gi\|109490334 | PREDICTED: similar to 60S ribosomal protein L3-like [Rattus norvegicus] | 14,7 | 3 |
| gi\|34873230 | PREDICTED: similar to abhydrolase domain containing 11 [Rattus norvegicus] | 18,6 | 3 |
| gi\|109484416 | PREDICTED: similar to Acyl-CoA dehydrogenase family member 8, mitochondrial precursor (ACAD-8) (Isobutyryl-CoA dehydrogenase) [Rattus norvegicus] | 32,9 | 10 |
| gi\|62646885 | PREDICTED: similar to adenylate kinase 2 isoform b [Rattus norvegicus] | 41 | 14 |
| gi\|109463545 | PREDICTED: similar to AHNAK nucleoprotein isoform 1 isoform 1 [Rattus norvegicus] | 51,7 | 33 |
| gi\|109463553 | PREDICTED: similar to AHNAK nucleoprotein isoform 1 isoform 3 [Rattus norvegicus] | 49,6 | 36 |
| gi\|62664437 | PREDICTED: similar to aldehyde dehydrogenase family 7, member A1 [Rattus norvegicus] | 24,9 | 9 |
| gi\|109496584 | PREDICTED: similar to ATP synthase, H+ transporting, mitochondrial F0 complex, subunit f, isoform 2 [Rattus norvegicus] | 34,9 | 5 |
| gi\|109460528 | PREDICTED: similar to Band 4.1-like protein 2 (Generally expressed protein 4.1) (4.1G) [Rattus norvegicus] | 17,8 | 5 |
| gi\|109508252 | PREDICTED: similar to C50H11.1 [Rattus norvegicus] | 18,3 | 4 |
| gi\|62646841 | (Mitochondrial aspartate glutamate carrier 2) (Solute carrier family 25 member 13) (Citrin) [Rattus norvegicus] | 35,4 | 17 |
| gi\|109465895 | PREDICTED: similar to cardiomyopathy associated 5 [Rattus norvegicus] | 16,2 | 3 |
| gi\|109512256 | PREDICTED: similar to cation-dependent mannose-6-phosphate receptor [Rattus norvegicus] | 16,1 | 2 |
| gi\|109467679 | PREDICTED: similar to CG1458-PA [Rattus norvegicus] | 32,3 | 3 |
| gi\|109497479 | PREDICTED: similar to CG2453-PA [Rattus norvegicus] | 22,3 | 4 |
| gi\|109511686 | PREDICTED: similar to Cytochrome c-type heme lyase (CCHL) (Holocytochrome c-type synthase) [Rattus norvegicus] | 37,2 | 8 |
| gi\|109506992 | PREDICTED: similar to desmoglein 2 [Rattus norvegicus] | 14,8 | 4 |
| gi\|109506988 | PREDICTED: similar to Desmoglein-1 alpha precursor (Dsg1-alpha) (Desmoglein-1) (Desmosomal glycoprotein I) (DG1) (DGI) [Rattus norvegicus] | 5,4 | 2 |
| gi\|109505631 | PREDICTED: similar to desmoplakin isoform I isoform 2 [Rattus norvegicus] | 28,7 | 10 |
| gi\|109484674 | PREDICTED: similar to dihydrolipoamide S-acetyltransferase (E2 component of pyruvate dehydrogenase complex) [Rattus norvegicus] | 44,4 | 32 |
| gi\|109504649 | PREDICTED: similar to dimerization cofactor of hepatocyte nuclear factor 1 (HNF1) from muscle [Rattus norvegicus] | 54,4 | 2 |
| gi\|109487346 | PREDICTED: similar to DnaJ (Hsp40) homolog, subfamily B, member 2 isoform b isoform 9 [Rattus norvegicus] | 28,7 | 2 |
| gi\|62657092 | PREDICTED: similar to Dual specificity protein phosphatase 3 (T-DSP11) [Rattus norvegicus] | 21 | 4 |
| gi\|62638223 | PREDICTED: similar to dynactin 3 [Rattus norvegicus] | 24,7 | 2 |
| gi\|34865933 | PREDICTED: similar to Dystroglycan precursor (Dystrophin-associated glycoprotein 1) [Rattus norvegicus] | 14,9 | 7 |
| gi\|109511865 | PREDICTED: similar to dystrophin, muscular dystrophy [Rattus norvegicus] | 26,9 | 21 |
| gi\|109482048 | PREDICTED: similar to Elongation factor Ts, mitochondrial precursor (EF-Ts) (EF-TsMt) (2A3-2) isoform 2 [Rattus norvegicus] | 13,3 | 3 |
| gi\|62658155 | PREDICTED: similar to eukaryotic translation initiation factor 4, gamma 1 isoform a [Rattus norvegicus] | 20,9 | 9 |

| | | | |
|---|---|---|---|
| gi\|109501906 | PREDICTED: similar to F11C1.5a [Rattus norvegicus] | 23,8 | 16 |
| gi\|109472679 | isomerase) (PPIase) (Rotamase) (p59 protein) (HSP-binding immunophilin) (HBI) (FKBP52 protein) (52 kDa FK506-binding protein) (FKBP59) [Rattus norvegicus] | 28,8 | 3 |
| gi\|62657298 | PREDICTED: similar to glyceraldehyde-3-phosphate dehydrogenase [Rattus norvegicus] | 54,7 | 33 |
| gi\|109485458 | PREDICTED: similar to glycerol-3-phosphate dehydrogenase 1-like [Rattus norvegicus] | 27,4 | 5 |
| gi\|109461932 | PREDICTED: similar to glycogen synthase 1, muscle isoform 2 [Rattus norvegicus] | 30,8 | 8 |
| gi\|109473554 | PREDICTED: similar to glycyl-tRNA synthetase [Rattus norvegicus] | 16 | 4 |
| gi\|109495090 | PREDICTED: similar to High mobility group protein 1 (HMG-1) (High mobility group protein B1) (Amphoterin) (Heparin-binding protein p30) [Rattus norvegicus] | 25 | 4 |
| gi\|62638360 | PREDICTED: similar to High mobility group protein 2 (HMG-2) [Rattus norvegicus] | 23 | 2 |
| gi\|109490737 | monophosphoramidase) (Protein kinase C inhibitor 1) (Protein kinase C-interacting protein 1) (PKCI-1) [Rattus norvegicus] | 24,4 | 3 |
| gi\|34853298 | PREDICTED: similar to histidine triad protein 4 [Rattus norvegicus] | 38,3 | 2 |
| gi\|109505801 | PREDICTED: similar to Histone H1.2 (H1 VAR.1) (H1c) [Rattus norvegicus] | 21,7 | 3 |
| gi\|109492192 | PREDICTED: similar to Immature colon carcinoma transcript 1 protein precursor [Rattus norvegicus] | 30,6 | 2 |
| gi\|62644491 | binding protein 1) (Guanine nucleotide-binding protein 1) (HuGBP-1) [Rattus norvegicus] | 25,9 | 8 |
| gi\|109481333 | PREDICTED: similar to keratin Kb40 [Rattus norvegicus] | 17,9 | 3 |
| gi\|109501541 | PREDICTED: similar to kinectin 1 [Rattus norvegicus] | 22,9 | 4 |
| gi\|109460394 | PREDICTED: similar to Laminin alpha-2 chain precursor (Laminin M chain) (Merosin heavy chain) [Rattus norvegicus] | 15,1 | 9 |
| gi\|109500591 | PREDICTED: similar to LIM domain only protein 7 [Rattus norvegicus] | 26,5 | 4 |
| gi\|27663300 | mitochondrial precursor (MCT) (Mitochondrial malonyltransferase) [Rattus norvegicus] | 14 | 2 |
| gi\|109513120 | PREDICTED: similar to mannose-6-phosphate receptor binding protein 1 [Rattus norvegicus] | 22,4 | 4 |
| gi\|109497496 | PREDICTED: similar to methylmalonic aciduria (cobalamin deficiency) type B homolog [Rattus norvegicus] | 19,8 | 2 |
| gi\|62654757 | PREDICTED: similar to Methylmalonyl-CoA mutase, mitochondrial precursor (MCM) (Methylmalonyl-CoA isomerase) [Rattus norvegicus] | 29,9 | 13 |
| gi\|109498993 | PREDICTED: similar to microsomal glutathione S-transferase 3 [Rattus norvegicus] | 29 | 3 |
| gi\|34854147 | PREDICTED: similar to Mimitin, mitochondrial precursor (Myc-induced mitochondrial protein) (MMTN) [Rattus norvegicus] | 24,7 | 2 |
| gi\|109481425 | PREDICTED: similar to MIR-interacting saposin-like protein precursor (Transmembrane protein 4) (Putative secreted protein ZSIG9) [Rattus norvegicus] | 31,2 | 2 |
| gi\|109489724 | PREDICTED: similar to mitochondria-associated granulocyte macrophage CSF signaling molecule [Rattus norvegicus] | 22 | 5 |
| gi\|109462235 | PREDICTED: similar to myocyte induction differentiation originator [Rattus norvegicus] | 15,3 | 2 |
| gi\|109487636 | PREDICTED: similar to Myomesin-1 (Skelemin) isoform 1 [Rattus norvegicus] | 25 | 26 |
| gi\|109506129 | PREDICTED: similar to Nebulette (Actin-binding Z-disk protein) [Rattus norvegicus] | 33,8 | 15 |
| gi\|109505096 | PREDICTED: similar to Nidogen-1 precursor (Entactin) [Rattus norvegicus] | 9,6 | 5 |

| | | | |
|---|---|---|---|
| gi\|109477262 | PREDICTED: similar to NonO/p54nrb homolog [Rattus norvegicus] | 30,9 | 6 |
| gi\|109512451 | PREDICTED: similar to Nucleophosmin (NPM) (Nucleolar phosphoprotein B23) (Numatrin) (Nucleolar protein NO38) [Rattus norvegicus] | 28,8 | 2 |
| gi\|109463329 | PREDICTED: similar to nudix-type motif 8 [Rattus norvegicus] | 18,8 | 2 |
| gi\|109490823 | PREDICTED: similar to obscurin, cytoskeletal calmodulin and titin-interacting RhoGEF [Rattus norvegicus] | 16,2 | 4 |
| gi\|62655561 | PREDICTED: similar to peptidylprolyl isomerase D [Rattus norvegicus] | 26 | 6 |
| gi\|109463725 | PREDICTED: similar to phosphoglucomutase 5 [Rattus norvegicus] | 25,9 | 8 |
| gi\|109502891 | PREDICTED: similar to Potassium channel tetramerisation domain containing protein 12 (Pfetin) (Predominantly fetal expressed T1 domain) [Rattus norvegicus] | 19,2 | 2 |
| gi\|62651145 | PREDICTED: similar to Protein C14orf159, mitochondrial precursor [Rattus norvegicus] | 20,9 | 4 |
| gi\|109483709 | PREDICTED: similar to Protein C6orf142 homolog [Rattus norvegicus] | 21,4 | 4 |
| gi\|109476306 | PREDICTED: similar to Protein KIAA1045 [Rattus norvegicus] | 25,1 | 3 |
| gi\|109473811 | PREDICTED: similar to RAB11 family interacting protein 5 (class I) isoform 1 [Rattus norvegicus] | 11,1 | 3 |
| gi\|109502869 | PREDICTED: similar to RAN binding protein 5 [Rattus norvegicus] | 10,7 | 7 |
| gi\|109500414 | PREDICTED: similar to rap2 interacting protein x [Rattus norvegicus] | 21,6 | 3 |
| gi\|62654783 | PREDICTED: similar to ribosomal protein L10a [Rattus norvegicus] | 25,8 | 2 |
| gi\|34876783 | PREDICTED: similar to ribosomal protein L30 [Rattus norvegicus] | 19,5 | 2 |
| gi\|62655953 | PREDICTED: similar to ribosomal protein S10 [Rattus norvegicus] | 36,4 | 4 |
| gi\|109470919 | PREDICTED: similar to Ribosome-binding protein 1 (Ribosome receptor protein) (mRRp) isoform 2 [Rattus norvegicus] | 22 | 4 |
| gi\|34867677 | PREDICTED: similar to Serine protease inhibitor A3M precursor (Serine protease inhibitor 2.4) (SPI-2.4) [Rattus norvegicus] | 18,6 | 8 |
| gi\|109512218 | PREDICTED: similar to SH3 domain-binding glutamic acid-rich-like protein [Rattus norvegicus] | 14,6 | 3 |
| gi\|34854800 | PREDICTED: similar to solute carrier family 25 (mitochondrial carrier, Aralar), member 12 [Rattus norvegicus] | 40,2 | 3 |
| gi\|109468291 | PREDICTED: similar to solute carrier family 25 (mitochondrial carrier, Aralar), member 12 [Rattus norvegicus] | 24,6 | 5 |
| gi\|109463865 | PREDICTED: similar to sorbin and SH3 domain containing 1 isoform 3 [Rattus norvegicus] | 22,3 | 11 |
| gi\|109486883 | PREDICTED: similar to stromal membrane-associated protein 1 [Rattus norvegicus] | 5,1 | 2 |
| gi\|109480098 | PREDICTED: similar to SWI/SNF-related matrix-associated actin-dependent regulator of chromatin c2 isoform b isoform 2 [Rattus norvegicus] | 10,4 | 2 |
| gi\|62653851 | PREDICTED: similar to talin 2 [Rattus norvegicus] | 15,6 | 9 |
| gi\|109470195 | PREDICTED: similar to tankyrase 1-binding protein of 182 kDa [Rattus norvegicus] | 11,7 | 2 |
| gi\|109487302 | PREDICTED: similar to tensin [Rattus norvegicus] | 14,4 | 11 |
| gi\|109495987 | PREDICTED: similar to tescalcin [Rattus norvegicus] | 22,9 | 2 |
| gi\|109468337 | PREDICTED: similar to titin isoform N2-A [Rattus norvegicus] | 19,9 | 18 |

| | | | |
|---|---|---|---|
| gi\|109470142 | PREDICTED: similar to titin isoform N2-B [Rattus norvegicus] | 19 | 73 |
| gi\|109462731 | PREDICTED: similar to tRNA nucleotidyl transferase, CCA-adding, 1 isoform 3 [Rattus norvegicus] | 26,5 | 2 |
| gi\|109474148 | PREDICTED: similar to Tubulin alpha-8 chain (Alpha-tubulin 8) [Rattus norvegicus] | 49,4 | 22 |
| gi\|109476934 | thiolesterase 24) (Ubiquitin-specific-processing protease 24) (Deubiquitinating enzyme 24) [Rattus norvegicus] | 11,2 | 2 |
| gi\|62659702 | PREDICTED: similar to UDP-N-acetylhexosamine pyrophosphorylase [Rattus norvegicus] | 18,8 | 4 |
| gi\|157822777 | prefoldin subunit 1 [Rattus norvegicus] | 48,4 | 2 |
| gi\|55882 | preprocathepsin D [Rattus norvegicus] | 19,9 | 5 |
| gi\|149037631 | procollagen, type VI, alpha 3 (predicted), isoform CRA_c [Rattus norvegicus] | 15,1 | 5 |
| gi\|1628436 | profilin [Rattus norvegicus] | 42,9 | 7 |
| gi\|157819939 | programmed cell death 5 [Rattus norvegicus] | 60,8 | 3 |
| gi\|210032180 | programmed cell death 6 interacting protein [Rattus norvegicus] | 19,9 | 7 |
| gi\|66911717 | Prohibitin [Rattus norvegicus] | 55,2 | 23 |
| gi\|209413776 | proline dehydrogenase [Rattus norvegicus] | 13,5 | 2 |
| gi\|157823503 | proline synthetase co-transcribed homolog [Rattus norvegicus] | 12,7 | 2 |
| gi\|6981424 | prosaposin precursor [Rattus norvegicus] | 22,4 | 4 |
| gi\|157822395 | prostaglandin E synthase 2 [Rattus norvegicus] | 31 | 5 |
| gi\|38197361 | Prostaglandin I2 (prostacyclin) synthase [Rattus norvegicus] | 8 | 2 |
| gi\|61098214 | protease (prosome, macropain) 28 subunit, alpha [Rattus norvegicus] | 35,7 | 8 |
| gi\|6981420 | protease, serine, 2 precursor [Rattus norvegicus] | 28,1 | 36 |
| gi\|38181888 | Proteasome (prosome, macropain) 26S subunit, ATPase 2 [Rattus norvegicus] | 29,3 | 7 |
| gi\|149022610 | proteasome (prosome, macropain) 26S subunit, ATPase 3, isoform CRA_c [Rattus norvegicus] | 27,3 | 4 |
| gi\|38970025 | Proteasome (prosome, macropain) 26S subunit, ATPase, 4 [Rattus norvegicus] | 18,9 | 4 |
| gi\|157820107 | proteasome (prosome, macropain) 26S subunit, non-ATPase, 11 [Rattus norvegicus] | 22,5 | 5 |
| gi\|72255509 | proteasome (prosome, macropain) 26S subunit, non-ATPase, 2 [Rattus norvegicus] | 21,4 | 5 |
| gi\|56605666 | proteasome (prosome, macropain) 26S subunit, non-ATPase, 3 [Rattus norvegicus] | 33 | 7 |
| gi\|149038941 | proteasome (prosome, macropain) 26S subunit, non-ATPase, 5 (predicted), isoform CRA_a [Rattus norvegicus] | 19,6 | 3 |
| gi\|38014563 | Proteasome (prosome, macropain) subunit, alpha type 5 [Rattus norvegicus] | 20,8 | 5 |
| gi\|149047897 | proteasome (prosome, macropain) subunit, beta type 7 [Rattus norvegicus] | 15,6 | 2 |
| gi\|54400716 | proteasome 26S non-ATPase subunit 12 [Rattus norvegicus] | 30,3 | 4 |

| | | | |
|---|---|---|---|
| gi\|8394091 | proteasome activator subunit 2 [Rattus norvegicus] | 28,2 | 3 |
| gi\|8394060 | proteasome alpha 1 subunit [Rattus norvegicus] | 34,6 | 6 |
| gi\|8394063 | proteasome alpha 2 subunit [Rattus norvegicus] | 10,7 | 2 |
| gi\|8394066 | proteasome alpha 3 subunit [Rattus norvegicus] | 29,4 | 4 |
| gi\|8394069 | proteasome alpha 4 subunit [Rattus norvegicus] | 18,8 | 3 |
| gi\|8394076 | proteasome alpha 6 subunit [Rattus norvegicus] | 19,9 | 2 |
| gi\|56550075 | proteasome alpha 7 subunit [Rattus norvegicus] | 41,5 | 6 |
| gi\|56998 | proteasome subunit RC5 [Rattus norvegicus] | 32,5 | 5 |
| gi\|38454206 | proteasome, 26S, non-ATPase regulatory subunit 6 [Rattus norvegicus] | 26,5 | 4 |
| gi\|76880465 | protein kinase C and casein kinase substrate in neurons 2 [Rattus norvegicus] | 30,9 | 11 |
| gi\|71681475 | Protein kinase C and casein kinase substrate in neurons 3 [Rattus norvegicus] | 40,8 | 7 |
| gi\|149020437 | protein kinase C substrate 80K-H (predicted), isoform CRA_a [Rattus norvegicus] | 17,1 | 4 |
| gi\|206150 | protein kinase type II regulatory subunit (, EC 2.7.1.37) [Rattus norvegicus] | 36 | 5 |
| gi\|8394036 | protein phospatase 3, regulatory subunit B, alpha isoform,type 1 [Rattus norvegicus] | 22,9 | 2 |
| gi\|149061963 | protein phosphatase 1, catalytic subunit, alpha isoform, isoform CRA_b [Rattus norvegicus] | 22,7 | 2 |
| gi\|6981388 | protein phosphatase 1, catalytic subunit, beta [Rattus norvegicus] | 33 | 10 |
| gi\|157821043 | protein phosphatase 1, regulatory (inhibitor) subunit 12B [Rattus norvegicus] | 33,7 | 8 |
| gi\|56270302 | Protein phosphatase 1, regulatory (inhibitor) subunit 14c [Rattus norvegicus] | 29,3 | 2 |
| gi\|62089492 | Protein phosphatase 1, regulatory (inhibitor) subunit 2 [Rattus norvegicus] | 15,6 | 2 |
| gi\|157822901 | protein phosphatase 1, regulatory (inhibitor) subunit 3A [Rattus norvegicus] | 15,1 | 4 |
| gi\|8394012 | protein phosphatase 1A [Rattus norvegicus] | 18,6 | 4 |
| gi\|157823133 | protein phosphatase 1K (PP2C domain containing) [Rattus norvegicus] | 17,5 | 2 |
| gi\|187469721 | Protein phosphatase 2A activator, regulatory subunit 4 [Rattus norvegicus] | 25,1 | 4 |
| gi\|8394018 | protein phosphatase 2a, catalytic subunit, alpha isoform [Rattus norvegicus] | 25,2 | 5 |
| gi\|149045445 | protein phosphatase 2C, magnesium dependent, catalytic subunit, isoform CRA_b [Rattus norvegicus] | 32,2 | 8 |
| gi\|663080 | protein phosphatase T (PPT) [Rattus norvegicus] | 18,4 | 2 |
| gi\|741804 | protein RAKc | 47,4 | 10 |
| gi\|155369684 | protein tyrosine phosphatase-like (proline instead of catalytic arginine), member a [Rattus norvegicus] | 13,7 | 3 |
| gi\|149039523 | protein-L-isoaspartate (D-aspartate) O-methyltransferase 1, isoform CRA_d [Rattus norvegicus] | 44,2 | 5 |

| | | | |
|---|---|---|---|
| gi\|56970 | prothrombin precursor [Rattus norvegicus] | 28,9 | 6 |
| gi\|62201921 | Prothymosin alpha [Rattus norvegicus] | 35,7 | 5 |
| gi\|81294202 | Psmc6 protein [Rattus norvegicus] | 27 | 3 |
| gi\|209529636 | pyrophosphatase (inorganic) 2 [Rattus norvegicus] | 38,5 | 11 |
| gi\|71051030 | Pyruvate dehydrogenase (lipoamide) alpha 1 [Rattus norvegicus] | 60,3 | 33 |
| gi\|56090293 | pyruvate dehydrogenase (lipoamide) beta precursor [Rattus norvegicus] | 58,8 | 52 |
| gi\|59709473 | pyruvate dehydrogenase kinase 1 precursor [Rattus norvegicus] | 56,7 | 15 |
| gi\|694003 | pyruvate dehydrogenase kinase 2 subunit p45 [Rattus norvegicus] | 43,5 | 11 |
| gi\|157818167 | pyruvate dehydrogenase phosphatase regulatory subunit [Rattus norvegicus] | 13,4 | 4 |
| gi\|61889071 | RAB10, member RAS oncogene family [Rattus norvegicus] | 35,5 | 8 |
| gi\|61098195 | RAB3A, member RAS oncogene family [Rattus norvegicus] | 20 | 4 |
| gi\|77404180 | RAB4A, member RAS oncogene family [Rattus norvegicus] | 16,5 | 4 |
| gi\|149029653 | RAB5B, member RAS oncogene family (predicted), isoform CRA_b [Rattus norvegicus] | 29,2 | 2 |
| gi\|165970759 | Rab5c protein [Rattus norvegicus] | 25 | 4 |
| gi\|70778952 | RAD23 homolog B [Rattus norvegicus] | 14,7 | 3 |
| gi\|56799432 | radixin [Rattus norvegicus] | 42,5 | 11 |
| gi\|157820113 | RAN binding protein 1 [Rattus norvegicus] | 31,5 | 2 |
| gi\|54114993 | RAP1A, member of RAS oncogene family precursor [Rattus norvegicus] | 45,1 | 6 |
| gi\|4079645 | RAREG-2.1 [Rattus norvegicus] | 27,3 | 5 |
| gi\|6981476 | Ras homolog enriched in brain precursor [Rattus norvegicus] | 14,7 | 2 |
| gi\|157819711 | ras homolog gene family, member T1 [Rattus norvegicus] | 12,4 | 2 |
| gi\|206555 | ras protein [Rattus norvegicus] | 37,7 | 6 |
| gi\|157821061 | Ras suppressor protein 1 [Rattus norvegicus] | 24,9 | 3 |
| gi\|54607147 | ras-related C3 botulinum toxin substrate 1 (rho family, small GTP binding protein Rac1) precursor [Rattus norvegicus] | 23,4 | 3 |
| gi\|149040843 | rCG20317, isoform CRA_a [Rattus norvegicus] | 26,4 | 7 |
| gi\|149025275 | rCG20659, isoform CRA_c [Rattus norvegicus] | 28,6 | 3 |
| gi\|149025373 | rCG20813 [Rattus norvegicus] | 29,8 | 5 |
| gi\|149016272 | rCG23940, isoform CRA_g [Rattus norvegicus] | 25,4 | 3 |
| gi\|149018394 | rCG25289 [Rattus norvegicus] | 14,1 | 3 |

| | | | |
|---|---|---|---|
| gi\|149018741 | rCG25673, isoform CRA_d [Rattus norvegicus] | 38,8 | 2 |
| gi\|149019103 | rCG25753, isoform CRA_b [Rattus norvegicus] | 18,9 | 2 |
| gi\|149019173 | rCG25937, isoform CRA_a [Rattus norvegicus] | 56 | 4 |
| gi\|149044000 | rCG27696 [Rattus norvegicus] | 22,3 | 3 |
| gi\|149044044 | rCG27771, isoform CRA_b [Rattus norvegicus] | 22,3 | 6 |
| gi\|149044009 | rCG27793, isoform CRA_c [Rattus norvegicus] | 21,4 | 3 |
| gi\|149026101 | rCG28661, isoform CRA_b [Rattus norvegicus] | 28,6 | 11 |
| gi\|149049470 | rCG29914, isoform CRA_b [Rattus norvegicus] | 69,2 | 38 |
| gi\|149055325 | rCG30560 [Rattus norvegicus] | 25 | 19 |
| gi\|149024343 | rCG30666, isoform CRA_a [Rattus norvegicus] | 15,9 | 5 |
| gi\|149023892 | rCG31143 [Rattus norvegicus] | 55,3 | 2 |
| gi\|149023879 | rCG31475, isoform CRA_b [Rattus norvegicus] | 26,4 | 7 |
| gi\|149037138 | rCG31964, isoform CRA_c [Rattus norvegicus] | 20,4 | 2 |
| gi\|149042824 | rCG32122, isoform CRA_a [Rattus norvegicus] | 27 | 2 |
| gi\|149042757 | rCG32197, isoform CRA_a [Rattus norvegicus] | 47 | 2 |
| gi\|149052642 | rCG33456, isoform CRA_c [Rattus norvegicus] | 54,2 | 45 |
| gi\|149054664 | rCG35339 [Rattus norvegicus] | 8,7 | 2 |
| gi\|149047665 | rCG35703, isoform CRA_b [Rattus norvegicus] | 27 | 2 |
| gi\|149019917 | rCG36507, isoform CRA_a [Rattus norvegicus] | 17,3 | 4 |
| gi\|149019916 | rCG36700 [Rattus norvegicus] | 18,8 | 6 |
| gi\|149035969 | rCG38845, isoform CRA_b [Rattus norvegicus] | 63,2 | 19 |
| gi\|149031313 | rCG38907, isoform CRA_a [Rattus norvegicus] | 11,7 | 2 |
| gi\|149068775 | rCG39700, isoform CRA_b [Rattus norvegicus] | 44,2 | 6 |
| gi\|149048476 | rCG41402, isoform CRA_c [Rattus norvegicus] | 24,1 | 2 |
| gi\|149048690 | rCG41429, isoform CRA_b [Rattus norvegicus] | 28,8 | 3 |
| gi\|149032791 | rCG41951, isoform CRA_a [Rattus norvegicus] | 64,7 | 12 |
| gi\|149034099 | rCG42329, isoform CRA_a [Rattus norvegicus] | 49,4 | 7 |
| gi\|149029681 | rCG42490, isoform CRA_e [Rattus norvegicus] | 38 | 4 |
| gi\|149029697 | rCG42519, isoform CRA_a [Rattus norvegicus] | 51,3 | 45 |

| | | | |
|---|---|---|---|
| gi\|149045167 | rCG43947 [Rattus norvegicus] | 14,9 | 3 |
| gi\|149039410 | rCG45400 [Rattus norvegicus] | 51,9 | 35 |
| gi\|149062344 | rCG47621, isoform CRA_b [Rattus norvegicus] | 38,7 | 2 |
| gi\|149062039 | rCG48149, isoform CRA_d [Rattus norvegicus] | 45,1 | 11 |
| gi\|149017065 | rCG49368, isoform CRA_a [Rattus norvegicus] | 23,8 | 6 |
| gi\|149042653 | rCG49803, isoform CRA_a [Rattus norvegicus] | 28,5 | 6 |
| gi\|149035501 | rCG50422, isoform CRA_a [Rattus norvegicus] | 25,7 | 2 |
| gi\|149031961 | rCG50520 [Rattus norvegicus] | 24,4 | 15 |
| gi\|149031880 | rCG50547, isoform CRA_b [Rattus norvegicus] | 34,3 | 5 |
| gi\|149031922 | rCG50739, isoform CRA_c [Rattus norvegicus] | 23,3 | 4 |
| gi\|149043237 | rCG50929 [Rattus norvegicus] | 39,2 | 3 |
| gi\|149056633 | rCG54023, isoform CRA_a [Rattus norvegicus] | 41,2 | 3 |
| gi\|149056475 | rCG54610, isoform CRA_a [Rattus norvegicus] | 29,2 | 5 |
| gi\|149045808 | rCG54790, isoform CRA_c [Rattus norvegicus] | 51 | 2 |
| gi\|149045751 | rCG55135, isoform CRA_a [Rattus norvegicus] | 25,7 | 23 |
| gi\|149031440 | rCG56755, isoform CRA_b [Rattus norvegicus] | 25,6 | 2 |
| gi\|149029807 | rCG56815, isoform CRA_b [Rattus norvegicus] | 45,6 | 2 |
| gi\|149035247 | rCG57156 [Rattus norvegicus] | 27,5 | 6 |
| gi\|149065952 | rCG60046 [Rattus norvegicus] | 13,5 | 3 |
| gi\|149043497 | rCG61099, isoform CRA_a [Rattus norvegicus] | 39,5 | 2 |
| gi\|149065303 | rCG63680 [Rattus norvegicus] | 42 | 2 |
| gi\|149058161 | RCSD domain containing 1 (predicted), isoform CRA_a [Rattus norvegicus] | 32,3 | 5 |
| gi\|270288782 | receptor accessory protein 5 [Rattus norvegicus] | 25,4 | 4 |
| gi\|461731 | RecName: Full=10 kDa heat shock protein, mitochondrial; AltName: Full=Hsp10; AltName: Full=10 kDa chaperonin; AltName: Full=CPN10 | 86,3 | 7 |
| gi\|61216932 | RecName: Full=14-3-3 protein epsilon; Short=14-3-3E; AltName: Full=Mitochondrial import stimulation factor L subunit; Short=MSF L | 73,7 | 38 |
| gi\|49065780 | RecName: Full=26S protease regulatory subunit 4; AltName: Full=P26s4; AltName: Full=Proteasome 26S subunit ATPase 1 | 25,7 | 3 |
| gi\|229891500 | Full=26S proteasome regulatory subunit S11; AltName: Full=26S proteasome regulatory subunit p40.5 | 25,3 | 5 |
| gi\|257096782 | RecName: Full=28S ribosomal protein S31, mitochondrial; Short=S31mt; Short=MRP-S31; Flags: Precursor | 17,8 | 3 |
| gi\|81884155 | RecName: Full=39S ribosomal protein L24, mitochondrial; Short=L24mt; Short=MRP-L24; Flags: Precursor | 28,2 | 2 |

| | | | |
|---|---|---|---|
| gi\|7387724 | hydroxyacyl-CoA dehydrogenase type II; AltName: Full=Type II HADH; AltName: Full=3-hydroxy-2-methylbutyryl-CoA dehydrogenase; AltName: Full=17-beta- | 49 | 15 |
| gi\|51338623 | RecName: Full=40S ribosomal protein S28 | 30,4 | 3 |
| gi\|730581 | RecName: Full=60S acidic ribosomal protein P0; AltName: Full=L10E | 27,1 | 3 |
| gi\|730529 | RecName: Full=60S ribosomal protein L13 | 23,2 | 2 |
| gi\|51338615 | RecName: Full=60S ribosomal protein L23a | 19,2 | 2 |
| gi\|51704206 | RecName: Full=60S ribosomal protein L7 | 28,9 | 2 |
| gi\|81885370 | RecName: Full=Acid ceramidase; Short=AC; AltName: Full=Acylsphingosine deacylase; AltName: Full=N-acylsphingosine amidohydrolase; Flags: Precursor | 21,1 | 6 |
| gi\|60391194 | RecName: Full=Aconitate hydratase, mitochondrial; Short=Aconitase; AltName: Full=Citrate hydro-lyase; Flags: Precursor | 72,4 | 179 |
| gi\|205686193 | RecName: Full=Actin-related protein 2/3 complex subunit 2; AltName: Full=Arp2/3 complex 34 kDa subunit; Short=p34-ARC | 36 | 6 |
| gi\|62899645 | RecName: Full=Actin-related protein 2; AltName: Full=Actin-like protein 2 | 38,6 | 8 |
| gi\|81907889 | RecName: Full=Actin-related protein 3; AltName: Full=Actin-like protein 3 | 23,2 | 4 |
| gi\|84028273 | AltName: Full=SUB1 homolog; AltName: Full=Positive cofactor 4; Short=PC4; AltName: Full=p14 | 25,2 | 2 |
| gi\|728810 | translocator 2; Short=ANT 2; AltName: Full=ADP,ATP carrier protein 2; AltName: Full=Solute carrier family 25 member 5 | 64,1 | 29 |
| gi\|81890516 | RecName: Full=ADP-ribosylation factor-like protein 8B | 22 | 2 |
| gi\|543793 | RecName: Full=Afamin; AltName: Full=Alpha-albumin; Short=Alpha-Alb; Flags: Precursor | 26,5 | 6 |
| gi\|39930812 | RecName: Full=Aflatoxin B1 aldehyde reductase member 2; Short=rAFAR2; AltName: Full=Succinic semialdehyde reductase; Short=SSA reductase | 15,5 | 4 |
| gi\|81883726 | RecName: Full=Alanyl-tRNA synthetase domain-containing protein 1 | 18,2 | 3 |
| gi\|84028322 | RecName: Full=Aldose 1-epimerase; AltName: Full=Galactose mutarotase | 15,2 | 2 |
| gi\|81872093 | macroglobulin 165 kDa subunit; Contains: RecName: Full=Alpha-1-macroglobulin 45 kDa subunit; Flags: Precursor | 17,9 | 11 |
| gi\|182627523 | RecName: Full=Alpha-centractin; Short=Centractin | 16,8 | 5 |
| gi\|81889858 | RecName: Full=Alpha-ketoglutarate-dependent dioxygenase alkB homolog 3; AltName: Full=Alkylated DNA repair protein alkB homolog 3 | 20 | 2 |
| gi\|20138815 | RecName: Full=Alpha-parvin; AltName: Full=Actopaxin | 13,2 | 2 |
| gi\|6094309 | RecName: Full=Alpha-soluble NSF attachment protein; Short=SNAP-alpha; AltName: Full=N-ethylmaleimide-sensitive factor attachment protein alpha | 32,9 | 6 |
| gi\|81884653 | RecName: Full=Aminoacylase-1A; AltName: Full=N-acyl-L-amino-acid amidohydrolase; AltName: Full=ACY-1A; AltName: Full=ACY IA | 19,6 | 5 |
| gi\|81901720 | RecName: Full=Ankyrin repeat domain-containing protein 1; AltName: Full=Cardiac ankyrin repeat protein; AltName: Full=Cardiac adriamycin-responsive protein | 37,6 | 8 |
| gi\|51315713 | Full=ATP synthase proteolipid P3; AltName: Full=ATPase protein 9; AltName: Full=ATPase subunit c; Flags: Precursor | 21,1 | 2 |
| gi\|461587 | RecName: Full=ATP synthase subunit e, mitochondrial | 71,8 | 12 |
| gi\|543880 | RecName: Full=ATP synthase subunit O, mitochondrial; AltName: Full=Oligomycin sensitivity conferral protein; Short=OSCP; Flags: Precursor | 62,4 | 17 |
| gi\|71152214 | AltName: Full=ATP-binding cassette transporter 7; Short=ABC transporter 7 protein; Flags: Precursor | 16,5 | 2 |

| | | | |
|---|---|---|---|
| gi\|56404328 | RecName: Full=ATP-binding cassette sub-family F member 1; AltName: Full=ATP-binding cassette 50 | 24,9 | 4 |
| gi\|81910247 | RecName: Full=ATP-dependent Clp protease ATP-binding subunit clpX-like, mitochondrial; Flags: Precursor | 25,8 | 3 |
| gi\|81909565 | Full=SUV3-like protein 1; AltName: Full=Suppressor of var1 3-like protein 1; Flags: Precursor | 16,4 | 3 |
| gi\|81917434 | glycoprotein; AltName: Full=Lutheran antigen; AltName: CD_antigen=CD239; Flags: Precursor | 20 | 6 |
| gi\|81885353 | RecName: Full=Basic leucine zipper and W2 domain-containing protein 1 | 21,5 | 2 |
| gi\|81882072 | RecName: Full=Basic leucine zipper and W2 domain-containing protein 2; AltName: Full=Brain development-related molecule 2 | 25,8 | 5 |
| gi\|81888008 | RecName: Full=Beta-lactamase-like protein 2 | 17,7 | 2 |
| gi\|67460101 | Full=Diadenosine 5',5'''-P1,P4-tetraphosphate asymmetrical hydrolase; Short=Diadenosine tetraphosphatase; Short=Ap4A hydrolase; Short=Ap4Aase; | 53,1 | 3 |
| gi\|81866115 | RecName: Full=Bone marrow stromal antigen 2; Short=BST-2; AltName: Full=Protein DAMP-1; AltName: CD_antigen=CD317; Flags: Precursor | 8,7 | 2 |
| gi\|730110 | membrane protein NAP-22; AltName: Full=22 kDa neuronal tissue-enriched acidic protein | 30 | 2 |
| gi\|730248 | RecName: Full=Brain protein 44; AltName: Full=Protein 0-44 | 28,4 | 3 |
| gi\|3023378 | RecName: Full=Branched-chain-amino-acid aminotransferase, mitochondrial; Short=BCAT(m); Flags: Precursor | 32,3 | 5 |
| gi\|81884168 | RecName: Full=Calcineurin-like phosphoesterase domain-containing protein 1 | 16,7 | 2 |
| gi\|81884615 | RecName: Full=Calcyclin-binding protein; Short=CacyBP | 29,7 | 2 |
| gi\|543922 | RecName: Full=Calnexin; Flags: Precursor | 36,6 | 16 |
| gi\|83301638 | dependent protease small subunit 1; AltName: Full=Calcium-dependent protease small subunit; Short=CDPS; AltName: Full=Calpain regulatory subunit; AltName: | 31,1 | 7 |
| gi\|205687264 | RecName: Full=Carbonic anhydrase 1; AltName: Full=Carbonic anhydrase I; Short=CA-I; AltName: Full=Carbonate dehydratase I | 34,1 | 9 |
| gi\|57013350 | AltName: Full=Carboxyesterase ES-10; AltName: Full=Fatty acid ethyl ester synthase; Short=FAEE synthase; AltName: Full=pI 6.1 esterase; AltName: Full=ES- | 18,6 | 6 |
| gi\|81894530 | RecName: Full=Carboxymethylenebutenolidase homolog; AltName: Full=Liver regeneration-related protein LRRG072 | 34,3 | 5 |
| gi\|729088 | RecName: Full=CD9 antigen; AltName: CD_antigen=CD9 | 16,8 | 2 |
| gi\|215275245 | RecName: Full=CDGSH iron sulfur domain-containing protein 1; AltName: Full=MitoNEET | 55,6 | 8 |
| gi\|85541752 | associated antigen MUC18; AltName: Full=Melanoma cell adhesion molecule; AltName: Full=Gicerin; AltName: CD_antigen=CD146; Flags: Precursor | 13,1 | 3 |
| gi\|3023522 | RecName: Full=Coatomer subunit beta'; AltName: Full=Beta'-coat protein; Short=Beta'-COP; AltName: Full=p102 | 11,9 | 3 |
| gi\|81884175 | RecName: Full=Coatomer subunit delta; AltName: Full=Delta-coat protein; Short=Delta-COP; Short=Archain | 21,7 | 3 |
| gi\|47115521 | 2; Short=SGN2; AltName: Full=JAB1-containing signalosome subunit 2; AltName: Full=Thyroid receptor-interacting protein 15; Short=TRIP-15; AltName: Full=Alien | 27,1 | 3 |
| gi\|81884373 | RecName: Full=COP9 signalosome complex subunit 3; Short=Signalosome subunit 3; Short=SGN3 | 17,5 | 4 |
| gi\|67460104 | Full=Cullin-associated and neddylation-dissociated protein 1; AltName: Full=p120 CAND1; AltName: Full=TBP-interacting protein TIP120A; AltName: Full=TBP- | 18,4 | 6 |
| gi\|67460125 | Full=Cullin-associated and neddylation-dissociated protein 2; AltName: Full=p120 CAND2; AltName: Full=TBP-interacting protein TIP120B; AltName: Full=TBP- | 21,1 | 9 |
| gi\|81884378 | Full=Ubiquinol-cytochrome-c reductase complex core protein 1; Short=Core protein I; AltName: Full=Complex III subunit 1; Flags: Precursor | 54,4 | 56 |

| | | | |
|---|---|---|---|
| gi\|62511137 | Full=Ubiquinol-cytochrome c reductase complex 11 kDa protein; AltName: Full=Cytochrome c1 non-heme 11 kDa protein; AltName: Full=Mitochondrial hinge | 76,4 | 17 |
| gi\|81865392 | cytochrome c reductase complex ubiquinone-binding protein QP-C; AltName: Full=Ubiquinol-cytochrome c reductase complex 9.5 kDa protein; AltName: | 53,7 | 3 |
| gi\|73919261 | Full=Dynein light intermediate chain 1, cytosolic; Short=Dynein light chain A; Short=DLC-A | 13,2 | 2 |
| gi\|81884348 | AltName: Full=Leucyl aminopeptidase; AltName: Full=Leucine aminopeptidase 3; Short=LAP; AltName: Full=Proline aminopeptidase; AltName: Full=Prolyl | 31,4 | 8 |
| gi\|81892734 | RecName: Full=Cytosolic non-specific dipeptidase; AltName: Full=CNDP dipeptidase 2 | 11,2 | 3 |
| gi\|97048478 | RecName: Full=D-2-hydroxyglutarate dehydrogenase, mitochondrial; Flags: Precursor | 19,1 | 3 |
| gi\|6015047 | RecName: Full=Delta(3,5)-Delta(2,4)-dienoyl-CoA isomerase, mitochondrial; Flags: Precursor | 44 | 16 |
| gi\|81885266 | RecName: Full=Dihydrolipoyl dehydrogenase, mitochondrial; AltName: Full=Dihydrolipoamide dehydrogenase; Flags: Precursor | 57,8 | 38 |
| gi\|62512126 | oxoglutarate dehydrogenase complex, mitochondrial; AltName: Full=Dihydrolipoamide succinyltransferase component of 2-oxoglutarate | 45,8 | 36 |
| gi\|68565629 | Short=DIPP-1; AltName: Full=Diadenosine 5',5'''-P1,P6-hexaphosphate hydrolase 1; AltName: Full=Nucleoside diphosphate-linked moiety X motif 3; Short=Nudix | 38,7 | 4 |
| gi\|81885840 | associated dnaJ protein 3; AltName: Full=ERj3p; AltName: Full=ERdj3; AltName: Full=ER-associated Hsp40 co-chaperone; AltName: Full=ER-associated DNAJ; | 12,6 | 3 |
| gi\|62512124 | subunit 2; AltName: Full=Dolichyl-diphosphooligosaccharide--protein glycosyltransferase 63 kDa subunit; AltName: Full=Ribophorin-2; AltName: | 9,4 | 2 |
| gi\|48428847 | subunit DAD1; Short=Oligosaccharyl transferase subunit DAD1; AltName: Full=Defender against cell death 1; Short=DAD-1 | 19,5 | 2 |
| gi\|2506256 | RecName: Full=Dynactin subunit 1; AltName: Full=150 kDa dynein-associated polypeptide; AltName: Full=DAP-150; Short=DP-150; AltName: Full=p150-glued | 31,2 | 10 |
| gi\|81884599 | RecName: Full=Dynactin subunit 2 | 32,8 | 7 |
| gi\|68566301 | RecName: Full=Dynamin-1-like protein; AltName: Full=Dynamin-like protein | 35,5 | 8 |
| gi\|56748619 | RecName: Full=Dynein light chain 2, cytoplasmic; AltName: Full=Dynein light chain LC8-type 2 | 42,7 | 2 |
| gi\|32469607 | RecName: Full=E3 ubiquitin-protein ligase NEDD4 | 15 | 2 |
| gi\|81910618 | RecName: Full=EH domain-containing protein 1 | 38,8 | 20 |
| gi\|81908709 | RecName: Full=EH domain-containing protein 2 | 32,2 | 11 |
| gi\|81884360 | RecName: Full=Electron transfer flavoprotein subunit beta; Short=Beta-ETF | 72,2 | 27 |
| gi\|50402096 | RecName: Full=Elongation factor 1-alpha 2; Short=EF-1-alpha-2; Short=Elongation factor 1 A-2; AltName: Full=eEF1A-2; AltName: Full=Statin S1 | 40,6 | 19 |
| gi\|190359305 | RecName: Full=Elongation factor Tu, mitochondrial; Flags: Precursor | 55,1 | 40 |
| gi\|81910882 | RecName: Full=Endophilin-B1; AltName: Full=SH3 domain-containing GRB2-like protein B1 | 28,2 | 8 |
| gi\|160185603 | RecName: Full=Endophilin-B2; AltName: Full=SH3 domain-containing GRB2-like protein B2 | 22,3 | 3 |
| gi\|62510525 | Full=Multiprotein-bridging factor 1; Short=MBF1; AltName: Full=Calmodulin-associated peptide 19; Short=CAP-19 | 34,5 | 3 |
| gi\|81883193 | RecName: Full=Enolase-phosphatase E1; AltName: Full=2,3-diketo-5-methylthio-1-phosphopentane phosphatase; AltName: Full=MASA homolog | 10,7 | 2 |
| gi\|229485399 | protein 2; AltName: Full=Stomatin-prohibitin-flotillin-HflC/K domain-containing protein 2; Short=SPFH domain-containing protein 2 | 18,6 | 4 |
| gi\|50400211 | AltName: Full=Oxidoreductin-1-L-alpha; AltName: Full=Endoplasmic oxidoreductin-1-like protein; AltName: Full=Global ischemia-induced protein 11; Flags: Precursor | 18,1 | 5 |

| | | | |
|---|---|---|---|
| gi\|83302472 | RecName: Full=ES1 protein homolog, mitochondrial; Flags: Precursor | 62,4 | 26 |
| gi\|81892005 | 17-beta-dehydrogenase 8; AltName: Full=17-beta-hydroxysteroid dehydrogenase 8; Short=17-beta-HSD 8; AltName: Full=3-oxoacyl-[acyl-carrier-protein] reductase | 27,4 | 3 |
| gi\|81889423 | RecName: Full=Eukaryotic initiation factor 4A-II; Short=eIF-4A-II; Short=eIF4A-II; AltName: Full=ATP-dependent RNA helicase eIF4A-2 | 27,8 | 4 |
| gi\|88909158 | AltName: Full=Eukaryotic translation initiation factor 3 subunit 9; AltName: Full=eIF-3-eta | 21,7 | 6 |
| gi\|224487979 | AltName: Full=Eukaryotic translation initiation factor 3 subunit 8; AltName: Full=eIF3 p110 | 16,3 | 6 |
| gi\|81884616 | AltName: Full=Eukaryotic translation initiation factor 3 subunit 7; AltName: Full=eIF-3-zeta | 17 | 2 |
| gi\|81884078 | AltName: Full=Eukaryotic translation initiation factor 3 subunit 6; AltName: Full=eIF-3 p48 | 18,7 | 5 |
| gi\|187471123 | AltName: Full=Eukaryotic translation initiation factor 3 subunit 1; AltName: Full=eIF-3-alpha; AltName: Full=eIF3 p35 | 37,5 | 5 |
| gi\|81910358 | RecName: Full=Eukaryotic translation initiation factor 4H; Short=eIF-4H; AltName: Full=Williams-Beuren syndrome chromosomal region 1 protein homolog | 26,2 | 4 |
| gi\|91207082 | Short=eIF-5A1; AltName: Full=Eukaryotic initiation factor 5A isoform 1; Short=eIF-5A; AltName: Full=eIF-4D | 21,4 | 3 |
| gi\|81883744 | RecName: Full=Evolutionarily conserved signaling intermediate in Toll pathway, mitochondrial; Flags: Precursor | 19,4 | 3 |
| gi\|68067388 | RecName: Full=Ezrin; AltName: Full=p81; AltName: Full=Cytovillin; AltName: Full=Villin-2 | 45,9 | 13 |
| gi\|81883689 | RecName: Full=F-actin-capping protein subunit beta; AltName: Full=CapZ beta | 33,1 | 2 |
| gi\|37078084 | protein 2; AltName: Full=KH type-splicing regulatory protein; Short=KSRP; AltName: Full=MAP2 RNA trans-acting protein 1; Short=MARTA1 | 29 | 5 |
| gi\|399362 | dehydrogenase family 3 member A2; AltName: Full=Aldehyde dehydrogenase 4; AltName: Full=Microsomal aldehyde dehydrogenase; Short=msALDH | 17,2 | 3 |
| gi\|81907626 | RecName: Full=Four and a half LIM domains protein 1; Short=FHL-1 | 32,9 | 6 |
| gi\|6226139 | Full=Skeletal muscle LIM-protein 3; Short=SLIM 3; AltName: Full=LIM domain protein DRAL | 14,3 | 4 |
| gi\|73919838 | RecName: Full=Fumarylacetoacetate hydrolase domain-containing protein 1 | 34,8 | 6 |
| gi\|229784139 | RecName: Full=Fumarylacetoacetate hydrolase domain-containing protein 2 | 22,4 | 3 |
| gi\|81892272 | Full=Phosphoglucose isomerase; Short=PGI; AltName: Full=Phosphohexose isomerase; Short=PHI; AltName: Full=Autocrine motility factor; Short=AMF; | 33,9 | 22 |
| gi\|81917962 | RecName: Full=Glutathione S-transferase Mu 5; AltName: Full=GST class-mu 5 | 30,7 | 3 |
| gi\|6225463 | RecName: Full=Glycogenin-1 | 16,8 | 4 |
| gi\|81914601 | N-myristoyltransferase 1; AltName: Full=Myristoyl-CoA:protein N-myristoyltransferase 1; Short=NMT 1; Short=Type I N-myristoyltransferase | 13,9 | 2 |
| gi\|81882968 | RecName: Full=Glyoxalase domain-containing protein 4 | 31,5 | 4 |
| gi\|8928123 | RecName: Full=GMP reductase 1; AltName: Full=Guanosine 5'-monophosphate oxidoreductase 1; Short=Guanosine monophosphate reductase 1 | 22,9 | 4 |
| gi\|81908630 | RecName: Full=GMP synthase [glutamine-hydrolyzing]; AltName: Full=Glutamine amidotransferase; AltName: Full=GMP synthetase | 9,1 | 2 |
| gi\|81882726 | RecName: Full=GRINL1A complex locus protein 1 | 28,8 | 3 |
| gi\|6016156 | RecName: Full=Growth factor receptor-bound protein 14; AltName: Full=GRB14 adapter protein | 16,9 | 2 |
| gi\|51338593 | RecName: Full=GTP-binding nuclear protein Ran; AltName: Full=GTPase Ran; AltName: Full=Ras-related nuclear protein; AltName: Full=Ras-like protein TC4 | 26,9 | 3 |

| | | | |
|---|---|---|---|
| gi\|81889008 | RecName: Full=GTP-binding protein SAR1b | 15,7 | 3 |
| gi\|54037164 | Full=Receptor of activated protein kinase C 1; Short=RACK1; AltName: Full=Receptor for activated C kinase | 20,2 | 4 |
| gi\|55977739 | RecName: Full=Heat shock 70 kDa protein 1A/1B; AltName: Full=Heat shock 70 kDa protein 1/2; AltName: Full=HSP70.1/2 | 45,4 | 30 |
| gi\|81886881 | RecName: Full=Heat shock 70 kDa protein 4; AltName: Full=Ischemia responsive 94 kDa protein | 40,4 | 23 |
| gi\|81890517 | RecName: Full=Heat shock protein 105 kDa; AltName: Full=Heat shock 110 kDa protein | 18,5 | 3 |
| gi\|6016269 | RecName: Full=Heat shock protein beta-2; Short=HspB2 | 53,9 | 5 |
| gi\|6016271 | RecName: Full=Heat shock protein beta-6; Short=HspB6; AltName: Full=Heat shock 20 kDa-like protein p20 | 41,4 | 12 |
| gi\|46576202 | RecName: Full=Heat shock protein beta-8; Short=HspB8; AltName: Full=Alpha-crystallin C chain; AltName: Full=Small stress protein-like protein HSP22 | 18,4 | 3 |
| gi\|71152130 | RecName: Full=Hematological and neurological expressed 1-like protein; Short=HN1-like protein | 21,6 | 2 |
| gi\|224493240 | RecName: Full=Heterogeneous nuclear ribonucleoproteins A2/B1; Short=hnRNP A2 / hnRNP B1 | 48,4 | 18 |
| gi\|76363184 | RecName: Full=HIG1 domain family member 1A; AltName: Full=Hypoxia-inducible gene 1 protein | 35,5 | 9 |
| gi\|60392384 | RecName: Full=Homeodomain-only protein; AltName: Full=Odd homeobox protein 1; AltName: Full=Global ischemia-induced protein 15B; AltName: Full=GIIg15b | 42,5 | 2 |
| gi\|81918176 | Short=HOT; AltName: Full=Alcohol dehydrogenase iron-containing protein 1; Flags: Precursor | 20,3 | 5 |
| gi\|81907928 | RecName: Full=Hydroxysteroid dehydrogenase-like protein 2 | 35,3 | 12 |
| gi\|81882496 | Short=Chaperone-ABC1-like; AltName: Full=aarF domain-containing protein kinase 3; Flags: Precursor | 32,1 | 12 |
| gi\|81911115 | RecName: Full=Chloride intracellular channel protein 1 | 14,1 | 2 |
| gi\|8469184 | sulfate proteoglycan NG2; AltName: Full=HSN tumor-specific antigen; Flags: Precursor | 7,8 | 2 |
| gi\|81916552 | RecName: Full=Integrin-linked protein kinase | 22,4 | 5 |
| gi\|68051964 | AltName: Full=Isocitric dehydrogenase; AltName: Full=NAD(+)-specific ICDH; Flags: Precursor | 33,5 | 10 |
| gi\|6166247 | AltName: Full=Isocitric dehydrogenase; AltName: Full=NAD(+)-specific ICDH; Flags: Precursor | 21,6 | 2 |
| gi\|81863750 | RecName: Full=Isochorismatase domain-containing protein 1; AltName: Full=Down-regulated in nephrectomized rat kidney protein 1; Short=DR-NR1 | 24,9 | 2 |
| gi\|81885083 | RecName: Full=Junction plakoglobin | 28,1 | 11 |
| gi\|81891690 | Short=CK-10; AltName: Full=Keratin-10; Short=K10; AltName: Full=Type I keratin Ka10 | 39,4 | 31 |
| gi\|81891674 | Short=CK-17; AltName: Full=Keratin-17; Short=K17; AltName: Full=Type I keratin Ka17 | 44,3 | 15 |
| gi\|81891673 | Short=CK-42; AltName: Full=Keratin-42; Short=K42; AltName: Full=Type I keratin Ka22 | 46 | 14 |
| gi\|81891699 | RecName: Full=Keratin, type II cytoskeletal 2 epidermal; AltName: Full=Cytokeratin-2e; Short=CK 2e; Short=K2e; Short=keratin-2; AltName: Full=Type II keratin Kb2 | 41,6 | 14 |
| gi\|81170669 | RecName: Full=Keratin, type II cytoskeletal 5; AltName: Full=Cytokeratin-5; Short=CK-5; AltName: Full=Keratin-5; Short=K5 | 46,4 | 33 |
| gi\|81871585 | associated protein 2; AltName: Full=Sam68-like mammalian protein 1; Short=rSLM-1; Short=SLM-1 | 25,2 | 2 |
| gi\|215274103 | RecName: Full=Kinesin light chain 1; Short=KLC 1 | 16,1 | 2 |

| | | | |
|---|---|---|---|
| gi\|81909953 | RecName: Full=Kinesin light chain 4; Short=KLC 4; AltName: Full=Kinesin-like protein 8 | 17,8 | 2 |
| gi\|84028216 | RecName: Full=Kinesin-like protein KIF1B | 10,9 | 2 |
| gi\|81861882 | Full=Kynurenine--oxoglutarate transaminase III; AltName: Full=Kynurenine aminotransferase III; Short=KATIII; AltName: Full=Cysteine-S-conjugate beta-lyase | 22,7 | 4 |
| gi\|81885359 | AltName: Full=Aldoketomutase; AltName: Full=Glyoxalase I; Short=Glx I; AltName: Full=Ketone-aldehyde mutase; AltName: Full=S-D-lactoylglutathione methylglyoxal | 51,1 | 6 |
| gi\|81873626 | RecName: Full=Lambda-crystallin homolog; AltName: Full=L-gulonate 3-dehydrogenase; Short=Gul3DH | 19,1 | 4 |
| gi\|81883487 | RecName: Full=LDLR chaperone MESD; AltName: Full=Mesoderm development candidate 2; AltName: Full=Mesoderm development protein; Flags: Precursor | 28,6 | 2 |
| gi\|62510718 | AltName: Full=Leucine zipper-EF-hand-containing transmembrane protein 1; Flags: Precursor | 40,1 | 17 |
| gi\|81910029 | RecName: Full=Leucine-rich repeat-containing protein 59; AltName: Full=Protein p34 | 29 | 2 |
| gi\|81883774 | synthase; AltName: Full=Lipoate synthase; Short=Lip-syn; Short=LS; Flags: Precursor | 14,2 | 2 |
| gi\|81916424 | like protein; Short=LONP; AltName: Full=Mitochondrial ATP-dependent protease Lon; AltName: Full=Serine protease 15; Flags: Precursor | 29,1 | 12 |
| gi\|81861572 | RecName: Full=Malate dehydrogenase, cytoplasmic; AltName: Full=Cytosolic malate dehydrogenase | 65,6 | 43 |
| gi\|67460103 | RecName: Full=Malonyl-CoA decarboxylase, mitochondrial; Short=MCD; Flags: Precursor | 27,9 | 7 |
| gi\|62510703 | RecName: Full=Mannose-6-phosphate isomerase; AltName: Full=Phosphomannose isomerase; Short=PMI; AltName: Full=Phosphohexomutase | 13,2 | 2 |
| gi\|76363234 | AltName: Full=Macrophage myristoylated alanine-rich C kinase substrate; Short=Mac-MARCKS; Short=MacMARCKS; AltName: Full=Brain protein F52 | 19,6 | 2 |
| gi\|62900631 | RecName: Full=Membrane-associated progesterone receptor component 2 | 21,7 | 4 |
| gi\|81883845 | Short=MCCase subunit beta; AltName: Full=3-methylcrotonyl-CoA:carbon dioxide ligase subunit beta; AltName: Full=3-methylcrotonyl-CoA carboxylase non-biotin- | 41 | 22 |
| gi\|81882966 | Short=MCCase subunit alpha; AltName: Full=3-methylcrotonyl-CoA carboxylase 1; AltName: Full=3-methylcrotonyl-CoA:carbon dioxide ligase subunit alpha; AltName: | 34,3 | 16 |
| gi\|400269 | mitochondrial; Short=MMSDH; Short=Malonate-semialdehyde dehydrogenase [acylating]; AltName: Full=Aldehyde dehydrogenase family 6 member A1; Flags: | 60 | 80 |
| gi\|81882932 | isomerase; Short=M1Pi; AltName: Full=S-methyl-5-thioribose-1-phosphate isomerase; AltName: Full=Translation initiation factor eIF-2B subunit | 15,7 | 2 |
| gi\|13432197 | RecName: Full=Microtubule-associated protein tau; AltName: Full=Neurofibrillary tangle protein; AltName: Full=Paired helical filament-tau; Short=PHF-tau | 17,3 | 4 |
| gi\|47605758 | RecName: Full=Mitofusin-1; AltName: Full=Transmembrane GTPase MFN1; AltName: Full=Mitochondrial transmembrane GTPase FZO1B | 21,5 | 3 |
| gi\|81170680 | beta promoter stimulator protein 1; Short=IPS-1; AltName: Full=Virus-induced-signaling adapter | 11,1 | 2 |
| gi\|2497984 | Full=Carnitine/acylcarnitine translocase; Short=CAC; AltName: Full=Solute carrier family 25 member 20 | 34,2 | 6 |
| gi\|94711371 | Short=rFis1; AltName: Full=Tetratricopeptide repeat protein 11; AltName: Full=TPR repeat protein 11 | 42,1 | 4 |
| gi\|59800393 | RecName: Full=Mitochondrial import inner membrane translocase subunit Tim13 | 74,7 | 17 |
| gi\|90110082 | RecName: Full=Mitochondrial import inner membrane translocase subunit Tim9 | 42,7 | 2 |
| gi\|81864913 | RecName: Full=Mitochondrial import receptor subunit TOM22 homolog; AltName: Full=Translocase of outer membrane 22 kDa subunit homolog; Short=rTOM22 | 48,6 | 3 |
| gi\|81864912 | Full=Translocase of outer membrane 40 kDa subunit homolog; AltName: Full=Mitochondrial outer membrane protein of 38 kDa; Short=OM38 | 12,7 | 2 |
| gi\|81911805 | Full=Translocase of outer membrane 70 kDa subunit; AltName: Full=Mitochondrial precursor proteins import receptor | 28,5 | 6 |

| | | | |
|---|---|---|---|
| gi\|67460631 | RecName: Full=Motile sperm domain-containing protein 1 | 8 | 2 |
| gi\|81870614 | RecName: Full=Murinoglobulin-1; AltName: Full=Alpha-1 inhibitor 3 variant I; AltName: Full=Alpha-X protein; Flags: Precursor | 31,3 | 27 |
| gi\|226711873 | RecName: Full=Muscle-related coiled-coil protein; AltName: Full=Muscle-restricted coiled-coil protein | 21,6 | 3 |
| gi\|78099013 | RecName: Full=Myoglobin | 98,7 | 182 |
| gi\|266495 | RecName: Full=Myristoylated alanine-rich C-kinase substrate; Short=MARCKS; AltName: Full=Protein kinase C substrate 80 kDa protein | 55,7 | 10 |
| gi\|6831527 | Short=Dimethylarginine dimethylaminohydrolase 1; Short=DDAH-1; AltName: Full=DDAHI; AltName: Full=Dimethylargininase-1 | 21,8 | 5 |
| gi\|81911114 | Short=Dimethylarginine dimethylaminohydrolase 2; Short=DDAH-2; AltName: Full=DDAHII; AltName: Full=Dimethylargininase-2 | 20,7 | 3 |
| gi\|42559161 | 2; AltName: Full=Tyrosine kinase activator protein 1; Short=TKA-1; AltName: Full=SRY-interacting protein 1; Short=SIP-1; AltName: Full=Solute carrier family 9 | 33,2 | 2 |
| gi\|81884377 | RecName: Full=NAD-dependent deacetylase sirtuin-5; Flags: Precursor | 16,5 | 4 |
| gi\|81882328 | 10, mitochondrial; AltName: Full=NADH-ubiquinone oxidoreductase 42 kDa subunit; AltName: Full=Complex I-42kD; Short=CI-42kD; Flags: Precursor | 57,2 | 27 |
| gi\|52000746 | 11; AltName: Full=NADH-ubiquinone oxidoreductase subunit B14.7; AltName: Full=Complex I-B14.7; Short=CI-B14.7 | 19,9 | 5 |
| gi\|83305118 | RecName: Full=NADH dehydrogenase [ubiquinone] flavoprotein 2, mitochondrial; AltName: Full=NADH-ubiquinone oxidoreductase 24 kDa subunit; Flags: Precursor | 54,8 | 29 |
| gi\|81890431 | mitochondrial; AltName: Full=NADH-ubiquinone oxidoreductase 49 kDa subunit; AltName: Full=Complex I-49kD; Short=CI-49kD; Flags: Precursor | 50,3 | 27 |
| gi\|81889861 | mitochondrial; AltName: Full=NADH-ubiquinone oxidoreductase 18 kDa subunit; AltName: Full=Complex I-18 kDa; Short=CI-18 kDa; Flags: Precursor | 44,6 | 24 |
| gi\|81882716 | RecName: Full=NADH-cytochrome b5 reductase 1; Short=b5R.1 | 23,3 | 2 |
| gi\|81884209 | RecName: Full=NADH-ubiquinone oxidoreductase 75 kDa subunit, mitochondrial; Flags: Precursor | 61,1 | 75 |
| gi\|266504 | RecName: Full=NADP-dependent malic enzyme; Short=NADP-ME; AltName: Full=Malic enzyme 1 | 24,7 | 5 |
| gi\|212288176 | RecName: Full=Neutral cholesterol ester hydrolase 1; Short=NCEH; AltName: Full=Arylacetamide deacetylase-like 1 | 17,2 | 5 |
| gi\|81918008 | RecName: Full=Nexilin | 43,3 | 6 |
| gi\|68565643 | Short=Nampt; AltName: Full=Pre-B-cell colony-enhancing factor 1 homolog; Short=PBEF; AltName: Full=Visfatin | 30,6 | 6 |
| gi\|81888874 | RecName: Full=NLR family member X1; Flags: Precursor | 13,8 | 2 |
| gi\|462266 | RecName: Full=Non-histone chromosomal protein HMG-17; AltName: Full=High-mobility group nucleosome-binding domain-containing protein 2 | 33,3 | 2 |
| gi\|67460593 | RecName: Full=Non-POU domain-containing octamer-binding protein; Short=NonO protein | 24,6 | 2 |
| gi\|462690 | Short=NDK A; AltName: Full=Tumor metastatic process-associated protein; AltName: Full=Metastasis inhibition factor NM23 | 45,4 | 13 |
| gi\|81883530 | RecName: Full=Nucleosome assembly protein 1-like 4 | 31,4 | 7 |
| gi\|81883766 | RecName: Full=OCIA domain-containing protein 1 | 42,9 | 6 |
| gi\|81910234 | RecName: Full=Oligoribonuclease, mitochondrial; AltName: Full=Small fragment nuclease; AltName: Full=RNA exonuclease 2 homolog; Flags: Precursor | 27 | 2 |
| gi\|81901134 | RecName: Full=Oxysterol-binding protein-related protein 1; Short=OSBP-related protein 1; Short=ORP-1 | 11,8 | 2 |
| gi\|81912692 | RecName: Full=Paraplegin | 17,6 | 2 |

| | | | |
|---|---|---|---|
| gi\|5902791 | Full=1-Cys peroxiredoxin; Short=1-Cys PRX; AltName: Full=Acidic calcium-independent phospholipase A2; Short=aiPLA2; AltName: Full=Non-selenium | 58 | 21 |
| gi\|81883743 | AltName: Full=Dodecenoyl-CoA isomerase; AltName: Full=Delta(3),delta(2)-enoyl-CoA isomerase; Short=D3,D2-enoyl-CoA isomerase; Flags: Precursor | 40,9 | 19 |
| gi\|67460102 | diglyceride pyrophosphorylase 2; AltName: Full=CDP-diglyceride synthetase 2; AltName: Full=CDP-diacylglycerol synthase 2; Short=CDS 2; AltName: Full=CDP- | 16 | 4 |
| gi\|22654268 | RecName: Full=Phospholemman; AltName: Full=FXYD domain-containing ion transport regulator 1; Flags: Precursor | 31,5 | 3 |
| gi\|226693553 | RecName: Full=Plastin-3; AltName: Full=T-plastin | 18,9 | 3 |
| gi\|51702760 | AltName: Full=PAF acetylhydrolase 45 kDa subunit; Short=PAF-AH 45 kDa subunit; AltName: Full=PAF-AH alpha; AltName: Full=PAFAH alpha; AltName: | 18,8 | 4 |
| gi\|73919763 | RecName: Full=Probable prolyl-tRNA synthetase, mitochondrial; AltName: Full=Proline--tRNA ligase; Short=ProRS; Flags: Precursor | 11,4 | 3 |
| gi\|73919297 | RecName: Full=Probable saccharopine dehydrogenase | 10 | 2 |
| gi\|76363296 | RecName: Full=Prohibitin-2; AltName: Full=B-cell receptor-associated protein BAP37; Short=BAP-37 | 53,9 | 15 |
| gi\|206558239 | oxoprostaglandin 13-reductase; AltName: Full=Zinc-binding alcohol dehydrogenase domain-containing protein 1 | 24,2 | 7 |
| gi\|78099786 | Short=ERp72; AltName: Full=Calcium-binding protein 2; Short=CaBP2; Flags: Precursor | 18,7 | 5 |
| gi\|62296810 | isomerase P5; AltName: Full=Calcium-binding protein 1; Short=CaBP1; AltName: Full=Thioredoxin domain-containing protein 7; Flags: Precursor | 25,7 | 6 |
| gi\|56404680 | AltName: Full=Contraception-associated protein 1; Short=Protein CAP1; AltName: Full=Fertility protein SP22 | 61,4 | 12 |
| gi\|187471128 | RecName: Full=Protein FAM136A | 31,2 | 2 |
| gi\|81882907 | RecName: Full=Protein FAM98A | 26 | 2 |
| gi\|81870080 | deprivation response factor-related gene product that binds to C-kinase; AltName: Full=Dithiolethione-inducible gene 2 protein; AltName: Full=D3T-inducible gene 2 | 21,7 | 2 |
| gi\|81883247 | domain-containing protein; AltName: Full=Leucine zipper and CTNNBIP1 domain-containing protein | 33,2 | 2 |
| gi\|81918134 | RecName: Full=Protein MEMO1; AltName: Full=Mediator of ErbB2-driven cell motility 1; Short=Protein memo | 27,6 | 3 |
| gi\|81867103 | RecName: Full=Protein NDRG2; AltName: Full=NDRG1-related protein; AltName: Full=Antidepressant-related protein ADRG123 | 39,9 | 7 |
| gi\|81909701 | RecName: Full=Protein phosphatase 1 regulatory subunit 7; AltName: Full=Protein phosphatase 1 regulatory subunit 22 | 31,7 | 6 |
| gi\|81889646 | RecName: Full=Protein preY, mitochondrial; Flags: Precursor | 32,1 | 2 |
| gi\|81910340 | RecName: Full=Protein SEC13 homolog; AltName: Full=SEC13-like protein 1 | 9,6 | 2 |
| gi\|81883171 | RecName: Full=Protein TBRG4; AltName: Full=Transforming growth factor beta regulator 4 | 12,9 | 2 |
| gi\|152112289 | RecName: Full=Protein transport protein Sec31A; AltName: Full=SEC31-related protein A; AltName: Full=SEC31-like 1; AltName: Full=Vesicle-associated protein 1 | 11,7 | 3 |
| gi\|73621422 | RecName: Full=Protein-tyrosine phosphatase mitochondrial 1; Flags: Precursor | 22,3 | 2 |
| gi\|205829287 | RecName: Full=Purine nucleoside phosphorylase; Short=PNP; AltName: Full=Inosine phosphorylase | 22,8 | 6 |
| gi\|81884356 | RecName: Full=Pyridine nucleotide-disulfide oxidoreductase domain-containing protein 2 | 24,1 | 5 |
| gi\|71152023 | RecName: Full=Quinone oxidoreductase; AltName: Full=NADPH:quinone reductase; AltName: Full=Zeta-crystallin | 15,5 | 2 |
| gi\|190360156 | RecName: Full=Quinone oxidoreductase-like protein 2 | 8,6 | 2 |

| | | | |
|---|---|---|---|
| gi\|46577632 | RecName: Full=Ras-related protein Rab-14 | 20,9 | 5 |
| gi\|81884468 | RecName: Full=Ras-related protein Rab-21 | 12,6 | 2 |
| gi\|62900797 | RecName: Full=Ras-related protein Rab-35 | 37,8 | 9 |
| gi\|2500066 | RecName: Full=Ras-related protein Rab-8B | 39,6 | 6 |
| gi\|48474727 | RecName: Full=Retinoid-inducible serine carboxypeptidase; AltName: Full=Serine carboxypeptidase 1; Flags: Precursor | 14,6 | 2 |
| gi\|81883710 | RecName: Full=Rho GDP-dissociation inhibitor 1; Short=Rho GDI 1; AltName: Full=Rho-GDI alpha | 49,5 | 4 |
| gi\|62900751 | RecName: Full=Ribosome-recycling factor, mitochondrial; Short=RRF; AltName: Full=Ribosome-releasing factor, mitochondrial; Flags: Precursor | 19,5 | 2 |
| gi\|81884542 | Full=NRH dehydrogenase [quinone] 2; AltName: Full=NRH:quinone oxidoreductase 2; Short=Quinone reductase 2; Short=QR2 | 24,7 | 2 |
| gi\|81879451 | protein 2; AltName: Full=56 kDa selenium-binding protein; AltName: Full=SBP56; Short=SP56 | 36,2 | 13 |
| gi\|81902430 | RecName: Full=Septin-2; AltName: Full=Vascular endothelial cell specific protein 11 | 44,3 | 4 |
| gi\|224493301 | RecName: Full=Septin-8 | 22,2 | 3 |
| gi\|2507388 | Full=Contrapsin-like protease inhibitor 6; AltName: Full=CPI-26; AltName: Full=Serine protease inhibitor 3; Short=SPI-3; AltName: Full=SPI-2.2; Flags: | 30,1 | 10 |
| gi\|81912127 | RecName: Full=Serine/threonine-protein kinase TNNI3K; AltName: Full=TNNI3-interacting kinase; AltName: Full=Cardiac ankyrin repeat kinase | 8,1 | 2 |
| gi\|543717 | subunit B alpha isoform; Short=PP2A, subunit B, B-alpha isoform; AltName: Full=PP2A, subunit B, B55-alpha isoform; AltName: Full=PP2A, subunit B, PR55- | 19,5 | 3 |
| gi\|81883769 | RecName: Full=Serine-threonine kinase receptor-associated protein; AltName: Full=UNR-interacting protein | 19,7 | 3 |
| gi\|81884184 | RecName: Full=Serum deprivation-response protein; AltName: Full=Phosphatidylserine-binding protein | 41 | 14 |
| gi\|417812 | SSB; Short=MtSSB; AltName: Full=Single strand DNA-binding protein P16; Flags: Precursor | 39,1 | 4 |
| gi\|8134664 | alpha; AltName: Full=Alpha-SGT; AltName: Full=Small glutamine-rich protein with tetratricopeptide repeats 1 | 16,2 | 2 |
| gi\|156633625 | RecName: Full=Solute carrier family 12 member 7; AltName: Full=Electroneutral potassium-chloride cotransporter 4; AltName: Full=K-Cl cotransporter 4 | 13,1 | 3 |
| gi\|81884480 | RecName: Full=Sorting and assembly machinery component 50 homolog | 24,1 | 6 |
| gi\|81883455 | RecName: Full=Sorting nexin-3 | 33,3 | 2 |
| gi\|61252099 | UAP56; AltName: Full=56 kDa U2AF65-associated protein; AltName: Full=ATP-dependent RNA helicase p47 | 25,9 | 4 |
| gi\|81908254 | kinase; AltName: Full=STE20-related serine/threonine-protein kinase; Short=STE20-related kinase | 22,8 | 6 |
| gi\|116242506 | regulated protein; AltName: Full=GRP 75; AltName: Full=Heat shock 70 kDa protein 9; AltName: Full=Peptide-binding protein 74; Short=PBP74; AltName: | 55,5 | 61 |
| gi\|54036435 | RecName: Full=Stress-induced-phosphoprotein 1; Short=STI1; AltName: Full=Hsc70/Hsp90-organizing protein; Short=Hop | 29,8 | 13 |
| gi\|97537204 | RecName: Full=Striated muscle-specific serine/threonine-protein kinase; AltName: Full=Aortic preferentially expressed protein 1; AltName: Full=APEG-1 | 10,5 | 5 |
| gi\|52782765 | mitochondrial; AltName: Full=Flavoprotein subunit of complex II; Short=Fp; Flags: Precursor | 61 | 58 |
| gi\|81883340 | Short=SDH assembly factor 2; AltName: Full=Succinate dehydrogenase subunit 5, mitochondrial; Flags: Precursor | 22 | 2 |
| gi\|182676407 | Full=NAD(+)-dependent succinic semialdehyde dehydrogenase; AltName: Full=Aldehyde dehydrogenase family 5 member A1; Flags: Precursor | 22,8 | 8 |

| | | | |
|---|---|---|---|
| gi\|223634703 | AltName: Full=Succinyl-CoA synthetase subunit alpha; Short=SCS-alpha; Flags: Precursor | 29,8 | 15 |
| gi\|205829936 | AltName: Full=3-oxoacid-CoA transferase 1; AltName: Full=Somatic-type succinyl CoA:3-oxoacid CoA-transferase; Short=Scot-S; Flags: Precursor | 50,4 | 47 |
| gi\|41017815 | RecName: Full=Synaptosomal-associated protein 23; Short=SNAP-23; AltName: Full=Vesicle-membrane fusion protein SNAP-23 | 29,5 | 2 |
| gi\|81910374 | RecName: Full=T-complex protein 1 subunit beta; Short=TCP-1-beta; AltName: Full=CCT-beta | 19,8 | 5 |
| gi\|52000745 | RecName: Full=T-complex protein 1 subunit delta; Short=TCP-1-delta; AltName: Full=CCT-delta | 24,3 | 5 |
| gi\|81910761 | RecName: Full=T-complex protein 1 subunit epsilon; Short=TCP-1-epsilon; AltName: Full=CCT-epsilon | 28,7 | 3 |
| gi\|81911258 | RecName: Full=T-complex protein 1 subunit gamma; Short=TCP-1-gamma; AltName: Full=CCT-gamma | 29,7 | 8 |
| gi\|189036952 | RecName: Full=Tetratricopeptide repeat protein 35; Short=TPR repeat protein 35 | 28 | 2 |
| gi\|81888054 | RecName: Full=Thioesterase superfamily member 4 | 11,7 | 2 |
| gi\|81916316 | RecName: Full=Thioredoxin-like protein 1; AltName: Full=Thioredoxin-related protein | 26,3 | 3 |
| gi\|81883793 | RecName: Full=Thioredoxin-related transmembrane protein 2; AltName: Full=Thioredoxin domain-containing protein 14; Flags: Precursor | 20,3 | 3 |
| gi\|83305808 | RecName: Full=Thiosulfate sulfurtransferase; AltName: Full=Rhodanese | 31 | 4 |
| gi\|81884372 | Full=Threonine--tRNA ligase; Short=ThrRS; AltName: Full=Threonyl-tRNA synthetase-like 1; Flags: Precursor | 14,5 | 4 |
| gi\|78103212 | RecName: Full=Thymosin beta-4; Short=T beta 4; Contains: RecName: Full=Hematopoietic system regulatory peptide; AltName: Full=Seraspenide | 40,9 | 6 |
| gi\|73918915 | RecName: Full=TIM21-like protein, mitochondrial; Flags: Precursor | 32,2 | 6 |
| gi\|160184946 | RecName: Full=Toll-interacting protein | 28,1 | 3 |
| gi\|71153350 | RecName: Full=Trafficking protein particle complex subunit 3 | 36,7 | 2 |
| gi\|92090643 | RecName: Full=Transaldolase | 19,6 | 5 |
| gi\|51317294 | Full=RNA polymerase II transcription factor SIII subunit B; AltName: Full=SIII p18; AltName: Full=Elongin B; Short=EloB; AltName: Full=Elongin 18 kDa subunit | 31,4 | 4 |
| gi\|47605935 | RecName: Full=Transforming protein RhoA; Flags: Precursor | 40,4 | 8 |
| gi\|263511729 | Full=Translational activator of mitochondrially-encoded cytochrome c oxidase I; AltName: Full=Coiled-coil domain-containing protein 44 | 21,7 | 3 |
| gi\|62906894 | gamma; AltName: Full=Signal sequence receptor subunit gamma; Short=SSR-gamma | 11,4 | 2 |
| gi\|62906896 | Full=21 kDa transmembrane-trafficking protein; AltName: Full=Transmembrane protein Tmp21; Flags: Precursor | 22,8 | 3 |
| gi\|81882927 | RecName: Full=Transmembrane protein 126A | 22,5 | 3 |
| gi\|160358756 | Short=TRICA; AltName: Full=Transmembrane protein 38A; AltName: Full=27 kDa sarcoplasmic reticulum protein; AltName: Full=SPR-27 | 8,1 | 3 |
| gi\|81175036 | RecName: Full=Tripartite motif-containing protein 54; AltName: Full=RING finger protein 30 | 21,4 | 4 |
| gi\|92090646 | RecName: Full=Tropomyosin alpha-1 chain; AltName: Full=Tropomyosin-1; AltName: Full=Alpha-tropomyosin | 81,7 | 44 |
| gi\|55977470 | RecName: Full=Tubulin alpha-1A chain; AltName: Full=Tubulin alpha-1 chain; AltName: Full=Alpha-tubulin 1 | 61,9 | 41 |
| gi\|81889864 | RecName: Full=Tubulin alpha-4A chain; AltName: Full=Tubulin alpha-4 chain; AltName: Full=Alpha-tubulin 4 | 67,4 | 40 |

| | | | |
|---|---|---|---|
| gi\|56754676 | RecName: Full=Tubulin beta-5 chain | 48,4 | 26 |
| gi\|81883217 | RecName: Full=Tubulin polymerization-promoting protein family member 3 | 47,2 | 5 |
| gi\|81885679 | RecName: Full=Tubulin-specific chaperone A; AltName: Full=Tubulin-folding cofactor A; Short=CFA; AltName: Full=TCP1-chaperonin cofactor A | 43,5 | 5 |
| gi\|62511141 | RecName: Full=Tumor protein D54; AltName: Full=Tumor protein D52-like 2 | 33,2 | 3 |
| gi\|84028250 | Full=Protein-tyrosine phosphatase SYP; AltName: Full=PTP-1D; AltName: Full=SH-PTP2; Short=SHP-2; Short=Shp2 | 24,1 | 5 |
| gi\|90111992 | RecName: Full=Ubiquinone biosynthesis protein COQ9, mitochondrial; Flags: Precursor | 46,2 | 19 |
| gi\|68566104 | RecName: Full=Ubiquitin carboxyl-terminal hydrolase isozyme L3; Short=UCH-L3; AltName: Full=Ubiquitin thioesterase L3 | 24,4 | 2 |
| gi\|205829267 | Full=OTU domain-containing ubiquitin aldehyde-binding protein 1; AltName: Full=Ubiquitin-specific-processing protease OTUB1; AltName: | 32,1 | 5 |
| gi\|77417616 | protein ligase N; AltName: Full=Ubiquitin carrier protein N; AltName: Full=Bendless-like ubiquitin-conjugating enzyme | 41,5 | 7 |
| gi\|81889667 | RecName: Full=Ubiquitin-like modifier-activating enzyme 1; AltName: Full=Ubiquitin-activating enzyme E1 | 30,3 | 19 |
| gi\|76363460 | activating enzyme 5; AltName: Full=Ubiquitin-activating enzyme E1 domain-containing protein 1; AltName: Full=UFM1-activating enzyme | 15,4 | 2 |
| gi\|81882961 | RecName: Full=UBX domain-containing protein 4; AltName: Full=UBX domain-containing protein 2; AltName: Full=Erasin | 19,8 | 2 |
| gi\|81883788 | RecName: Full=Uncharacterized protein C18orf19 homolog | 29,3 | 4 |
| gi\|81884655 | RecName: Full=UPF0027 protein C22orf28 homolog; AltName: Full=p55 | 11,5 | 2 |
| gi\|81884656 | RecName: Full=UPF0364 protein C6orf211 homolog | 17,8 | 6 |
| gi\|81918167 | RecName: Full=UPF0389 protein FAM162A; AltName: Full=E2-induced gene 5 protein homolog | 49 | 4 |
| gi\|81884088 | RecName: Full=UPF0598 protein C8orf82 homolog | 20,2 | 2 |
| gi\|81868653 | RecName: Full=Up-regulated during skeletal muscle growth protein 5; AltName: Full=Diabetes-associated protein in insulin-sensitive tissues | 43,1 | 16 |
| gi\|78099277 | RecName: Full=Vacuolar protein sorting-associated protein 26A; AltName: Full=Vesicle protein sorting 26A | 38,2 | 4 |
| gi\|224493432 | RecName: Full=Vacuolar protein sorting-associated protein 29; AltName: Full=Vesicle protein sorting 29 | 26,9 | 3 |
| gi\|73919325 | RecName: Full=Vacuolar-sorting protein SNF8; AltName: Full=ESCRT-II complex subunit VPS22; AltName: Full=ELL-associated protein of 30 kDa | 28,3 | 2 |
| gi\|73920806 | RecName: Full=Valyl-tRNA synthetase; AltName: Full=Valine--tRNA ligase; Short=ValRS | 19,7 | 14 |
| gi\|81892462 | RecName: Full=V-type proton ATPase subunit E 1; Short=V-ATPase subunit E 1; AltName: Full=Vacuolar proton pump subunit E 1 | 26,6 | 3 |
| gi\|1718093 | AltName: Full=Vacuolar proton pump subunit F; AltName: Full=V-ATPase 14 kDa subunit | 33,6 | 3 |
| gi\|81910041 | RecName: Full=WD repeat-containing protein 1 | 25,6 | 10 |
| gi\|81889014 | Full=Proline dipeptidase; Short=Prolidase; AltName: Full=Imidodipeptidase; AltName: Full=Peptidase D | 15,2 | 2 |
| gi\|4079713 | reggie1-4 [Rattus norvegicus] | 41,2 | 6 |
| gi\|61740635 | related RAS viral (r-ras) oncogene homolog 2 [Rattus norvegicus] | 46,1 | 7 |
| gi\|149063305 | restin (Reed-Steinberg cell-expressed intermediate filament-associated protein), isoform CRA_b [Rattus norvegicus] | 28,3 | 3 |

| | | | |
|---|---|---|---|
| gi\|157819753 | reticulocalbin 1, EF-hand calcium binding domain [Rattus norvegicus] | 25,5 | 3 |
| gi\|183985843 | Reticulon 4 interacting protein 1 [Rattus norvegicus] | 23,5 | 4 |
| gi\|197246361 | RGD1310159 protein [Rattus norvegicus] | 18,5 | 10 |
| gi\|211065497 | Rho GTPase activating protein 1 [Rattus norvegicus] | 25,5 | 2 |
| gi\|57527565 | Rho, GDP dissociation inhibitor (GDI) beta [Rattus norvegicus] | 40 | 2 |
| gi\|6981478 | Rho-associated coiled-coil containing protein kinase 2 [Rattus norvegicus] | 29,9 | 7 |
| gi\|887580 | ri1 [Rattus norvegicus] | 13,6 | 2 |
| gi\|149061561 | ribonuclease/angiogenin inhibitor 1, isoform CRA_b [Rattus norvegicus] | 26,6 | 5 |
| gi\|736292 | ribophorin I [Rattus norvegicus] | 26 | 9 |
| gi\|77404207 | ribosomal protein L13A [Rattus norvegicus] | 18,2 | 2 |
| gi\|34849736 | Ribosomal protein L36 [Rattus norvegicus] | 20 | 2 |
| gi\|78214309 | ribosomal protein L8 [Rattus norvegicus] | 14,8 | 2 |
| gi\|57164151 | ribosomal protein S3 [Rattus norvegicus] | 16,9 | 3 |
| gi\|8394221 | ribosomal protein S3a [Rattus norvegicus] | 27,3 | 6 |
| gi\|56090273 | ribosomal protein S4, X-linked [Rattus norvegicus] | 24 | 4 |
| gi\|225901 | ribosomal protein S6 | 29,7 | 3 |
| gi\|71795613 | ribosomal protein, large P2 [Rattus norvegicus] | 37,4 | 3 |
| gi\|71043834 | RNA binding motif protein, X-linked [Rattus norvegicus] | 26,9 | 4 |
| gi\|9588100 | RNA binding protein p40 AUF1 [Rattus norvegicus] | 35,5 | 3 |
| gi\|51948426 | RNA terminal phosphate cyclase domain 1 [Rattus norvegicus] | 12,3 | 2 |
| gi\|1585294 | RNH-1/14-3-3 protein | 47,2 | 12 |
| gi\|38511552 | Rpl6 protein [Rattus norvegicus] | 20,5 | 4 |
| gi\|717146 | rTOM20 [Rattus norvegicus] | 24,8 | 2 |
| gi\|60551888 | S100 calcium binding protein A10 [Rattus norvegicus] | 24,2 | 3 |
| gi\|6981326 | S100 calcium-binding protein A4 [Rattus norvegicus] | 29,7 | 2 |
| gi\|165971609 | S100a16 protein [Rattus norvegicus] | 26,8 | 2 |
| gi\|157823585 | sarcoglycan, alpha [Rattus norvegicus] | 28,9 | 6 |
| gi\|157823857 | sarcospan [Rattus norvegicus] | 9,7 | 2 |
| gi\|157786908 | SCAN domain containing 3 [Rattus norvegicus] | 16,4 | 3 |

| | | | |
|---|---|---|---|
| gi\|38197654 | Scavenger receptor class B, member 2 [Rattus norvegicus] | 16,5 | 3 |
| gi\|71043604 | SEC22 vesicle trafficking protein homolog B [Rattus norvegicus] | 38,6 | 2 |
| gi\|197246881 | Sec23 homolog A (S. cerevisiae) [Rattus norvegicus] | 11,4 | 2 |
| gi\|149022245 | secernin 3, isoform CRA_a [Rattus norvegicus] | 22 | 2 |
| gi\|206896 | sepiapterin reductase (EC 1.1.1.153) [Rattus norvegicus] | 13,9 | 2 |
| gi\|183985886 | Sept9 protein [Rattus norvegicus] | 23,1 | 4 |
| gi\|149027897 | septin 7, isoform CRA_b [Rattus norvegicus] | 39,4 | 4 |
| gi\|58865630 | serine (or cysteine) peptidase inhibitor, clade C (antithrombin), member 1 [Rattus norvegicus] | 43,4 | 8 |
| gi\|8393057 | serine (or cysteine) peptidase inhibitor, clade H, member 1 precursor [Rattus norvegicus] | 24,5 | 3 |
| gi\|56961650 | serine (or cysteine) proteinase inhibitor, clade A (alpha-1 antiproteinase, antitrypsin), member 4 [Rattus norvegicus] | 13,2 | 2 |
| gi\|40018548 | serine (or cysteine) proteinase inhibitor, clade B (ovalbumin), member 6 [Rattus norvegicus] | 12,9 | 3 |
| gi\|56605722 | serine hydroxymethyltransferase 2 (mitochondrial) [Rattus norvegicus] | 21,2 | 3 |
| gi\|51036655 | serine protease inhibitor alpha 1 precursor [Rattus norvegicus] | 38,9 | 30 |
| gi\|4584820 | serine/threonine specific protein phosphatase [Rattus norvegicus] | 15,9 | 3 |
| gi\|157819737 | seryl-tRNA synthetase 2 [Rattus norvegicus] | 24,5 | 5 |
| gi\|157819445 | SET and MYND domain containing 1 [Rattus norvegicus] | 29,8 | 9 |
| gi\|741750 | set gene | 22,8 | 3 |
| gi\|166796515 | Sh3bgr protein [Rattus norvegicus] | 51,3 | 10 |
| gi\|149042430 | SH3-domain kinase binding protein 1, isoform CRA_c [Rattus norvegicus] | 20,5 | 4 |
| gi\|7248379 | SH3P7r3 [Rattus norvegicus] | 22,7 | 4 |
| gi\|186910265 | signal recognition particle 9 [Rattus norvegicus] | 27,9 | 2 |
| gi\|6981592 | signal transducer and activator of transcription 3 [Rattus norvegicus] | 13 | 3 |
| gi\|149052177 | similar to 39S ribosomal protein L28, mitochondrial precursor (L28mt) (MRP-L28) [Rattus norvegicus] | 30 | 2 |
| gi\|187469118 | Similar to 60S ribosomal protein L12 [Rattus norvegicus] | 46,7 | 2 |
| gi\|68533996 | Similar to ADP-ribosylation factor GTPase-activating protein 3 (ARF GAP 3) [Rattus norvegicus] | 8,4 | 2 |
| gi\|78395043 | Similar to Basic FGF-repressed Zic-binding protein (mbFZb) [Rattus norvegicus] | 21,7 | 2 |
| gi\|167736386 | similar to DNA segment, Chr 4, ERATO Doi 22, expressed isoform 1 [Rattus norvegicus] | 21,1 | 2 |
| gi\|149032962 | similar to Laminin alpha-4 chain precursor (predicted), isoform CRA_d [Rattus norvegicus] | 13,3 | 2 |
| gi\|149038801 | similar to LIM and senescent cell antigen-like domains 1 (predicted), isoform CRA_b [Rattus norvegicus] | 26,8 | 6 |

| | | | |
|---|---|---|---|
| gi\|77748329 | Similar to Protein C6orf203 [Rattus norvegicus] | 32,9 | 5 |
| gi\|149028159 | similar to Putative ATP-dependent Clp protease proteolytic subunit, mitochondrial precursor (Endopeptidase Clp) [Rattus norvegicus] | 20,4 | 4 |
| gi\|149018745 | similar to RIKEN cDNA 1300017J02, isoform CRA_c [Rattus norvegicus] | 24,5 | 5 |
| gi\|187469709 | Similar to RIKEN cDNA 2010011I20 [Rattus norvegicus] | 22,1 | 2 |
| gi\|149018298 | similar to RIKEN cDNA 2010110K16 (predicted), isoform CRA_b [Rattus norvegicus] | 29,3 | 2 |
| gi\|149055499 | similar to RIKEN cDNA 9430083G14, isoform CRA_a [Rattus norvegicus] | 42,6 | 9 |
| gi\|149067647 | similar to tripartite motif protein 50 (predicted) [Rattus norvegicus] | 43,4 | 15 |
| gi\|215819922 | SIRT3L mitochondrial precursor [Rattus norvegicus] | 30,9 | 4 |
| gi\|149056444 | sirtuin (silent mating type information regulation 2 homolog) 2 (S. cerevisiae), isoform CRA_b [Rattus norvegicus] | 22,8 | 2 |
| gi\|4958935 | SKD3 [Rattus norvegicus] | 21,4 | 2 |
| gi\|47718004 | Slc25a3 protein [Rattus norvegicus] | 41,2 | 37 |
| gi\|3309068 | small GTP-binding protein rab5 [Rattus norvegicus] | 32,1 | 4 |
| gi\|6457612 | small heat shock protein B3 [Rattus norvegicus] | 40,8 | 4 |
| gi\|5107153 | small zinc finger-like protein [Rattus norvegicus] | 40,2 | 6 |
| gi\|6467898 | small zinc finger-like protein DDP2 [Rattus norvegicus] | 28,9 | 3 |
| gi\|187469313 | Snrp70 protein [Rattus norvegicus] | 10,4 | 2 |
| gi\|169642767 | Snx5 protein [Rattus norvegicus] | 26 | 4 |
| gi\|6981542 | solute carrier family 16, member 1 (monocarboxylic acid transporter 1) [Rattus norvegicus] | 19,4 | 9 |
| gi\|38014819 | Solute carrier family 25 (mitochondrial carrier; adenine nucleotide translocator), member 4 [Rattus norvegicus] | 75,8 | 75 |
| gi\|50054324 | solute carrier family 27, member 1 [Rattus norvegicus] | 17,5 | 2 |
| gi\|78214331 | solute carrier family 8, member 1 precursor [Rattus norvegicus] | 11,4 | 4 |
| gi\|187469715 | Sorting nexin 2 [Rattus norvegicus] | 20,6 | 4 |
| gi\|149044856 | spectrin beta 2, isoform CRA_a [Rattus norvegicus] | 36,2 | 36 |
| gi\|71480173 | spectrin repeat containing, nuclear envelope 1 [Rattus norvegicus] | 16,3 | 3 |
| gi\|56090475 | S-phase kinase-associated protein 1 [Rattus norvegicus] | 42,3 | 6 |
| gi\|157818019 | splicing factor, arginine/serine-rich 1 [Rattus norvegicus] | 56,9 | 4 |
| gi\|77404395 | staphylococcal nuclease domain containing 1 [Rattus norvegicus] | 28,4 | 6 |
| gi\|8393696 | stathmin 1 [Rattus norvegicus] | 44,3 | 3 |
| gi\|6981598 | steryl-sulfatase precursor [Rattus norvegicus] | 11,3 | 2 |

| | | | |
|---|---|---|---|
| gi\|158081745 | STIP1 homology and U-Box containing protein 1 [Rattus norvegicus] | 22,7 | 2 |
| gi\|72255527 | stomatin (Epb7.2)-like 2 [Rattus norvegicus] | 26,6 | 6 |
| gi\|209915614 | succinate dehydrogenase complex, subunit B, iron sulfur (Ip) precursor [Rattus norvegicus] | 52,5 | 24 |
| gi\|158749584 | succinate-Coenzyme A ligase, ADP-forming, beta subunit [Rattus norvegicus] | 67,1 | 34 |
| gi\|51260799 | Suclg2 protein [Rattus norvegicus] | 44,9 | 25 |
| gi\|211828197 | Sulfite oxidase [Rattus norvegicus] | 23,8 | 4 |
| gi\|8394328 | superoxide dismutase 1, soluble [Rattus norvegicus] | 56,5 | 22 |
| gi\|8394331 | superoxide dismutase 2, mitochondrial precursor [Rattus norvegicus] | 38,7 | 23 |
| gi\|50927605 | Suppression of tumorigenicity 13 [Rattus norvegicus] | 38 | 13 |
| gi\|157822537 | SWAP-70 protein [Rattus norvegicus] | 21,5 | 2 |
| gi\|149035828 | synapse associated protein 1, isoform CRA_a [Rattus norvegicus] | 14,7 | 2 |
| gi\|77695930 | syntaxin 12 [Rattus norvegicus] | 15,6 | 2 |
| gi\|201023317 | syntrophin, acidic 1 [Rattus norvegicus] | 28,7 | 2 |
| gi\|194474040 | syntrophin, beta 1 [Rattus norvegicus] | 32,1 | 8 |
| gi\|58865526 | TAR DNA binding protein [Rattus norvegicus] | 19,7 | 2 |
| gi\|149032481 | target of myb1 homolog (chicken), isoform CRA_a [Rattus norvegicus] | 18,4 | 2 |
| gi\|209529675 | taxilin beta [Rattus norvegicus] | 31,6 | 8 |
| gi\|6981642 | t-complex 1 [Rattus norvegicus] | 21,9 | 7 |
| gi\|3983131 | testicular N-cadherin [Rattus norvegicus] | 12,9 | 6 |
| gi\|53850588 | tetratricopeptide repeat domain 1 [Rattus norvegicus] | 25,7 | 2 |
| gi\|4325177 | thiopurine S-methyltransferase [Rattus norvegicus] | 27,9 | 4 |
| gi\|34849734 | Thioredoxin 1 [Rattus norvegicus] | 41 | 7 |
| gi\|209915579 | thioredoxin domain containing 13 [Rattus norvegicus] | 25 | 4 |
| gi\|55250718 | Thioredoxin reductase 2 [Rattus norvegicus] | 15,2 | 3 |
| gi\|157823439 | tight junction protein 1 [Rattus norvegicus] | 14,2 | 3 |
| gi\|198278573 | TIP41, TOR signaling pathway regulator-like [Rattus norvegicus] | 19,9 | 3 |
| gi\|7106246 | tissue-nonspecific alkaline phosphatase precursor [Rattus norvegicus] | 10,3 | 3 |
| gi\|84781723 | TNF receptor-associated protein 1 precursor [Rattus norvegicus] | 36,7 | 13 |
| gi\|8393848 | trans-2-enoyl-CoA reductase, mitochondrial precursor [Rattus norvegicus] | 31,1 | 4 |

| | | | |
|---|---|---|---|
| gi\|4914683 | transactivating protein BRIDGE [Rattus norvegicus] | 46,9 | 4 |
| gi\|38304015 | Transcription factor A, mitochondrial [Rattus norvegicus] | 20,1 | 4 |
| gi\|1854476 | transferrin [Rattus norvegicus] | 46,9 | 56 |
| gi\|52486810 | transforming acidic coiled coil 2 isoform 1 [Rattus norvegicus] | 8,4 | 2 |
| gi\|603877 | transgelin [Rattus norvegicus] | 40,8 | 7 |
| gi\|149040761 | transgelin 2, isoform CRA_b [Rattus norvegicus] | 28,4 | 6 |
| gi\|641973 | transitional endoplasmic reticulum ATPase [Rattus norvegicus] | 38 | 28 |
| gi\|485267 | transketolase [Rattus norvegicus] | 11,3 | 4 |
| gi\|60688577 | Translocase of inner mitochondrial membrane 10 homolog (yeast) [Rattus norvegicus] | 43,3 | 3 |
| gi\|77415403 | Translocase of outer mitochondrial membrane 34 [Rattus norvegicus] | 42,4 | 5 |
| gi\|149015618 | translocator of inner mitochondrial membrane 44, isoform CRA_b [Rattus norvegicus] | 42,2 | 15 |
| gi\|149047669 | transmembrane emp24 protein transport domain containing 4 (predicted), isoform CRA_b [Rattus norvegicus] | 18,1 | 2 |
| gi\|157819597 | transmembrane protein 143 [Rattus norvegicus] | 14 | 3 |
| gi\|6851387 | triadin 1 [Rattus norvegicus] | 20,2 | 2 |
| gi\|149046248 | tripeptidyl peptidase II, isoform CRA_a [Rattus norvegicus] | 13,2 | 7 |
| gi\|67968365 | TRK-fused gene protein [Rattus norvegicus] | 11,7 | 2 |
| gi\|66730475 | tropomyosin 2, beta [Rattus norvegicus] | 62,7 | 30 |
| gi\|8394469 | troponin 1, type 3 [Rattus norvegicus] | 59,2 | 18 |
| gi\|77627992 | troponin C type 1 (slow) [Rattus norvegicus] | 31,1 | 6 |
| gi\|203656 | troponin T [Rattus norvegicus] | 56,2 | 18 |
| gi\|62460630 | tryptophanyl-tRNA synthetase [Rattus norvegicus] | 23,4 | 2 |
| gi\|157823715 | tubulin polymerization promoting protein [Rattus norvegicus] | 22,9 | 2 |
| gi\|224839 | tubulin T beta15 | 51,2 | 26 |
| gi\|58865558 | tubulin, alpha 1C [Rattus norvegicus] | 54,8 | 34 |
| gi\|21666559 | TUC-4b [Rattus norvegicus] | 16,7 | 2 |
| gi\|210032365 | tumor rejection antigen gp96 precursor [Rattus norvegicus] | 22,9 | 12 |
| gi\|149031974 | type II keratin Kb15 [Rattus norvegicus] | 41,2 | 15 |
| gi\|6981710 | tyrosine 3-monooxygenase/tryptophan 5-monooxygenase activation protein, eta polypeptide [Rattus norvegicus] | 61,8 | 21 |
| gi\|9507245 | tyrosine 3-monooxygenase/tryptophan 5-monooxygenase activation protein, gamma polypeptide [Rattus norvegicus] | 57,9 | 19 |

| | | | |
|---|---|---|---|
| gi\|6981712 | tyrosine 3-monooxygenase/tryptophan 5-monooxygenase activation protein, theta polypeptide [Rattus norvegicus] | 35,9 | 6 |
| gi\|71043618 | tyrosyl-tRNA synthetase [Rattus norvegicus] | 32,8 | 6 |
| gi\|73695428 | Ubc protein [Rattus norvegicus] | 86,3 | 11 |
| gi\|187469561 | Ube2l3 protein [Rattus norvegicus] | 26,6 | 4 |
| gi\|55741544 | ubiquinol cytochrome c reductase core protein 2 precursor [Rattus norvegicus] | 58,9 | 55 |
| gi\|189011657 | ubiquinol-cytochrome c reductase binding protein [Rattus norvegicus] | 64 | 30 |
| gi\|281427170 | ubiquinol-cytochrome c reductase complex 7.2kDa protein [Rattus norvegicus] | 45,3 | 5 |
| gi\|57114330 | ubiquinol-cytochrome c reductase, Rieske iron-sulfur polypeptide 1 [Rattus norvegicus] | 43,1 | 27 |
| gi\|157819971 | ubiquitin specific peptidase 5 [Rattus norvegicus] | 21,7 | 9 |
| gi\|56605688 | ubiquitin specific protease 14 [Rattus norvegicus] | 25,6 | 4 |
| gi\|56270304 | Ubiquitin-conjugating enzyme E2G 1 (UBC7 homolog, C. elegans) [Rattus norvegicus] | 20 | 2 |
| gi\|157817518 | ubiquitin-conjugating enzyme E2M (UBC12 homolog, yeast) [Rattus norvegicus] | 39,9 | 4 |
| gi\|197384571 | ubiquitin-like modifier activating enzyme 2 [Rattus norvegicus] | 19,9 | 2 |
| gi\|67078526 | UDP-glucose pyrophosphorylase 2 [Rattus norvegicus] | 25,6 | 5 |
| gi\|71043752 | UMP-CMP kinase 1 [Rattus norvegicus] | 35,2 | 5 |
| gi\|157820137 | unc-45 homolog B [Rattus norvegicus] | 23,4 | 5 |
| gi\|95115832 | unknown [Rattus norvegicus] | 18,6 | 4 |
| gi\|829028 | unknown protein [Rattus norvegicus] | 10 | 2 |
| gi\|55628 | unnamed protein product [Rattus norvegicus] | 75,7 | 192 |
| gi\|763179 | unnamed protein product [Rattus norvegicus] | 69,5 | 20 |
| gi\|57087 | unnamed protein product [Rattus norvegicus] | 57,6 | 5 |
| gi\|55985 | unnamed protein product [Rattus norvegicus] | 48,8 | 13 |
| gi\|56905 | unnamed protein product [Rattus norvegicus] | 41,9 | 14 |
| gi\|56336 | unnamed protein product [Rattus norvegicus] | 41,9 | 5 |
| gi\|57294 | unnamed protein product [Rattus norvegicus] | 41,6 | 16 |
| gi\|57139 | unnamed protein product [Rattus norvegicus] | 21,2 | 3 |
| gi\|55729 | unnamed protein product [Rattus norvegicus] | 18,9 | 4 |
| gi\|56773 | unnamed protein product [Rattus norvegicus] | 15,9 | 4 |
| gi\|57125 | unnamed protein product [Rattus norvegicus] | 15,5 | 4 |

| | | | |
|---|---|---|---|
| gi\|259668299 | unnamed protein product [Rattus norvegicus] | 15,3 | 2 |
| gi\|56334 | unnamed protein product [Rattus norvegicus] | 13,8 | 2 |
| gi\|56494 | unnamed protein product [Rattus norvegicus] | 13,1 | 3 |
| gi\|197333853 | vacuolar protein sorting 26 homolog B [Rattus norvegicus] | 22 | 2 |
| gi\|189163489 | vacuolar protein sorting 36 homolog [Rattus norvegicus] | 24,6 | 2 |
| gi\|4240462 | VAMP-associated protein A [Rattus norvegicus] | 32,2 | 7 |
| gi\|4240464 | VAMP-associated protein B [Rattus norvegicus] | 30 | 3 |
| gi\|71043730 | vanin 1 [Rattus norvegicus] | 13,9 | 3 |
| gi\|9506515 | v-crk sarcoma virus CT10 oncogene homolog [Rattus norvegicus] | 28,6 | 8 |
| gi\|57480 | vimentin [Rattus norvegicus] | 44,4 | 9 |
| gi\|149031250 | vinculin (predicted), isoform CRA_a [Rattus norvegicus] | 45,9 | 39 |
| gi\|8810245 | voltage-dependent anion channel 1 [Rattus norvegicus] | 79,9 | 54 |
| gi\|8810247 | voltage-dependent anion channel 2 [Rattus norvegicus] | 44,4 | 11 |
| gi\|38512108 | Voltage-dependent anion channel 3 [Rattus norvegicus] | 53 | 13 |
| gi\|8394544 | xanthine dehydrogenase [Rattus norvegicus] | 11,6 | 3 |
| gi\|65301494 | X-linked eukaryotic translation initiation factor 1A [Rattus norvegicus] | 35,4 | 2 |
| gi\|92373398 | Y box binding protein 1 [Rattus norvegicus] | 51,6 | 12 |
| gi\|95102024 | ZH14 [Rattus norvegicus] | 35,3 | 5 |
| gi\|57506 | zinc binding protein [Rattus norvegicus] | 34 | 3 |
